# The geometry of the Pareto front in biological phenotype space

**DOI:** 10.1002/ece3.528

**Published:** 2013-04-17

**Authors:** Hila Sheftel, Oren Shoval, Avi Mayo, Uri Alon

**Affiliations:** Department of Molecular Cell Biology, Weizmann Institute of Science234 Herzl Street, Rehovot, 76100, Israel

**Keywords:** Ecological morphology, efficiency front, evolutionary theory, evolutionary trade-offs, location theory, multi-objective optimality

## Abstract

When organisms perform a single task, selection leads to phenotypes that maximize performance at that task. When organisms need to perform multiple tasks, a trade-off arises because no phenotype can optimize all tasks. Recent work addressed this question, and assumed that the performance at each task decays with distance in trait space from the best phenotype at that task. Under this assumption, the best-fitness solutions (termed the Pareto front) lie on simple low-dimensional shapes in trait space: line segments, triangles and other polygons. The vertices of these polygons are specialists at a single task. Here, we generalize this finding, by considering performance functions of general form, not necessarily functions that decay monotonically with distance from their peak. We find that, except for performance functions with highly eccentric contours, simple shapes in phenotype space are still found, but with mildly curving edges instead of straight ones. In a wide range of systems, complex data on multiple quantitative traits, which might be expected to fill a high-dimensional phenotype space, is predicted instead to collapse onto low-dimensional shapes; phenotypes near the vertices of these shapes are predicted to be specialists, and can thus suggest which tasks may be at play.

## Introduction

Biological systems often need to perform more than one task. A given design or shape – that is, a phenotype – cannot usually be optimal at all tasks at the same time. This situation gives rise to a fundamental trade-off (Arnold 1983). Such trade-offs have been widely studied in ecology; examples include life history aspects such as fertility versus offspring survival (Stearns 1992), and performance measures such as speed versus endurance in lizards (Vanhooydonck et al. 2001), foraging scale versus precision (Campbell et al. 1991) and growth versus shell robustness in snails (Trussell 2000). The broad context of this study is to ask how such trade-offs affect the range of phenotypes found in nature.

Recently, Pareto optimality was used to understand the range of phenotypes that best resolve such trade-offs (Shoval et al. 2012). To define Pareto optimality, consider a system with n traits (quantitative traits such as size and shape parameters). A phenotype *v* is a vector of trait values, and can be described as a point in morphospace – the space of trait values.

Assume that the system needs to perform *k* different tasks. The phenotype's performance *p*_*i*_(*v*) at each task *i* is a function of its trait values, *v*. The fitness of the organism, *F*(*v*), is an increasing function of its performance at each task *F*(*v*) = *f*_*h*_(*p*_1_(*v*), *p*_2_(*v*), …, *p*_*k*_(*v*)) (Arnold 1983). The function *f*_*h*_ describes the relative importance of the performance of each task in determining the fitness in niche *h*. In the following, we do not need to know the explicit form of *f*_*h*_, only that it increases with performances. Note the difference between performance and fitness: the fitness function *f*_*h*_ is associated with a given niche and determines which phenotype will be selected at that niche. The fitness combines the different performances in a way that is relevant to that given niche. The performance functions are global and do not depend on the niche. It is usually easier to experimentally measure performances in the lab than fitness in the wild (Arnold 1983).

**Figure 1:**
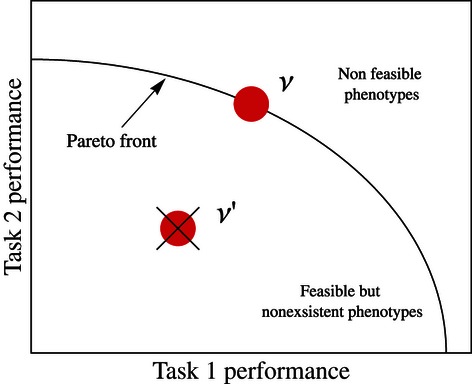
Schematic view of the Pareto front in performance space. The Pareto front is the set of phenotypes that remain after all phenotypes are removed that are dominated in all tasks by another phenotype. Note that this is plotted in performance space and not trait space.

Pareto optimality is usually defined in performance space (schematically shown in Fig. 1). If phenotype *v* has higher performance at all tasks than phenotype *v’*, one can erase *v’*. Eliminating all such *v’* phenotypes results in the Pareto front. Moving along the front leads to improvement in some tasks at the expense of others. The front is the set of best compromises. Note that natural selection tends to select phenotypes on the Pareto front (or close to the front, the closer the higher the selection pressure), rather than phenotypes that are off the front (Oster and Wilson 1979; Farnsworth and Niklas 1995; El Samad et al. 2005; Kennedy 2009; Warmflash et al. 2012). This is due to the fact that fitness is an increasing function of each of the performances. Each niche *h* corresponds to a different point on the front, determined by the relative importance of the different tasks in that niche, as defined by the particular form of the fitness function *f*_*h*_.

Most studies of Pareto optimality in economics and engineering focus on performance space (Steuer 1986). Few studies explore the trait space (morphospace), as we do in this article. An exception is location theory that studies optimizing functions of a distance from given points (Kuhn 1967; Thisse et al. 1984; Durier and Michelot 1986).

Shoval et al. (2012) calculated the shape of the Pareto front in morphospace. To do so, three assumptions were made. The main aim of this study is to explore the effects of relaxing these assumptions. The first assumption is that for each task *i*, there is a single phenotype 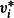 that maximizes the corresponding performance function *Pi*. This phenotype is called the *archetype* for task *i*. Relaxing this assumption means that performance can be maximized at multiple points.

The second assumption is that the performance of a phenotype is a decreasing function of its distance from the archetype for that task: *P*_*i*_(*v*) = *p*_*i*_(*d*_*i*_(*v*)), where 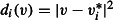 and 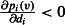. The important point here is the existence of a distance metric, more specifically an inner-product norm distance on the morphospace. This distance function governs the decrease of the performance functions. An inner-product norm is defined by 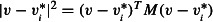, where *M* is a positive-definite matrix. One example for such a norm is Euclidian distance (given by *M* = *I*, the identity matrix). Relaxing this assumption means that performance decays not with a distance metric away from its maximum.

The third assumption was that all performance functions decay with the *same* norm from their maxima. Relaxing this assumption means that each performance decays with a different norm.

**Figure 2:**
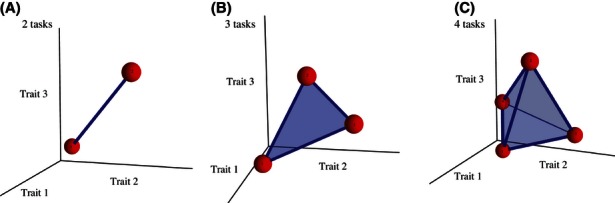
Pareto fronts are simple shapes in trait space according to the assumptions of Shoval et al. Under the assumptions of (1), the Pareto front is the convex hull of the archetypes – the phenotypes that maximize performance of one of the tasks. For two tasks, the front is the line segment connecting the two archetypes. For three tasks, it is the full triangle whose vertices are the three archetypes. Tetrahedrons are found for four tasks. Archetypes are denoted by red circles. Note the difference between the morphospace depiction in this figure, where axes are traits, and the performance space description in Fig. 1, where axes are performances.

Under these assumptions, it was shown (Shoval et al. 2012) that the Pareto front is the convex hull of the archetypes. In other words, phenotypes on the Pareto front are linear combinations of the *k* archetypes: 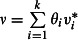, with nonnegative coefficients 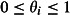, that sum to one 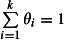. The Pareto front for two tasks is a line segment that connects the two archetypes; three tasks result in a triangle shaped Pareto front. Four tasks result in a tetrahedron, etc. (see Fig. 2). These results generalize previous theorems in location theory, such as (Kuhn 1967), which considered only Euclidean norms, and did not make a connection with biological evolution. Consequently, no matter how large the number of traits in the system – as long as they correspond to tasks that show trade-offs – the theory predicts that naturally selected phenotypes fall on a low-dimensional space, and within that space on a polytope (line, triangle, etc.). The vertices of the polytope are the archetypes. In practice, one can fit a polytope to the data, and discover the potential archetypes, which are the vertices of the polytope. The niches or behaviors of the species in the dataset closest to the archetypes give clues as to what tasks might be at play. Evidence for such lines and triangles was presented by Shoval et al. based on classic studies of animal morphology, and bacterial gene expression datasets.

Here we ask what happens if we relax these assumptions. The article is organized as follows: we first relax assumption (iii), to consider a different inner-product norm for each performance function. We then relax assumption (i) to consider cases where performance is maximized in a region and not at a single point. Finally, we relax all assumptions, and consider general performance functions that need not be monotonic or depend on a norm.

Our main conclusions are that the shape of the Pareto front for the case of different norms is composed of mildly curved hyperbolae. We also present a theorem that places bounds on the Pareto front in cases of general, non-monotonic performance functions. Generally, relaxing the assumptions of Shoval et al. changes the straight edges of the polytopes to mildly curved ones. Table 1 lists the results in this study that go beyond the study of Shoval et al. (Shoval et al. 2012).

**Table 1:** Findings of presence study that extend the results of Shoval et al. (2012)

	Shoval et al. 2012	Present study
Shape of Pareto fronts for two tasks in a 2-dimensional morphospace with different inner-product norms	Numerical calculation showed that shape can be curved (Figs. 3D and S2)	The curve is analytically solved, found to be a hyperbola (Appendix S2)
Shape of Pareto fronts for two tasks in an N-dimensional morphospace with different inner-product norms	Not discussed	Is calculated along with 2D projections. Axes can be rotated such that all projections on principal planes are hyperbolae. (Appendix S3)
The maximal deviation of the Pareto front from a straight line	Was calculated numerically (Fig. S3)	Is calculated analytically. Bounds are provided. (Appendix S4)
Pareto fronts for three tasks with different norms are curved triangles or multi-connected regions	Mentioned.	Proved (Appendix S7)
Relaxing the assumption that the Pareto front is maximized at a single point	Discussed for the case of two tasks and performance that decays with Euclidean norm	The case of three tasks is discussed (Appendix S8)
Inverse problem of deducing the norms from the shape of the Pareto front	Not studied	Studied for 2 and 3 tasks in 2D (Appendices S5 and S6)
Bounds for the Pareto front in the case of general performance functions (not decaying with a norm, not necessarily monotonic)	Not studied	Proved to be restricted to a region near the archetypes (Appendix S9)

## Results

### Pareto fronts for two tasks with different inner-product norms are hyperbolae

We start with relaxing the assumption that all performance functions decay with the *same* inner-product norm from their maxima. It is important to relax this assumption, because in reality, performance at task 1 may depend more strongly on certain traits, whereas performance at task 2 may depend most strongly on other traits. The result is performance contours that have different shapes for the two performance functions.

Shoval et al. found that for two tasks with performance functions that decay with the same inner-product norm, the Pareto front is the straight-line segment that connects the two archetypes. In this section, we relax the assumption that the norms for the two tasks are the same.

We use inner-product norms for two reasons: first, one can obtain analytical results that are relatively easy to interpret. Second, performance functions generally decay, close to their maximum, with an inner-product distance metric. To see this, consider the second order Taylor expansion of a function *P*_*i*_(*v*) near its maximum 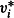, namely 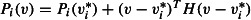, where H is the Hessian matrix (matrix of second order partial derivatives evaluated at 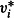) which is negative definite (unless Det*(H)* = 0). Thus, near the maximum, the function decays according to an inner-product metric with positive-definite matrix *M = −H*.

**Figure 3:**
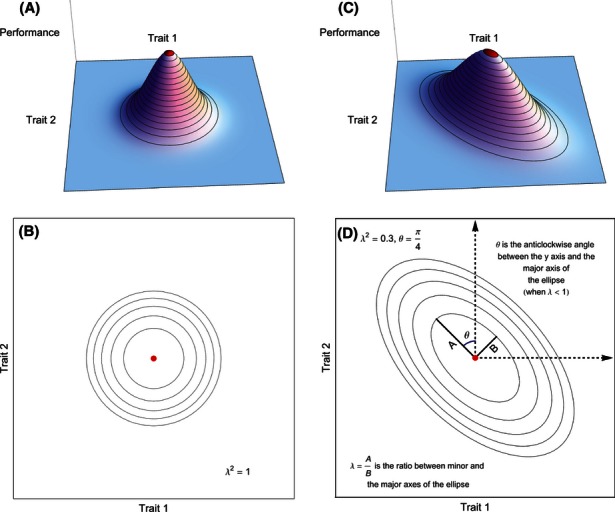
Performance functions that decay with inner-product norms and their contours. Performance functions with a single maximum and which decay with an inner-product norm distance away from that maximum have contours in the shape of circles or ellipses. (A) A 3D plot of a performance function that depends on two traits and decays with Euclidean distance from its maximum. (B) The contours of the performance function shown in (A) on the plane defined by the traits. The contours are concentric circles. (C) A 3D plot of a performance function that depends on two traits, and decays with a non-Euclidean, inner-product norm from its maximum. (D) The contours of the function shown in (C) are concentric ellipses, whose shape is determined by the parameters *λ* and *θ* defined in the figure. In (A–D) maximum points are marked by red dots, contours by solid lines.

An example of performance functions with different inner-product norms is shown in Figure 3. The contours of these functions are concentric parallel ellipses; they are circles in the case of of Euclidean norm (Fig. 3B). These contour families can be defined by two parameters, the angle θ between the major axis of the ellipse and the *y*-axis of morphospace, and λ the ratio between the minor and major axes of the ellipse (Fig. 3D).

To calculate the Pareto front, we note that the front is the locus of all points at which contours of the two performance functions are externally tangent to each other (for a proof see Appendix S2). The proof shows, briefly, that if a point *x* is not on this locus, there exist nearby points, which have higher performance in both tasks–and thus *x* is not on the Pareto front.

**Figure 4:**
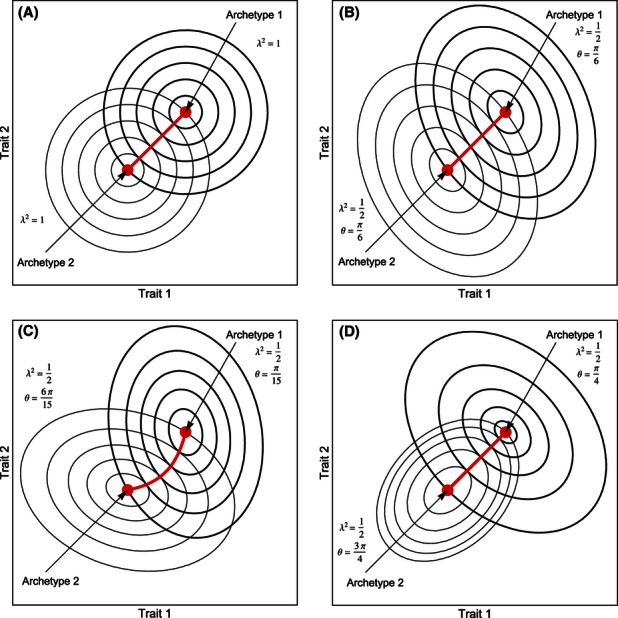
The Pareto front in a 2D morphospace resulting from two tasks with two different norms is a hyperbola or a straight line. (A, B) When the two norms are equal, either Euclidean (A) or with a general elliptical contour (B), the front is a straight-line segment. (C, D) When the two norms are different the Pareto front is a hyperbola that connects the two archetypes, except in special cases such as (D) in which the two different inner-product norms have a main axis that is perpendicular to the line between the archetypes. In this case, the Pareto front is a straight line connecting the two archetypes. In (A–D), the front is a marked by a red line, and the archetypes are denoted by red dots. The contours of the performance functions are plotted in black and gray. Notice that the Pareto front is given by the locus of points at which the contours of the two performance functions are externally tangent.

When the two norms are different, we find that the Pareto front in a two-dimensional morphospace is a hyperbola segment that connects the two archetypes (Fig. 4C, Appendix S2 for proof). The parameters of the hyperbola can be calculated from the parameters of the norms *θ* and *λ*. We find that in specific cases, the hyperbolic Pareto front becomes a straight line even when the norms are different – namely when one of the axes of each ellipse aligns with the line connecting the archetypes (Fig. 4D).

**Figure 5:**
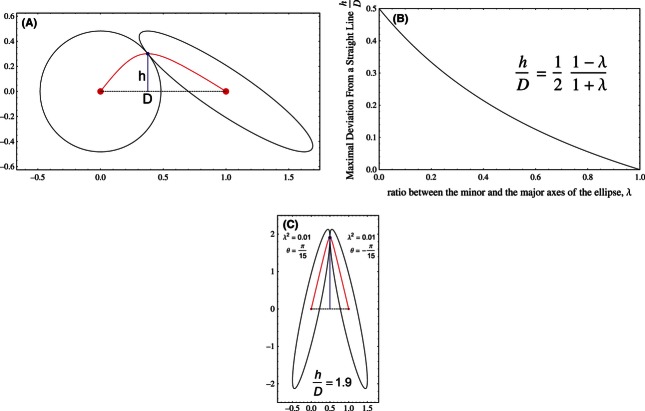
The maximal deviation of the Pareto front from a straight line is mild for most norms. (A) The deviation of the Pareto front (red curve) from the straight line connecting the archetypes (red dots) is defined by the maximal height *h* of a point on the front relative to that line, divided by the distance between the archetypes, *D*. The point that maximizes the deviation is plotted as a blue dot. (B) When one of the performance functions depends on a Euclidean norm and the second performance function depends on a general inner-product norm with parameters *θ*, *λ* as defined in Fig. 2, 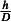 is bounded by 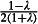. When setting 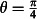, this maximum is obtained. The graph shows the maximal deviation as a function of *λ*. (C) Only when the two norms are very eccentric can the front show large deviations from a straight line.

We next studied the maximal deviation of the Pareto front from a straight line, measured as the maximal Euclidean distance *h* from the straight line connecting the archetypes divided by the distance between the archetypes, *D* (Fig. 5A). When one norm is Euclidian (circular contours) and the other has elliptic contours, the maximal deviation occurs when the ellipse is at an angle of 45° relative to the line between archetypes, and is given by 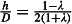. The Pareto front makes only small deviations from a line when the ellipse is mildly eccentric – for example, when the ellipse axis ratio is 2:1, the Pareto front deviates from a line by about 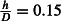 (Fig. 5B). The deviation grows with ellipse axis ratio and is bounded by 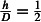 (as *λ* → 0).

When contours of both performance functions are highly eccentric, larger deviations can occur. These deviations are bounded by 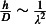 in the limit where both ellipses have equal *λ* and are very eccentric, *λ* → 0 (proofs in Appendix S4). Thus, deviations from a straight line are generally mild, except in the case of highly eccentric norms.

**Figure 6:**
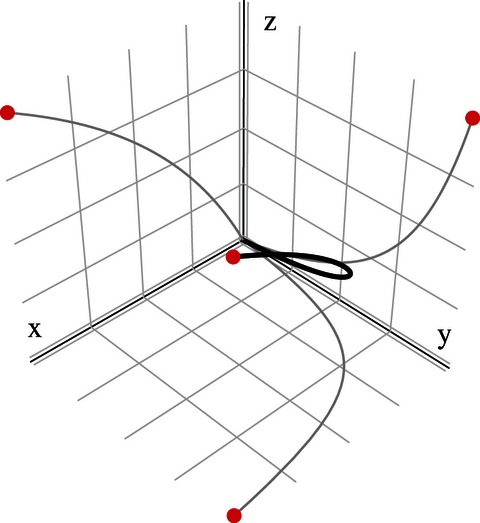
The two-task Pareto front in an n-dimensional morphospace is a one-dimensional curve connecting the two archetypes. There exists a basis for the morphospace such that the projection of the Pareto front on every plane spanned by two basis vectors is a hyperbola, or in special cases, a line. Here, a 3D example is shown with the norms given by the matrices 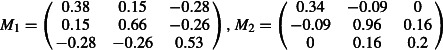. The Pareto front is plotted in black; the projections on the XY, YZ and XZ planes are in gray. The archetypes and their projections are plotted as red circles. Note that generally the front itself does not lie on any 2D plane. X,Y and Z axes are linear combination of the traits. Specifically here, X,Y and Z are chosen as the orthogonal basis in which the projections of the Pareto front on the planes spanned by each two basis vectors are hyperbolae.

In higher dimensions, the Pareto front for two tasks is a one-dimensional curve between the two archetypes. The projections of this curve are hyperbolic in the following sense: there exists a coordinate system in which the projection of this curve on every principal plane (plane spanned by two of the axes) is a hyperbola (or, in specific cases, a line)—see Fig. 6 (proof in Appendix S3).

### Pareto fronts for three tasks with different norms are curved triangles or multi-connected regions

We next consider the case of three tasks. For equal norms, Shoval et al. showed that the Pareto front is the full triangle whose vertices are the archetypes. We consider the case where the norms are different. We begin with a 2D morphospace. Each performance function has its own set of elliptical contours, with differently shaped ellipses for each task.

We calculated the shapes of the Pareto front analytically (Appendix S1). We find that the Pareto front is a region bounded by hyperbolae segments that connect the archetypes. These hyperbolae are identical to the Pareto fronts associated with the three pairs of tasks (Appendix S7).

**Figure 7:**
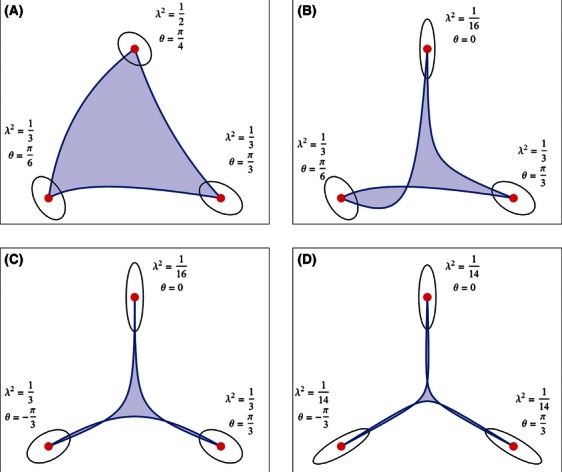
The three-task Pareto front in a 2D morphospace is a region enclosed by three hyperbolae. The result is a triangle-like shape with hyperbolic edges (A), or multiple connected regions, depending on the intersections between the hyperbolae. There thus exist classes of fronts, with two (B), three (C) or four (D) connected components. These three classes can occur when one or more of the norms have high eccentricity. Norm parameters are shown in the figure.

The hyperbolic edges can form four topological classes of Pareto front shapes. The first class of Pareto fronts resembles curved triangles, with three hyperbolic edges (Fig. 7A). The other classes have two, three, and four components (Fig. 7B–D). These last three classes occur when the hyperbola segments intersect at points other than the archetypes. Such intersections may happen when at least one of the norms has high eccentricity (i.e., a trait combination that is significantly more influential on one performance than on others).

**Figure 8:**
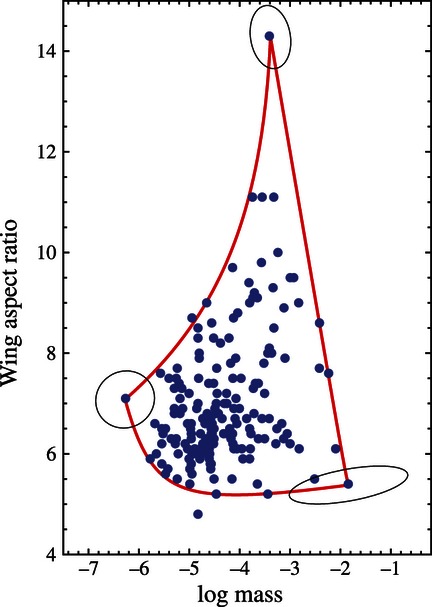
Wing aspect ratio versus mass of 108 bat (Microchiroptera) species. A Pareto front bounded by three hyperbolae (red) was manually fitted to the data (blue dots). The front is generated by three different inner-product norms, one for each task. Note that there is a one-dimensional family of other norms that can give the same front shape.

We demonstrate this by using a morphological dataset analyzed by Shoval et al., regarding bat wing shape of 108 bat species (Norberg and Rayner 1987). The dataset can be fit quite well by a hyperbolae-edged triangle (Fig. 8).

**Figure 9:**
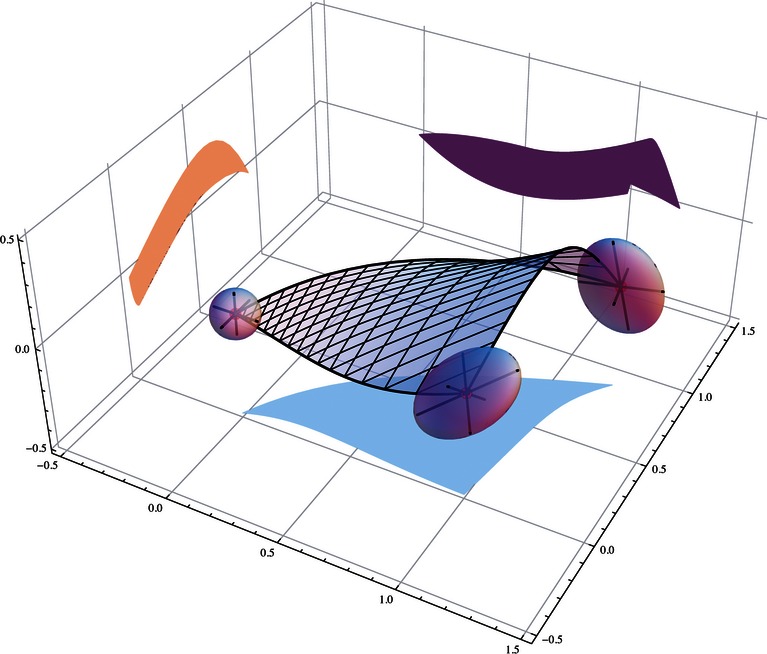
An example of a three-task Pareto front in a 3D morphospace. In this case the Pareto front is a 2D manifold that resembles a distorted triangular shape, whose vertices are the three archetypes. Note that the 2D projections (plotted in the figure on the XY, YZ and XZ planes) do not generally have hyperbolic edges, and can have relatively complicated shapes. The three norms in this case are given by the matrices 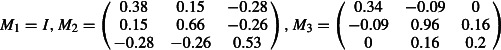

We also considered morphospaces of higher dimension (Appendix S1). We find that three tasks give rise to Pareto fronts that are curved two-dimensional surfaces. An example is shown in Fig. 9 for a 3D morphospace. In 3D, each task has equi-performance contours shaped as ellipsoids, centered on the archetype. The Pareto front in this case resembles a curved triangle.

The Pareto front remains connected and of low dimension even when considering a wider class of performance functions – strongly concave functions (Appendix S10).

It is interesting to consider what happens when one measures only some of the traits, for example two of the three traits that make up a 3D morphospace. This amounts to projecting the data on one of the principal planes. The projections of the curved Pareto front on principal planes resemble curved triangles in some cases, and more complex shapes in other cases. This means that when norms are different for each task, data on only two out of the three relevant traits might not fall within shapes predicted by a two-dimensional model.

In contrast, if the norms are equal for all tasks, one can safely measure only a subset of the traits: because in this case the front is a convex polygon, its projections are also convex polygons. Projecting a triangle in 3D on a 2D plane gives rise to a triangle (except in singular cases which give a line). Thus, two-dimensional data in this case give fronts that can be predicted from 2D models, even if there exist additional traits that are not measured. In summary, when norms are different, it may be difficult to interpret data that are missing important traits; when norms are approximately the same, interpretation of such data is easier.

**Figure 10:**
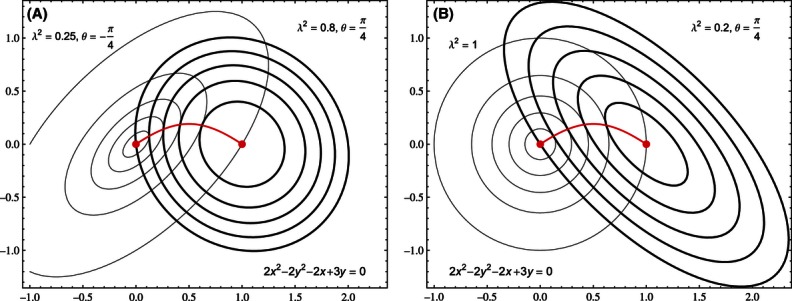
The norms cannot be uniquely deduced from the two-task hyperobla-shaped Pareto front. For two tasks in a 2D morphospace, the same hyperbola-shaped front can result from many pairs of norm (a 1D family of norm pairs). (A) and (B) show two different norm pairs (parameters given in the figure) that generate the same hyperbola, whose equation is shown in the figure. The archetypes are marked by red dots, the Pareto front is plotted in red, and the contours of the performance functions are in black and gray.

We also studied the inverse problem of deducing the different norms from the shape of the Pareto front. For two tasks, this inverse problem is not possible without additional information: a one-dimensional family of norm pairs can explain a given hyperbolic front (Fig. 10, Appendix S5). For three or more tasks, in 2D, the inverse problem is solvable in principle (Appendix S6). In practice, data must be very precise to differentiate between alternative norms that give very similar hyperbolic edges.

### Relaxing the assumption that the Pareto front is maximized at a single point

We now relax the assumption that the Pareto front is maximized at a single point (assumption i). We consider performance functions maximized in a region of morphospace rather than at a single point. This phenomenon, called many-to-one mapping or performance ridges, has been suggested to occur in biological systems (Schluter and Nychka 1994; Wainwright et al. 2005).

**Figure 11:**
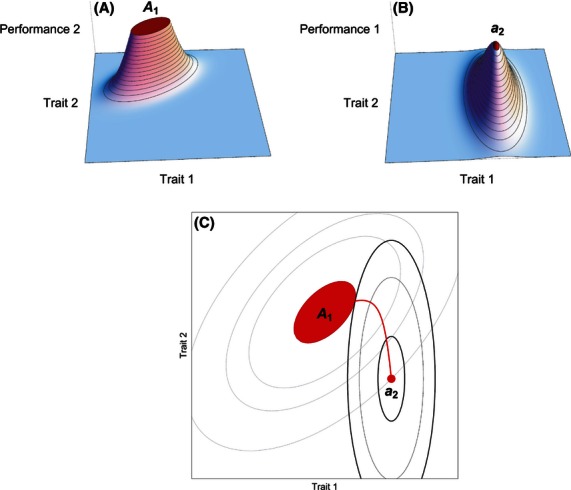
Pareto front when performance function is maximized in a region rather than a point. When performance at task 1 is maximal in a region of space (A_1_) rather than at a single point, the front is a curve connecting the second archetype *a*_2_ with the point on A_1_ that has maximal performance of task 2 – the point where a performance contour of task 2 first touches A_1_. (A) A plot of performance function 1 that is maximized in a region *A*_1_ (in red). (B) A plot of performance function 2 maximized at a point *a*_2_. (C) The Pareto front (red curve) connects *a*_2_ (red dot) to the point with maximal performance of task 2 on A_1_ (red region). Contours of performance 1 are in thin black, contours of performance 2 are in thick black.

We begin with two tasks. As found in Shoval et al. (2012) for performance functions that decay with Euclidean distance away from the maximizing region, the Pareto front connects the point on each region that is closest to the other archetype. We considered inner-product norms, and a maximizing region which is bounded in one of the elliptical contours. We find that, the Pareto front connects the points in which the highest possible performance contour of task i touches the archetype region of task j (Fig. 11, Appendix S8).

In this way, multiple tasks break the symmetry of points within the archetype region. If only one task was required, evolution can drift within the archetype region, because all points have the same fitness (Wainwright et al. 2005).The multi-objective nature of multiple tasks leads to a differentiation between points of equivalent performance, and selects a particular point on the boundary of the archetype region that is closest to the other archetype.

**Figure 12:**
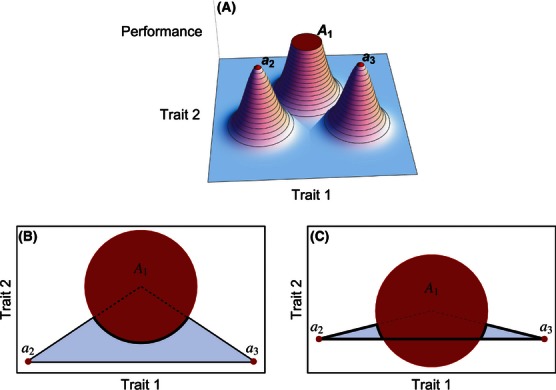
The Pareto front for three tasks, one maximized in a circle-shaped region, the others at points. (A) An example of a case where performance 1 is maximal in a region A_1_, and performance 2 and 3 are maximized by the points *a*_2_, *a*_3_, respectively. (B) When the two archetype points *a*_2_, *a*_3_ are connected by a line that does not intersect the archetype region of the third task (A_1_), the front includes the triangle whose third vertex is the center of the circle, minus the interior of the circle, but including an arc on its circumference. (C) A similar situation results when the line *a*_2_, *a*_3_ intersects the circle.

We also explored the case of three tasks, not considered in Shoval et al. (2012). For simplicity, we present the case of a task with a circle-shaped archetype region A_1_, and two other tasks with point-like archetypes a_2_ and a_3_, and assume that performances decay away from the maximal regions with Euclidean norm. We find that the Pareto front is bounded by the line between a_2_ and a_3_, the lines between the closest point on A_1_ and each of the archetypes, and an arc on the boundary of A_1_ (Fig. 12).

More generally, the Pareto front is composed of points on the boundary of the archetype regions, together with points on the Pareto front calculated as if the archetype was maximized at a single point within the archetype region- more details are given in Appendix S8.

### Bounds for the Pareto front in the case of general performance functions

We finally relax the assumption that the performance functions decay according to norms, and thus consider the case of general, continuous performance functions. These functions have a global maximum, the archetype, but need not be monotonically decreasing. We provide bounds on the Pareto front.

**Figure 13:**
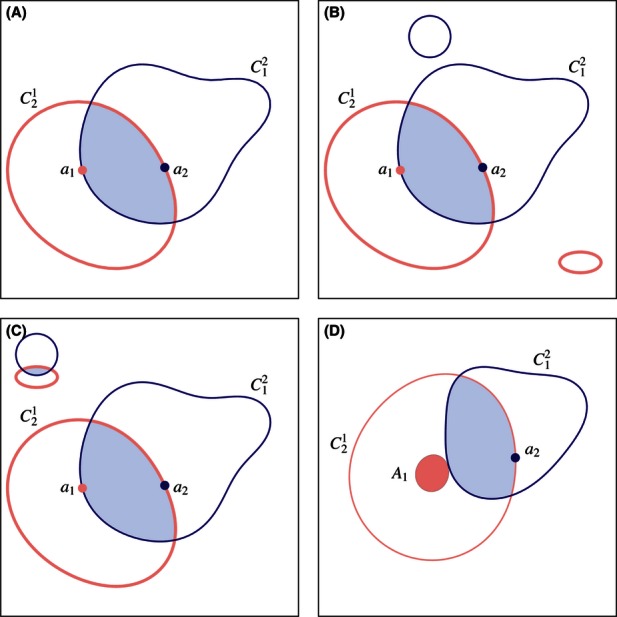
Bounds on the Pareto front for general performance functions show that it is located between the archetypes. (A) The Pareto front is bounded in a region (shaded) defined by the intersection of two special regions. The special region 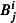 is defined by all points with performance in task *i* higher than the performance in task *i* of the archetype of task *j*, *a*_*j*_. The boundary of the each special region is the contour of the performance function *i* that crosses the archetype of performance *j*. (B) When performance functions are non-monotonic, the special regions 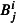 can have multiple non-connected regions, each surrounding a local maximum point. (C) If distant regions of the special contours intersect, the Pareto front can be localized to multiple intersection regions. (D) The same bound can apply when performance is optimized in a region, not a single point (region *A*_1_). In this case, the special region 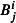 is defined by all points with performance at task *i* higher than the maximal performance in the archetype region of task *j*.

We begin with two tasks. We construct a special contour for each performance function- the contour that passes through the archetype of the other task. Thus, we construct the contour of performance function 1 that passes through archetype 2, denoted by 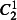, and the contour of performance function 2 that passes through archetype 1, denoted by 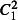 (Fig. 13). Define the interior 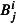 of a special contour 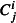 as the set of points in which performance *i* is greater or equal to its value on the contour. We find that the Pareto front is in the intersection of these interiors 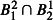 (see also Appendix S9).

This bound does not require that the performance functions be monotonic or have a single maximum. If local maxima of performance exist away from the archetype, there is a possibility that the special contour will be multiply connected: one piece of contour surrounding the archetype, and another piece surrounds each local maximum (as shown in Fig. 13). In this case, the bound suggests the possibility that parts of the Pareto front lie in distant regions in trait-space. However, such an occurrence requires a coincidence, namely that the local maxima of the two performance functions lie close to each other in morphospace. In the generic case, the present bound suggests that the Pareto front is localized to a restricted region in morphospace that lies between, and includes, the two archetypes. The size of the bound region is determined by the precise shape of the performance contours. Inside the bound region, the Pareto front is not necessarily connected. One can find performance functions in which the Pareto front is composed of several disjoint pieces (Appendix S5).

For the case of *k* tasks, the Pareto front is bounded in the region defined by 
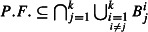
. Again, in the generic case, this means that the Pareto front is usually localized to a restricted region that lies between and includes the archetypes.

## Discussion

This study extends the findings of Shoval et al. that biological systems with trade-offs display variation that falls within a Pareto front shaped as a line, triangle or other polytope. These findings were based on three assumptions – performances have a single global maximum, decay with a norm distance away from their maximum, and that the same norm applies to all performances. Here we explored the effect of relaxing these assumptions. We first relaxed the assumption that norms for all tasks are equal, by analyzing the Pareto fronts obtained for performance functions with non-equal inner-product norms. We find that for two tasks, fronts are hyperbolic, and for three tasks fronts can resemble curved triangles or multi-connected regions with hyperbolic edges. We next relaxed the assumption that performance is maximized at a single point. With performance maximized in a region, we find that the Pareto front selects points on each region closest to the other archetypes, and connects them. Finally, we provide bounds on the Pareto front for the general case of performances not governed by norms, and not necessarily monotonically decreasing. We find that, in the generic case, the front is located between the archetypes, in a region bounded by certain performance contours.

Generally, except in cases where performance contours are very eccentric, relaxing the assumptions of Shoval et al. results in fronts that are mildly curved, instead of straight-edged ones. The fronts have vertices that correspond to the archetypes. This raises the possibility that a wide range of biological situations can be analyzed using the Pareto front approach. One way to use this approach is to analyze whether multi-trait data fall on low-dimensional spaces, and within those spaces on shapes that have pointy vertices. The shared behavior or features of the phenotypes near each vertex can help suggest which task might be optimized by the archetype corresponding to each vertex.

The application of the present findings to biological systems relies on the assumption that natural selection is the main evolutionary force at play, and that there is sufficient genetic variation to reach the optimal phenotypes (Orzack and Sober 2001). Other effects, such as genetic drift due to small population sizes, lack of sufficient genetic variation, lack of time to reach the optimum, physical constraints that preclude certain phenotypes, local fitness maxima that are difficult to escape, can all lead to organisms that do not reach the predicted front.

Of particular interest are developmental constraints – the belief that genetic variations are channeled in particular phenotypic directions by developmental mechanisms. Recent summary of experimental evidence for development constraints (Klingenberg 2010) suggests that such bias is relative, not absolute: in breeding experiments, phenotypes that are different from those found in nature (in the present language – organisms off the Pareto front) can be readily formed. Even absolute developmental constraints, if they exist, do not preclude the present theory, because the developmental mechanism and pathways themselves evolve, and can evolve to ‘encode’ the desired Pareto front (e.g., an allometric curve). This learning is due to the accumulated evolutionary experience of the parental lines of an organism: parental lines experienced a wide range of habitats and as a result evolved developmental mechanisms that can be tuned to optimize phenotypes across that range. Such learning has been demonstrated in computer simulations of evolution in changing environments (Parter et al. 2008; Kashtan et al. 2009).

The theory concerns phenotypes and not genotypes. Extending the analysis to genotypes, using population genetics approaches (Hartl and Clark 1998; Eshel and Feldman 2001; Orr 2005) will be an important step in developing the theory.

It would be interesting to find better bounds on the Pareto front in the case of general performance functions. For example, one may be able to prove under certain assumptions that the front for *k* tasks is a (*k*−1) dimensional surface, and that it deviates from a straight line or polytope by a bounded amount. Advances in methods to fit data to the surfaces predicted in this study could help make precise predictions about the tasks that organisms perform and the fitness weight of each task in the natural environment in which they evolved.

## Supporting Information

**Appendix S1.** The Pareto front is the locus of all points in which the gradients of the performance functions are positive-linearly dependent.

**Appendix S2.** The Pareto front associated with 2 tasks in a 2D-morphospace is a hyperbola.

**Appendix S3.** The Pareto front of 2 tasks in an n-dimensional morphospace has hyperbolic projections.

**Appendix S4.** Calculation of the deviation of the Pareto front from a straight line for 2 tasks in a 2D-morphospace.

**Appendix S5.** Each Pareto front of 2 tasks in a 2D morphospace is generated by a 1-dimensional family of norm pairs.

**Appendix S6.** Generally, for 3 tasks in a 2D morphospace, the norms can be uniquely determined by the shape of the Pareto front.

**Appendix S7.** The boundary of the 3-tasks Pareto front is composed of the three 2-tasks Pareto fronts.

**Appendix S8.** The resulting Pareto front when one of the performance function is maximized in a region.

**Appendix S9.** Bounds on the Pareto front for general performance functions show that normally it is located in a region close to the archetype.

**Appendix S10.** The Pareto front of r strongly concave performance functions is a connected set of Hausdorff dimension of at most r-1.
