# Supplementary material for: The geometry of the Pareto front in biological phenotype space
Source: Ecol Evol. 2013 Apr 17;3(6):1471–83. doi: 10.1002/ece3.528 (PMC3686184; doi:10.1002/ece3.528)
Supplement: Supplementary file 3 [file ece30003-1471-SD3.docx]

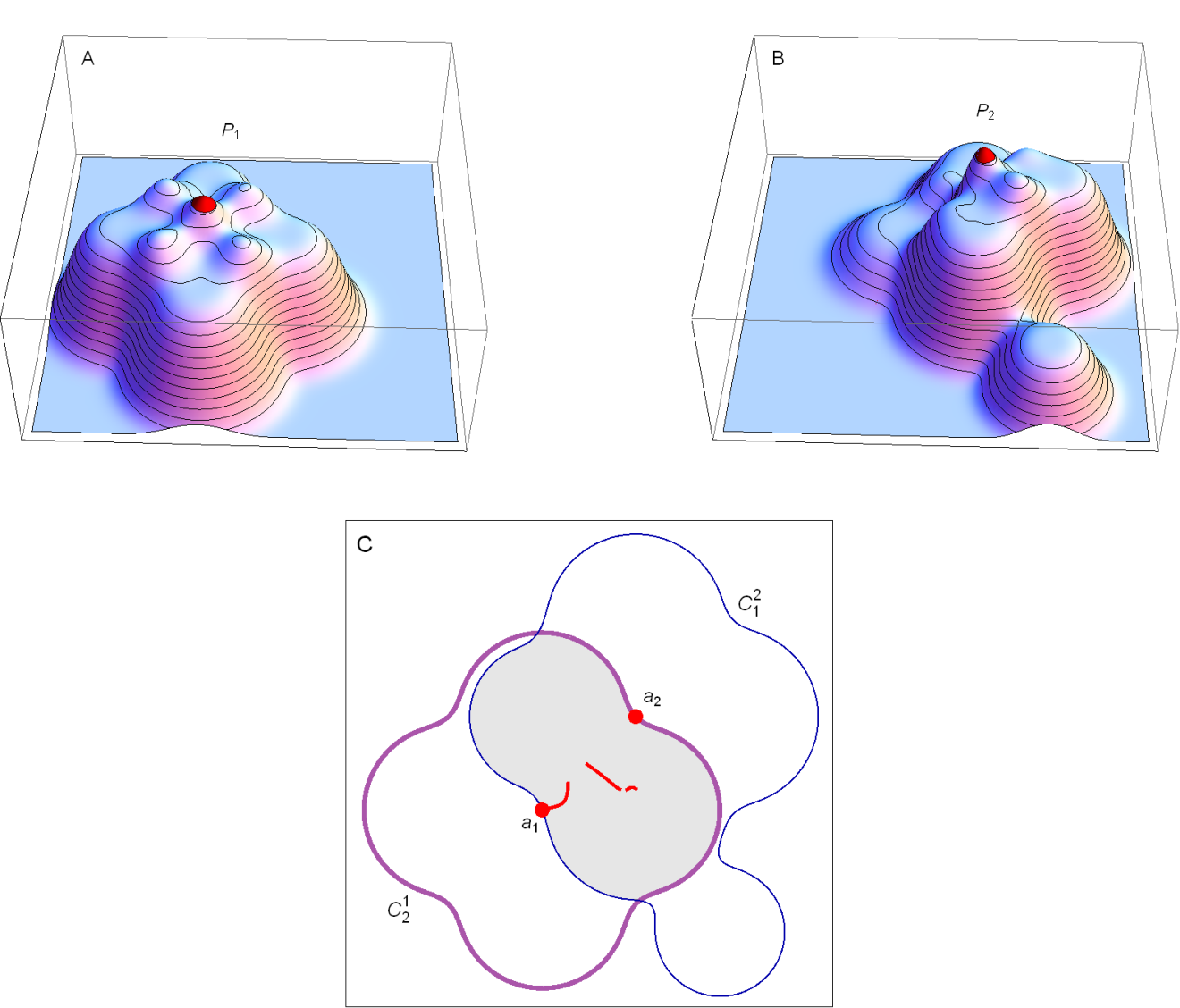


**Figure S5: The Pareto front does not have to be connected for non monotonic performance functions. A and B:** A plot of 2 chosen non-monotonic performance functions, with $\begin{matrix} P_{1}=4.1\exp(-10(x^{2}+y^{2}))+4.9\exp(-2((x-1)^{2}+y^{2})^{2})+5\exp(-2(x^{2}+(y-1)^{2})^{2})+ \\ 5.1\exp(-2(x^{2}+(y+1)^{2})^{2})+5.2\exp(-2((x+1)^{2}+y^{2})^{2}) \end{matrix}$and $\begin{matrix} P_{2}=4.1\exp(-10((x-1)^{2}+(y-1)^{2}))+4.9\exp(-2((x-2)^{2}+(y-1)^{2})^{2})+5\exp(-2((x-1)^{2}+(y-2)^{2})^{2}) \\ +2\exp(-2(x^{2}+(y-1)^{2})^{2})+5.1\exp(-2((x-1)^{2}+y^{2})^{2})+3\exp(-5((x-1.9)^{2}+(y+1.3)^{2})^{2}) \end{matrix}$

**C:** The Pareto front related to those performance functions is not connected. $C_{2}^{1}$ - the contour of performance function 1 going through $a_{2}$, the archetype of task 2, is in thick purple. $C_{1}^{2}$ - the contour of performance function 2 going through $a_{1}$ , the archetype of task 1, is in thin blue. $a_{1}$ and $a_{2}$ are red dots. The Pareto front is plotted in red. It can be seen that it is not connected.
