# Supplementary material for: The geometry of the Pareto front in biological phenotype space
Source: Ecol Evol. 2013 Apr 17;3(6):1471–83. doi: 10.1002/ece3.528 (PMC3686184; doi:10.1002/ece3.528)
Supplement: Supplementary file 4 [file ece30003-1471-SD4.docx]

Supporting Information

Appendix 1 2

The Pareto front is the locus of all points in which the gradients of the performance functions are positive-linearly dependent 2

Appendix 2 10

The Pareto front associated with 2 tasks in a 2D-mirphospace is a hyperbola 10

Appendix 3 16

The Pareto front of 2 tasks in an n-dimensional morphospace has hyperbolic projections 16

Appendix 4 19

Calculation of the deviation of the Pareto front from a straight line for 2 tasks in a 2D-morphospace 19

Appendix 5 22

Each Pareto front of 2 tasks in a 2D morphospace is generated by a 1-dimensional family of norm-pairs 22

Appendix 6 38

Generally, for 3 tasks in a 2D morphospace, the norms can be uniquely determined by the shape of the Pareto front 38

Appendix 7 43

The boundary of the 3-tasks Pareto front is composed of the three 2-tasks Pareto fronts 43

Appendix 8 55

The resulting Pareto front when one of the performance function is maximized in a region 55

Appendix 9 58

Bounds on the Pareto front for general performance functions show that normally it is located in a region close to the archetype 58

Appendix 10 63

The Pareto front of r strongly concave performance functions is a connected set of Hausdorff dimension of at most r-1. 63

References 67

# Appendix 1

## The Pareto front is the locus of all points in which the gradients of the performance functions are positive-linearly dependent

In this appendix we will analytically calculate the Pareto front for a system that needs to perform *r* tasks in an *n*-dimensional morphospace *V*. Each performance function has a single maximum - the archetype $v_{i}^{*}$, and it decreases monotonically with the distance from the archetype, where the distance is derived from a general inner-product norm. Each performance function may depend on a different inner product norm.

$\Rightarrow$ The performance at task *i* is $P_{i}(v)=p_{i}(\left\|v-v_{i}^{*}\right\|_{i}^{2})$, where $\left\|v-v_{i}^{*}\right\|_{i}^{2}=(v-v_{_{i}}^{*})^{T}M_{i}(v-v_{_{i}}^{*})$, $M_{i}$ is a positive definite matrix, and $P_{i}$ is a monotonically decreasing function of a single argument.

We say that *v* is Pareto optimal relative to $P_{1},...,P_{r}$, if for every $v'\neq v$ there exists $j\in\left\{1,...,n\right\}$such that $p_{j}(\left\|v-v_{j}^{*}\right\|_{j}^{2})>p_{j}(\left\|v'-v_{j}^{*}\right\|_{j}^{2})$. Since $P_{1},...,P_{r}$ are monotonically decreasing with their argument, it is equivalent to say that $v$ is Pareto optimal relative to $P_{1},...,P_{r}$ if for every $v'\neq v$ there exists $j\in\left\{1,...,n\right\}$such that $-\left\|v'-v_{j}^{*}\right\|_{j}^{2}<-\left\|v-v_{j}^{*}\right\|_{j}^{2}$. Denote $D_{i}(v)=\left\|v-v_{i}^{*}\right\|_{i}^{2}$. The Pareto front associated with $P_{1},...,P_{r}$ is exactly the same as the Pareto front associated with $D_{1},...,D_{r}$. Hence, from now on, without loss of generality, we will assume that $P_{i}(v)=D_{i}(v)$

Here we will show that the Pareto front associated with *r* tasks in an *n*-dimensional morphospace *V* is given by all points $v$ for which:

(I) $\exists\alpha_{i}\geq0$,$\sum_{i=1}^{r} \alpha_{i}>0$, s.t. $\sum_{i=1}^{r} \alpha_{i}M_{i}(v-v_{i}^{*})=0$.

Note that since $P_{i}(v)=-(v-v_{_{i}}^{*})^{T}M_{i}(v-v_{_{i}}^{*})$, this is equivalent to $\sum_{i=1}^{r} \alpha_{i}\nabla^{\to} P_{i}(v)=0$.

For the rest of this Appendix we will denote $g_{i}:=\nabla^{\to} P_{i}(v)$.

In this Appendix, we will regard V as an inner-product space, using the standard Euclidean inner product (using the standard basis of measured traits). Do not be confused with the inner products associated with each performance function$P_{i}$, which is only used to define $P_{i}$ by measuring distance from the respective archetype $v_{i}^{*}$.

To show that if *v* is Pareto optimal it satisfies property (I), we will rely on a theorem from a paper by Gerstenhaber(4). Our approach is to show that if *v* does *not* satisfy property (I), it is *not* Pareto optimal. Note that if *v* does not satisfy property (I) then $\forall i:g_{i}\neq0$, as if $\exists i:g_{i}=0$, then property (I) will be satisfied by choosing: $\alpha_{i}=1$, $\forall j\neq i:\alpha_{j}=0$.

Here are some relevant definitions quoted from (ref):

The *halfline* generated by the vector $u\in V$ is the set of all points $\{\lambda u|\lambda\geq0\}$.

The *convex polyhedral cone framed by* $u_{1},...,u_{r}\in V$ is the convex hull of their respective halflines.

Note that this is equivalent to the set of all non-negative linear combinations $\{\sum_{i=1}^{r} \lambda_{i}u_{i}|\lambda_{i}\geq0\}$.

Let *A* be a convex polyhedral cone. *L(A)* is defined to be the convex hull of all linear subspaces contained in *A*.

*l(A)* is the dimension of *L(A)*.

A convex polyhedral cone *A* is said to be *pointed* if $l\left( A \right)=0$.

Note that *A* is pointed if and only if *L(A)* does not contain a nontrivial full line (a 1-dimensional linear subspace), if and only if *A* does not contain a full line.

Lemma 1: A convex polyhedral cone *A* framed by $g_{1},...,g_{r}$ is pointed if and only if $g_{1},...,g_{r}$ do not satisfy property (I)

Proof:

1. “Only if”: If *A* is not pointed, it contains a full line spanned by the vector $x\neq0$*.* It means that both *x* and *-x* are in *A.*

$x\in A\Rightarrow x=\sum_{i=1}^{r} \alpha_{i}g_{i}$with $\alpha_{i}\geq0$.

$-x\in A\Rightarrow-x=\sum_{i=1}^{r} \beta_{i}g_{i}$ with $\beta_{i}\geq0$.

$$0=x-x=\sum_{i=1}^{r} \alpha_{i}g_{i}+\sum_{i=1}^{r} \beta_{i}g_{i}=\sum_{i=1}^{r} (\alpha_{i}+\beta_{i})g_{i}$$

$$\alpha_{i}\geq0,\beta_{i}\geq0\Rightarrow\alpha_{i}+\beta_{i}\geq0$$

As$x\neq0$, not all $\alpha_{i}$ are zero, and not all $\beta_{i}$ are zero $\Rightarrow\sum_{i=1}^{r} \alpha_{i}>0$ and $\sum_{i=1}^{r} \beta_{i}>0$ $\Rightarrow\sum_{i=1}^{r} (\alpha_{i}+\beta_{i})\Rightarrow\sum_{i=1}^{r} \alpha_{i}+\sum_{i=1}^{r} \beta_{i}>0$.

We showed that $g_{1},...,g_{r}$ satisfy property (I).

Hence, if $g_{1},...,g_{r}$ do not satisfy property (I), *A* is pointed

2. “If”: If $g_{1},...,g_{r}$ satisfy property (I), there exist $\alpha_{1},...,\alpha_{r}\geq0,\sum_{i=1}^{r} \alpha_{i}>0$ such that $\sum_{i=1}^{r} \alpha_{i}g_{i}=0$. Choose *i* such that $\alpha_{i}\neq0$*.* Denote $x=\alpha_{i}g_{i}$. Then$x\neq0$. $x\in A$ by definition.

$\sum_{\begin{matrix} j=1 \\ j\neq i \end{matrix}}^{r} \alpha_{j}g_{j}=-\alpha_{i}g_{i}=-x$.

$\sum_{\begin{matrix} j=1 \\ j\neq i \end{matrix}}^{r} \alpha_{j}g_{j}\in A$ by definition $\Rightarrow-x\in A$*.*

$x\neq0,x\in A$ and $-x\in A$ A contains the non-trivial full line:  A is not pointed.

Hence, if *A* is pointed, $g_{1},...,g_{r}$do not satisfy property (I)

According to theorem 17 in (ref), a convex polyhedral cone *A* is pointed if and only if there exists a half plane $H\subset V$such that, except for the origin, *A* is contained in the interior of *H.*

The interior of a half plane is defined as $\{x|h\cdot x>0\}$, where *h* is the unit vector perpendicular to the hyperplane separating the space into 2 halves.

Note that for a convex polyhedral cone $A$ framed by nonzero vectors$g_{1},...,g_{r}$, and for every vector *h*:

$(\forall0\neq x\in A:x\cdot h>0)\Leftrightarrow\forall i:g_{i}\cdot h>0$.

“” is trivial, as $g_{i}\in A$

“” stems from the fact that $\forall0\neq x\in A,\exists(\alpha_{i}\geq0,\sum_{i=1}^{r} \alpha_{i}>0)$*such that* $x=\sum_{i=1}^{r} \alpha_{i}g_{i}$

Putting together all of the above, we get:

$g_{1},...,g_{r}$do not satisfy property (I) $\Leftrightarrow$

The convex polyhedral cone *A* framed by $g_{1},...,g_{r}$ is pointed $\Leftrightarrow$

There exists a half plane $H\subset V$such that, except for the origin, *A* is contained in the interior of *H* $\Leftrightarrow$

There exists a vector *h* such that $\forall i:h\cdot g_{i}>0$ (note that $\forall i:g_{i}\neq0$)

Claim 1: If there exists a vector $h$, such that $\forall i:h\cdot g_{i}(v)>0$, then *v* is not Pareto optimal.

Proof: Consider a vector $v'=v+\varepsilon h$. The performance of task *i* of *v’* is

$P_{i}(v')=P_{i}(v+\varepsilon h)$. Since $P_{i}$ is smooth, we can approximate:

$$P_{i}(v+\varepsilon h)=P_{i}(v)+\varepsilon h\cdot\nabla^{\to} P_{i}(v)+O(\varepsilon^{2})=P_{i}(v)+\varepsilon h\cdot g_{i}+O(\varepsilon^{2})$$

By assumption, $h\cdot g_{i}>0$so for small enough $\varepsilon$,$\forall i:P_{i}(v')>P_{i}(v)$.

This implies that *v* is not Pareto optimal, as required.

From all of the above we can deduce that if $g_{1}(v),...,g_{r}(v)$ do not satisfy property (I), *v* is not Pareto optimal.

Note that we’ve shown that a Pareto optimal point must satisfy property (I), without using any prior assumptions on the nature of the performance functions $P_{i}(v)$, beside differentiability.

On the other hand, if a point $v$ satisfies property (I), it is Pareto optimal:

Consider the following function ($\alpha_{i}$ are given by property (I)):

$$f(u)=\sum_{i=1}^{r} \alpha_{i}P_{i}(u)=-\sum_{i=1}^{r} \alpha_{i}(u-v_{i}^{*})^{T}M_{i}(u-v_{i}^{*})$$

*v* is an extreme point of *f* as $(\nabla^{\to} f)(v)=\sum_{i=1}^{r} \alpha_{i}\nabla^{\to} P_{i}(v)=\sum_{i=1}^{r} \alpha_{i}g_{i}(v)=0$.

*f* has a single maximal point, since:

$$\nabla^{\to} \sum_{i=1}^{r} \alpha_{i}\nabla^{\to} P_{i}(v)=-\nabla^{\to} \sum_{i=1}^{r} \alpha_{i}(u-v_{i}^{*})^{T}M_{i}(u-v_{i}^{*})=-2\sum_{i=1}^{r} \alpha_{i}M_{i}(u-v_{i}^{*})=0$$

implies

$$v_{\max}=(\sum_{i=1}^{r} \alpha_{i}M_{i})^{-1}(\alpha_{i}M_{i}v_{i}^{*})$$

($\sum_{i} \alpha_{i}M_{i}$ is positive-definite as all $M_{i}$ are positive definite, all $\alpha_{i}$ are non-negative and not all of them are zero, which implies $\sum_{i} \alpha_{i}M_{i}$ is also invertible). And -

$$\nabla^{2}\sum_{i=1}^{r} \alpha_{i}P_{i}(v)=-\sum_{i=1}^{r} \alpha_{i}M_{i}$$

which is the negative of a positive definite matrix, and hence a negative definite matrix $\Rightarrow v_{\max}$ is a maximum. Also notice that $v_{\max}$ is a global maximum since *f* is defined continuously on the entire space and has a single extremum.

So if *v* satisfies property (I) it maximizes $\sum_{i=1}^{r} \alpha_{i}P_{i}$ (i.e. $v=v_{\max}$). If it is not Pareto optimal, it means that there is a point $v'\neq v$ such that $\forall i:P_{i}(v)\leq P_{i}(v')$and since $\forall i:\alpha_{i}\geq0$ we get $\sum_{i=1}^{r} \alpha_{i}P_{i}(v)<\sum_{i=1}^{r} \alpha_{i}P_{i}(v')$, in opposed to the maximality of *v*. So if *v* satisfies property (I), it is Pareto optimal.

Conclusion 1: $v$ is Pareto optimal $\Leftrightarrow v$satisfies property (I) $\Leftrightarrow$ there exists no vector *h* such that $\forall i:h\cdot g_{i}>0$.

Conclusion 2: *v* is Pareto optimal $\Leftrightarrow v$ satisfies property (I) $\Leftrightarrow v$maximizes $\sum_{i=1}^{r} \alpha_{i}P_{i}(u)$ (with $\alpha_{i}$ given by property (I)).

In other words, the set of Pareto optimal points equals the set of all points $v$ for which there exist $\alpha_{i}\geq0,\sum_{i=1}^{r} \alpha_{i}>0$, such that $\sum_{i=1}^{r} \alpha_{i}\nabla^{\to} P_{i}(v)=0$

Note that as $\sum_{i=1}^{r} \alpha_{i}\neq0$, we can define $\beta_{i}=\frac{\alpha_{i}}{\sum_{i=1}^{r} \alpha_{i}}$. Then $\beta_{i}\geq0, \sum_{i=1}^{r} \beta_{i}=1$, and:

$\sum_{i=1}^{r} \alpha_{i}\nabla P_{i}\left( v \right)=0\Leftrightarrow\frac{1}{\sum_{i=1}^{r} \alpha_{i}}\sum_{i=1}^{r} \alpha_{i}\nabla P_{i}\left( v \right)=0\Leftrightarrow\sum_{i=1}^{r} \beta_{i}\nabla P_{i}\left( v \right)=0$

Hence, $\exists\alpha_{i}\geq0, \sum_{i=1}^{r} \alpha_{i}>0$ s.t. $\sum_{i=1}^{r} \alpha_{i}\nabla P_{i}(v)=0$

$\Leftrightarrow\exists\alpha_{i}\geq0, \sum_{i=1}^{r} \alpha_{i}=1$, s.t. $\sum_{i=1}^{r} \alpha_{i}\nabla P_{i}(v)=0$

Thus, the set of Pareto optimal points equals the set of all points $v$ for which there exist $\alpha_{i}\geq0, \sum_{i=1}^{r} \alpha_{i}=1$, such that $\sum_{i=1}^{r} \alpha_{i}\nabla P_{i}(v)=0$

We consider performance functions of the form $P_{i}\left( v \right)=\left( v-v_{i}^{*} \right)^{T}M_{i}\left( v-v_{i}^{*} \right)\Rightarrow\nabla P_{i}\left( v \right)=M_{i}(v-v_{i}^{*})$. Hence,

$$\sum_{i=1}^{r} \alpha_{i}\nabla P_{i}(v)=0\Rightarrow{\sum_{i=1}^{r} \alpha_{i}M}_{i}\left( v-v_{i}^{*} \right)= 0\Rightarrow v=({\sum_{i} \alpha_{i}M_{i})}^{-1}(\alpha_{i}M_{i}v_{i}^{*})$$

Conclusion 3: The set of Pareto optimal points, associated with $P_{1},\ldots,P_{r}$ is given by:

$$\left\{ \left( \sum_{i} \alpha_{i}M_{i} \right)^{-1}\sum_{i} \left( \alpha_{i}M_{i}v_{i}^{*} \right) | \alpha_{i}\geq0, \sum_{i=1}^{3} \alpha_{i}=1 \right\}$$

# Appendix 2

## The Pareto front associated with 2 tasks in a 2D-mirphospace is a hyperbola

We would like to prove that the Pareto front associated with 2 tasks in a 2-dimensional morphospace is a section of a hyperbola or a line. As explained in Appendix 1, the performance of task $i$ is taken to be:

$${P_{i}= -\left( v-v_{i}^{*} \right)}^{T}M_{i}\left( v-v_{i}^{*} \right)$$

Where $M_{i}$ is a positive-definite 2$\times2$ matrix, and $v_{i}^{*}$ = ($x_{i}^{*},y_{i}^{*})$ is the archetype for task $i$*.* Positive definite matrices have positive eigenvalues and are Hermitian, and thus $M_{i}$ can be diagonalized by a rotation matrix. Thus $M_{i}=R(\theta_{i})\left( \begin{matrix} \lambda_{i,1}^{2} & 0 \\ 0 & \lambda_{i,2}^{2} \end{matrix} \right)R(-\theta_{i})$ , where $R(\theta)$ is an orthogonal matrix (rotation matrix by angle $\theta$) and $\lambda_{i,1}>0, \lambda_{i,2}>0$ (i.e. real and non-zero).

Note: The contours of such performance functions $P_{i}$ are concentric ellipses with eccentricity $\lambda=\frac{\lambda_{i,2}}{\lambda_{i,2}}$ which are rotated by an angle of $\theta$ relative to the y axis. These contours and their parameters are widely used in the main text.

As will be explained immediately, we can assume without loss of generality that:

1. $M_{1}=I$, implying - $\left( v-v_{1}^{*} \right)^{T}M_{1}\left( v-v_{1}^{*} \right)= \left( v-v_{1}^{*} \right)^{T}\left( v-v_{1}^{*} \right)$.

$v_{1}^{*}=(0,0)$

$v_{2}^{*}=(1,0)$

2. One of $M_{2}'s$ eigenvalues is 1.

Those assumptions will be true in a rotated, translated and rescaled coordinate system.

We will show that the Pareto front is a hyperbola. Since a hyperbola remains a hyperbola under such transformations, and all such transformations are invertible, it is enough to work in the coordinate system where the above assumptions hold.

The first and second assumptions are satisfied by the transformation $v\to Tr(v_{1}^{*})T_{1}v$ where:

$$T_{1}=R(\theta_{1})\left( \begin{matrix} \frac{1}{\lambda_{i,1}} & 0 \\ 0 & \frac{1}{\lambda_{i,2}} \end{matrix} \right)$$

(then translate such that $v_{1}^{*}$ is at $(0,0)$, rotate such that $M_{1}$ is diagonal, and then scale such that $M_{1}=I$).

Under the above transformation, the second archetype moves to:

$$\left\{ \begin{matrix} \tilde{\Delta x} & \tilde{\Delta y} \end{matrix} \right\}=T_{1}^{-1}(v_{2}^{*}-v_{1}^{*})$$

The third assumption is satisfied, while keeping assumptions 2, and keeping $M_{1}$ scalar by further applying:

$T_{2}= \left( \begin{matrix} \tilde{\Delta x} & -\tilde{\Delta y} \\ \tilde{\Delta y} & \tilde{\Delta x} \end{matrix} \right)$

(Move the second archetype to the $x$ axis and then scale it to be at $(1,0)$).

This transformation is invertible since $\tilde{\Delta x}\neq0$ or $\tilde{\Delta y}\neq0$

We apply the transformation $v\to T_{2}T_{1}v$.

In this coordinate system:

The functional $\left. \left\| v-v_{1}^{*} \right\| \right._{1}^{2}$ becomes $\left\| T_{2}T_{1}v-v_{1}^{*} \right\|_{1}^{2}={\left( \tilde{\Delta x}^{2}+\tilde{\Delta y}^{2} \right)v}^{T}v$

The functional $\left. \left\| v-v_{2}^{*} \right\| \right._{2}^{2}$ becomes $\left\| T_{2}T_{1}v-v_{2}^{*} \right\|_{2}^{2}= \left( v-\left( 1,0 \right) \right)^{T}M\left( v- (1,0) \right)$

Where $M= \left( T_{2}T_{1} \right)^{T}M_{2} T_{2}T_{1}$

Since $M_{2}$ is positive definite and $T_{2}T_{1}$ is invertible, $M$ is positive definite.

$\to M=R(\theta)\left( \begin{matrix} \lambda_{1}^{2} & 0 \\ 0 & \lambda_{2}^{2} \end{matrix} \right)R(-\theta)$, with $\lambda_{1}>0,\lambda_{2}>0$

Finally, assumption 1 and 4 are reached by using the following lemma:

Lemma 1: The Pareto front is invariant to scaling of any of the norms

Proof: $v$ is Pareto optimal with respect to $P_{1}(v),P_{2}(v)$ if for every $v^{'}\neq v$ there exists $j\in\left\{ 1,2 \right\}$ such that $\left\| v-v_{j}^{*} \right\|_{j}^{2} < \left\| v^{'}-v_{j}^{*} \right\|_{j}^{2}$. Let $\beta_{j}^{2}>0$ be a constant for each $j\in\left\{ 1,2 \right\}$.

$\left\| v-v_{j}^{*} \right\|_{j}^{2} < \left\| v^{'}-v_{j}^{*} \right\|_{j}^{2}\Leftrightarrow\beta_{j}^{2}\left\| v-v_{j}^{*} \right\|_{j}^{2} < \beta_{j}^{2}\left\| v^{'}-v_{j}^{*} \right\|_{j}^{2}$, which means that the norm-pairs $\{\beta_{j}^{2}\left\| v-v_{j}^{*} \right\|_{j}^{2}$} and $\{\left\| v-v_{j}^{*} \right\|_{j}^{2}\}$ result in the same Pareto front.

We choose $\beta_{1}^{2}=\frac{1}{\left( \tilde{\Delta x}^{2}+\tilde{\Delta y}^{2} \right)}$ and $\beta_{2}^{2}=\frac{1}{\lambda_{1}^{2}}$ and we get that

${\left( \tilde{\Delta x}^{2}+\tilde{\Delta y}^{2} \right)v}^{T}v ,\left( v-\left( 1,0 \right) \right)^{T}R\left( \theta\right)\left( \begin{matrix} \lambda_{1}^{2} & 0 \\ 0 & \lambda_{2}^{2} \end{matrix} \right)R\left( -\theta\right)\left( v-\left( 1,0 \right) \right)$ results in the same Pareto front as

$v^{T}v ,\left( v-\left( 1,0 \right) \right)^{T}R\left( \theta\right)\left( \begin{matrix} 1 & 0 \\ 0 & \frac{\lambda_{2}^{2}}{\lambda_{1}^{2}} \end{matrix} \right)R\left( -\theta\right)\left( v-\left( 1,0 \right) \right)$. Denote $\lambda^{2}= \frac{\lambda_{2}^{2}}{\lambda_{1}^{2}}$.

To conclude, we can assume without loss of generality that:

$${{||v-v_{i}^{*}||}_{i}}^{2}=\left( v-v_{i}^{*} \right)^{T}M_{i}\left( v-v_{i}^{*} \right){=\left( v-v_{i}^{*} \right)}^{T}R\left( \theta\right)\left( \begin{matrix} 1 & 0 \\ 0 & \lambda_{i}^{2} \end{matrix} \right)R\left( -\theta\right)\left( v-v_{i}^{*} \right)$$

With $v_{1}^{*}=\left( 0,0 \right), v_{2}^{*}=\left( 1,0 \right), \lambda_{1}^{2} =1, \lambda_{2}^{2} =\lambda^{2}$

As seen in Appendix 1 (conclusion 1), the Pareto front associated with 2 tasks is given by:

$$\left\{ v \right| \exists0\leq\alpha\leq1 s.t. \alpha g_{1}(v)+\left( 1-\alpha\right)g_{2}\left( v \right)= 0\}$$

Where $g_{i}={\nabla P}_{i}\left( v \right)$

Thus, the gradients of the 2 performance functions at a point that is Pareto optimal relative to 2 tasks point in opposite directions.

Let’s try to give a geometrical intuition to the above statement. The gradients of the performance functions at point $v$ point in opposite directions if and only if $v$ is a tangency point between 2 contours of the performance function.

Each point $v$ is on some contour of $P_{1}$: $C_{1}= \left\{ v^{'} \right|{{||v^{'}-v_{1}^{*}||}_{1}}^{2}={{||v-v_{1}^{*}||}_{1}}^{2}\}$, and on a contour of $P_{2}$: $C_{2}=$ $\left\{ v^{'} \right|{{||v^{'}-v_{2}^{*}||}_{2}}^{2}={{||v-v_{2}^{*}||}_{2}}^{2}\}$. As mentioned in the main text, $C_{1}$ and $C_{2}$ are ellipses. Point $v$ is a common point to $C_{1}$ and $C_{2}$. It can be an intersection point, an internal tangency point (when the intersection of the interiors of both ellipses is non-empty), or an external tangency point.

The gradients of the performance functions at point $v$ point in opposite directions $\Leftrightarrow v$ is an external tangency point between the 2 contours.

If the gradients of the performance function at a point $v$ do not point in opposite directions, it is either an internal tangency point or an intersection point between $C_{1}$ and $C_{2}$. In both cases ${C_{1}}^{○}\cap{C_{2}}^{○}\neq\phi$, where ${C_{i}}^{○}=\left\{ v^{'} \right|{{||v^{'}-v_{i}^{*}||}_{i}}^{2}<{{||v-v_{i}^{*}||}_{i}}^{2}\}$ - all points that outperform $v$ in the $i$-th task (see figure S1a). It means that there exists $v^{'}∊ {C_{1}}^{○}\cap{C_{2}}^{○}\Rightarrow P_{1}\left( v^{'} \right)>P_{1}\left( v \right)$ and $P_{2}\left( v^{'} \right)>P_{2}\left( v \right)$, i.e., if the gradients at point $v$ don’t point at opposite directions, we can find a point $v^{'}$ at the neighborhood of $v$ that performs both tasks better than it. That is why a point for which the gradients don’t point in opposite directions is not Pareto optimal. In case of an external tangency point $v$, ${C_{1}}^{○}\cap{C_{2}}^{○}=\{v\}$, in which case there are no points that outperform $v$ in both tasks (figure S1b).


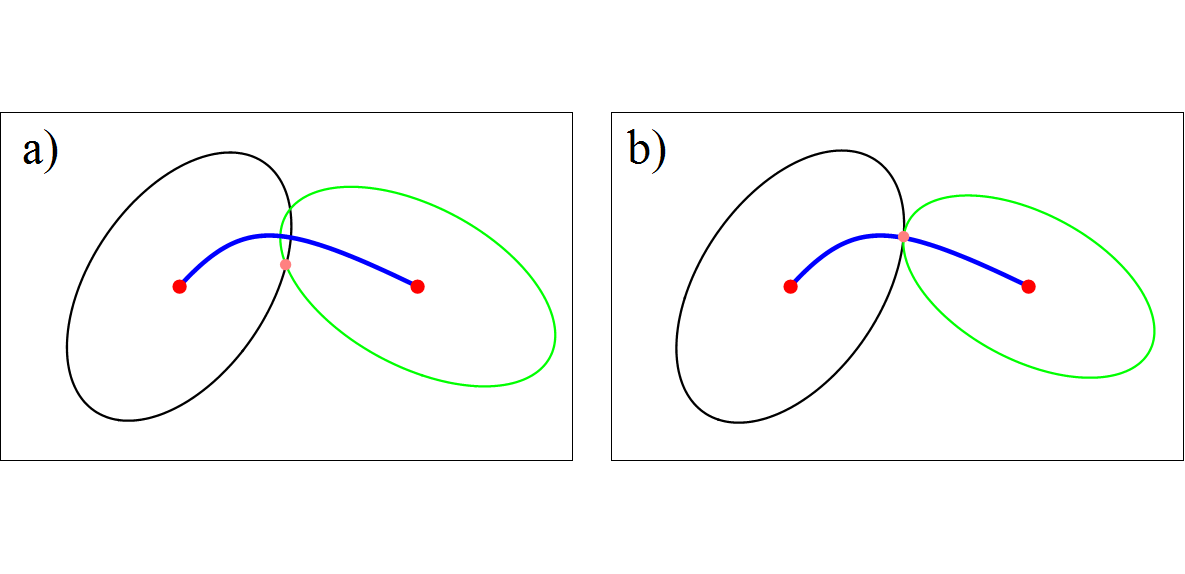


Figure S1: The Pareto front is composed of tangency points between performance functions’ contours. Archetypes are marked as red dots, Pareto front in blue. (a) A point whose contours intersect is *not* Pareto optimal; points in the intersection area outperform it in both tasks. (b) A point whose contours are tangent is optimal – there is no intersection area with other outperforming points.

Denote the Pareto front by *P.F*., as seen in Appendix 1 (conclusion 3):

$$P.F=\{({\alpha M_{1}+\left( 1-\alpha\right)M_{2})}^{-1}\left( \alpha M_{1}v_{1}^{*}+\left( 1-\alpha\right)M_{2}v_{2}^{*} \right) | 0\leq\alpha\leq1\}$$

$$\Rightarrow\forall0\leq\alpha\leq1 \exists v\in P.F :$$

$${v= \left( \alpha M_{1}+ \left( 1-\alpha\right)M_{2} \right)}^{-1}\left( \alpha M_{1}v_{1}^{*}+ \left( 1-\alpha\right)M_{2}v_{2}^{*} \right)=$$

$$\left( R\left( \theta\right)\left( \alpha I+\left( 1-\alpha\right)\left( \begin{matrix} 1 & 0 \\ 0 & \lambda^{2} \end{matrix} \right) \right)R\left( -\theta\right) \right)^{-1}\left( \left( 1-\alpha\right) R\left( \theta\right)\left( \begin{matrix} 1 & 0 \\ 0 & \lambda^{2} \end{matrix} \right)R\left( {-\theta}^{'} \right)\left( 1,0 \right) \right)=$$

$$(-\frac{\left( -1+\alpha\right)\left( -\alpha+\left( -2+\alpha\right)\lambda^{2}+\alpha\left( -1+\lambda^{2} \right)\mathrm{Cos}\left[ 2\theta\right] \right)}{-2\alpha+2\left( -1+\alpha\right)\lambda^{2}},\frac{\left( -1+\alpha\right)\alpha\left( -1+\lambda^{2} \right)\mathrm{Cos}\left[ \theta\right]\mathrm{Sin}\left[ \theta\right]}{\alpha+\lambda^{2}-\alpha\lambda^{2}})$$

When $\alpha\to0$, $v\to\left( 1, 0 \right).$ When $\alpha\to1$, $v\to\left( 0,0 \right)$. Thus, the Pareto front lies on a curve between the archetypes ${v_{1}^{*}}^{'}$ and ${v_{2}^{*}}^{'}$.

We next characterize this curve. Denote $v=(x, y)$. By eliminating $\alpha$, we get that our curve is a quadratic curve that satisfies:

$$x^{2}\left( 1-\lambda^{2} \right)Sin^{2}\left( 2 \theta\right)-2xy\left( 1-\lambda^{2} \right)Sin\left( 2 \theta\right)Cos\left( 2 \theta\right)- y^{2}\left( 1-\lambda^{2} \right)Sin^{2}\left( 2 \theta\right)-x \left( 1-\lambda^{2} \right)Sin^{2}\left( 2 \theta\right)+y ((1+\lambda^{2}-\left( 1-\lambda^{2} \right)Cos\left( 2 \theta\right)) Sin\left( 2 \theta\right)=0$$

A general quadratic curve is of the form $A_{xx}x^{2}+2A_{xy}xy+A_{yy}y^{2}+2A_{x}x+2A_{y}y+A=0$

If $J= A_{xx}A_{yy}$ - $A_{xy}^{2}<0$ and $\Delta=Det\left( \begin{matrix} A_{xx} & A_{xy} & A_{x} \\ A_{xy} & A_{yy} & A_{y} \\ A_{x} & A_{y} & A \end{matrix} \right) \neq0$ then the equation represents a hyperbola.

For our curve:

$$J=A_{xx}A_{yy}, A_{xy}^{2}= -\left( -1+\lambda^{2} \right)^{2}\sin^{2} \left( 2\theta\right), \Delta=4\lambda^{2}\left( -1+\lambda^{2} \right){\sin([2\theta]}^{4})$$

$J<0 and \Delta\neq0 unless$ $\lambda=1 or \theta=0 or \frac{\pi}{2}$ and then $J=0$ and $\Delta=0$. This means that the Pareto optimal points lie on a section of a hyperbola between the 2 archetypes, unless $\lambda=1 or \theta\in\{0,\frac{\pi}{2}\}$ and then they lie on the line between the archetypes. This matches previous results [1] since if $\lambda=1$ both norms are equal. This also shows that if both norms’ contours are perpendicular to the line between them ($\theta_{i}\in\left\{ 0,\frac{\pi}{2} \right\}$), the Pareto front is the line between the archetypes.

To conclude, we showed that the Pareto front associated with 2 archetypes in a 2-D trait space with performance functions that decrease monotonically with a general inner-product norm distance from the archetype is a section of a hyperbola (or a line) between the archetypes.

# Appendix 3

## The Pareto front of 2 tasks in an n-dimensional morphospace has hyperbolic projections

In this Appendix we will characterize the Pareto front for 2 tasks in an n-dimensional morphospace.

We would like to prove that the Pareto front in an n-dimensional trait space for a system that needs to perform 2 tasks, when each performance function depends on a different inner-product norm, is a 1-dimensional curve connecting the archetypes whose projections on the principle planes of a certain coordinate system are hyperbolae (or lines).

As shown in Appendix 1, the performance functions $P_{i}$ can be written as

$$P_{i}\left( v \right)=-\left( v-v_{i}^{*} \right)^{T}M_{i}\left( v-v_{i}^{*} \right)$$

with positive-definite $M_{i}$ that can be decomposed as

$$M_{i}=R_{i}\left( \begin{matrix} \lambda_{i,1}^{2} & 0 & 0 \\ 0 & \ldots& 0 \\ 0 & 0 & \lambda_{i,N}^{2} \end{matrix} \right)R_{i}^{T}$$

where $R_{i}$ is an orthogonal matrix. The Pareto front is the locus of all points satisfying

$$\alpha_{1} M_{1}\left( v-v_{1}^{*} \right)+\alpha_{2}M_{2}\left( v-v_{2}^{*} \right)= 0, \alpha_{1}+\alpha_{2}=1$$

Moreover, there exists a basis in which $M_{1}=I$, simply redefine:

$$v :={\left( \begin{matrix} \lambda_{i,1} & 0 & 0 \\ 0 & \ldots& 0 \\ 0 & 0 & \lambda_{i,n} \end{matrix} \right)R}_{n}^{T}v$$

In which $M_{2}$ (describing $P_{2}$) is still positive-definite (as it does not depend on the basis).

Denote $A_{i}= \alpha_{i}M_{i}$, $A= A_{1}+ A_{2}$, $B_{i}=A^{-1}A_{i}$

$B_{1}+ B_{2}= A^{-1}(A_{1}+ A_{2}$) = $A^{-1}A=I$

$v$ =$A^{-1}$($A_{1}v_{1}^{*}+ A_{2}v_{2}^{*}$) = $B_{1}v_{1}^{*}+B_{2}v_{2}^{*}$

The eigenvalues of $M_{i}$ are positive. The eigenvalues of $A_{i}$ are non-negative as $\alpha_{i}\geq0$. As $\alpha_{i}$ are real, $A_{i}$ are symmetric. $A_{1}$ has a single eigenvalue - $\alpha_{1}$, since $M_{1}$ is assumed to be the identity matrix. The eigenvalues of $A_{2}$ are $\alpha_{2}\lambda_{j}^{2}$. As $A_{1}$ is scalar, the eigenvectors of $A= A_{1}+ A_{2}$ are the same as those of $A_{2}$, with eigenvalues $\alpha_{1} + \alpha_{2} \lambda_{j}^{2}.$ These eigenvalues are positive since at least one $\alpha_{i}$ is positive, therefore A is invertible. The eigenvalues of $A^{-1}$ are $\frac{1}{\alpha_{1} + \alpha_{2} \lambda_{j}^{2}}$. $A$ is symmetric as a sum of 2 symmetric matrices.

As $A_{1}$ is scalar, it commutes with $A_{2}$. $\left[ A_{1}, A_{2} \right]=A_{1}A_{2}-A_{2}A_{1}=0\Rightarrow\left[ A, A_{1} \right]=0$ and $\left[ A, A_{2} \right]=0\Rightarrow\left[ A^{-1}, A_{1} \right]=0$ and $\left[ A^{-1}, A_{2} \right]=0\Rightarrow B_{i}^{T}=\left( A^{-1}A_{i} \right)^{T}=A_{i}^{T} {A^{-1}}^{T}= A_{i}{A^{T}}^{-1}= A_{i}A^{-1}= B_{i}$.

Also, $A^{-1}$ has the same eigenvectors as $A, A_{1}$ and $A_{2}$ so the eigenvalues of $B_{1}$ are $\frac{\alpha_{1}}{\alpha_{1}+\alpha_{2} \lambda_{j}^{2}}= \frac{\alpha}{\alpha+(1-\alpha) \lambda_{j}^{2}}$ and of $B_{2}$ are $\frac{1-\alpha}{1-\alpha+\frac{\alpha}{\lambda_{j}^{2}}}$

$B_{1}$ and $B_{2}$ are mutually diagonalizable$:$ Let D be such that $DB_{1}D^{-1}=\Lambda_{1}$. Now $B_{2}=I- B_{1}\Rightarrow DB_{2}D^{-1}= D\left( I- B_{1} \right)D^{-1}=I-\Lambda_{1}$

$v$ = $D^{-1}\Lambda_{1}Dv_{1}^{*}+D^{-1}\left( I-\Lambda_{1} \right)D v_{2}^{*}$ = $v_{2}^{*}+ D^{-1}\Lambda_{1}D \left( v_{1}^{*}-v_{2}^{*} \right)\to D\left( v-v_{2}^{*} \right)= \Lambda_{1}D\left( v_{1}^{*}-v_{2}^{*} \right)$

Consider the rotated coordinate system, $v:=D^{-1}v$, and rename $v_{2}^{*} :=D^{-1}v_{2}^{*}$ . In this system $v- v_{2}^{*}= \Lambda_{1}\left( v- v_{1}^{*} \right)=\left( \begin{matrix} \frac{\alpha}{\alpha+(1-\alpha) \lambda_{1}^{2}} & \ldots& 0 \\ \vdots& \ddots& \vdots\\ 0 & \ldots& \frac{\alpha}{\alpha+(1-\alpha) \lambda_{n}^{2}} \end{matrix} \right)\left( v- v_{1}^{*} \right)$.

Denote $v=\left( v_{1},\ldots,v_{n} \right), v_{1}^{*}=\left( v_{1,1}^{*},\ldots,v_{1,n}^{*} \right), v_{2}^{*}=\left( v_{2,1}^{*},\ldots,v_{2,n}^{*} \right)$

For every 2 coordinates $j, k$ we get that:

$$v_{k}v_{j}\left( \lambda_{j}-\lambda_{k} \right)+v_{k}\left( v_{1,j}\lambda_{k}-v_{2,j}\lambda_{j} \right)+v_{j}\left( v_{2,k}\lambda_{k}-v_{1,k}\lambda_{j} \right)+v_{1,k}v_{2,j}\lambda_{j}- v_{1,j}v_{2,k}\lambda_{k}=0$$

Thus, the projection of the Pareto front on any main plane is a quadratic curve, with parameters:

$$J=A_{xx}A_{yy}-A_{xy}^{2}=-\left( \lambda_{j}-\lambda_{k} \right)^{2}, \Delta= -\left( \lambda_{j}-\lambda_{k} \right)^{2}$$

For components for which $\lambda_{j}\neq\lambda_{k}$, $J<0$ and $\Delta\neq0$, so the projection on their plane is a hyperbola. For components for which $\lambda_{j}=\lambda_{k}$, $J=0$ and $\Delta=0$, so the projection on their plane is a line.

To conclude, we get that the Pareto front associated with 2 archetypes in an n-dimensional trait space is a 1-dimensional curve between the 2 archetypes. There exists a coordinate system such that the projection of this curve on each principal plane is a section of a hyperbola or a line between the projections of the archetypes.

# Appendix 4

## Calculation of the deviation of the Pareto front from a straight line for 2 tasks in a 2D-morphospace

In this Appendix, we calculate the maximal deviation of the Pareto front associated with 2 tasks in a 2D morphospace from the line between the archetypes (which is the Pareto front in case the norms are equal).

The deviation of the front from the line between the archetypes is defined as the maximal height $h$ of a point on the front with respect to the line*,* divided by the Euclidean distance between the archetypes, *D.*

We can assume without loss of generality that the archetypes are at $(-\frac{1}{2}, 0)$ and $(\frac{1}{2}, 0)$, since this assumption can be satisfied by a combination of translations/rotations and *isometric* scaling, all of which preserve distance ratios. Notice that in this case $D=1$ so the ratio $\frac{h}{D}$ is simply $h$.

Norm $i$ depends on the parameters $\lambda_{i},\theta_{i}$, i.e - $M_{i}= R\left( \theta_{i} \right)\left( \begin{matrix} 1 & 0 \\ 0 & \lambda_{i}^{2} \end{matrix} \right)R\left( -\theta_{i} \right)$. During the solution we assume that $0\leq\theta_{i}<\frac{\pi}{2}$. This is possible since the Pareto front is symmetric under the transformation: $\theta_{i},\lambda_{i}\to\theta_{i}+\frac{\pi}{2},\frac{1}{\lambda_{i}}$ (see Appendix 5)

Appendix 1 (Conclusion 3) gives a parametric representation of the front, $\{x\left( u \right), y\left( u \right)\}$, with a parameter $0\leq u\leq1$. As the line between the archetypes lies on the $x$ axis, the maximal deviation is given by $\max|y(u)|$. $\max|y(u)|= |y\left( u_{max} \right)|$, for $u_{max}$ with $\partial_{u}y|_{u_{max}}=0$ (The maximum is not obtained at the edges, since $y\left( 0 \right)=y\left( 1 \right)=0$).

So, from Appendix 1:

$$y\left( u \right)= -\frac{\left( -1+u \right)u\left( -\left( -1+\lambda_{1}^{2} \right)\mathrm{Sin}\left[ 2\theta_{1} \right]\left( {\mathrm{Cos}\left[ \theta_{2} \right]}^{2}+\lambda_{2}^{2}{\mathrm{Sin}\left[ \theta_{2} \right]}^{2} \right)-\frac{1}{2}\left( -1+\lambda_{2}^{2} \right)\left( -1-\lambda_{1}^{2}+\left( -1+\lambda_{1}^{2} \right)\mathrm{Cos}\left[ 2\theta_{1} \right] \right)\mathrm{Sin}\left[ 2\theta_{2} \right] \right)}{2\lambda_{2}^{2}+u\left( 1+\lambda_{1}^{2}+\left( -3+\lambda_{1}^{2} \right)\lambda_{2}^{2}+u\left( -1+\lambda_{1}^{2}+\lambda_{2}^{2}-\lambda_{1}^{2}\lambda_{2}^{2} \right) \right)+\left( -1+u \right)u\left( -1+\lambda_{1}^{2} \right)\left( -1+\lambda_{2}^{2} \right)\mathrm{Cos}\left[ 2\left( \theta_{1}-\theta_{2} \right) \right]}$$

Straightforward calculation shows that $\partial_{u}y=0$ for $u_{max}= \frac{1}{1+\frac{\lambda_{1}}{\lambda_{2}}}$

Substituting this into the expression for $y$, we get that the maximal deviation for given parameters $\lambda_{1},\theta_{1}$ and $\lambda_{2},\theta_{2}$ is:

$h(\lambda_{1},\theta_{1}$ $\lambda_{2},\theta_{2})=$|

It is bounded by:

$$\left| -\frac{\left( -1+\lambda_{1}^{2} \right)\left( 1+\lambda_{2}^{2} \right)\mathrm{Sin}\left[ 2\theta_{1} \right]-\left( -1+\lambda_{2}^{2} \right)\left( \left( -1+\lambda_{1}^{2} \right)\mathrm{Sin}\left[ 2\left( \theta_{1}-\theta_{2} \right) \right]+\left( 1+\lambda_{1}^{2} \right)\mathrm{Sin}\left[ 2\theta_{2} \right] \right)}{2\left( 1+\lambda_{1}^{2}+4\lambda_{1}\lambda_{2}+\lambda_{2}^{2}+\lambda_{1}^{2}\lambda_{2}^{2}-\left( -1+\lambda_{1}^{2} \right)\left( -1+\lambda_{2}^{2} \right)\mathrm{Cos}\left[ 2\left( \theta_{1}-\theta_{2} \right) \right] \right)} \right|\leq\frac{\left| \left( -1+\lambda_{1}^{2} \right)\left( 1+\lambda_{2}^{2} \right)\mathrm{Sin}\left[ 2\theta_{1} \right] \right|+\left| \left( -1+\lambda_{2}^{2} \right)\left( -1+\lambda_{1}^{2} \right)\mathrm{Sin}\left[ 2\left( \theta_{1}-\theta_{2} \right) \right] \right|+\left| \left( 1+\lambda_{1}^{2} \right)\left( -1+\lambda_{2}^{2} \right)\mathrm{Sin}\left[ 2\theta_{2} \right] \right|}{2\left| \left| 1+\lambda_{1}^{2}+4\lambda_{1}\lambda_{2}+\lambda_{2}^{2}+\lambda_{1}^{2}\lambda_{2}^{2} \right|-\left| \left( -1+\lambda_{1}^{2} \right)\left( -1+\lambda_{2}^{2} \right)\mathrm{Cos}\left[ 2\left( \theta_{1}-\theta_{2} \right) \right] \right| \right|}= \frac{\left| \left( -1+\lambda_{1}^{2} \right)\left( 1+\lambda_{2}^{2} \right)\mathrm{Sin}\left[ 2\theta_{1} \right] \right|+\left| \left( -1+\lambda_{2}^{2} \right)\left( -1+\lambda_{1}^{2} \right)\mathrm{Sin}\left[ 2\left( \theta_{1}-\theta_{2} \right) \right] \right|+\left| \left( 1+\lambda_{1}^{2} \right)\left( -1+\lambda_{2}^{2} \right)\mathrm{Sin}\left[ 2\theta_{2} \right] \right|}{2(1+\lambda_{1}^{2}+4\lambda_{1}\lambda_{2}+\lambda_{2}^{2}+\lambda_{1}^{2}\lambda_{2}^{2}-\left( 1-\lambda_{1}^{2} \right)\left( 1-\lambda_{2}^{2} \right)\left| \mathrm{Cos}\left[ 2\left( \theta_{1}-\theta_{2} \right) \right] \right|)}$$

$$\leq\frac{\left( 1-\lambda_{1}^{2} \right)\left( 1+\lambda_{2}^{2} \right)+\left( 1-\lambda_{2}^{2} \right)\left( 1-\lambda_{1}^{2} \right)+\left( 1+\lambda_{1}^{2} \right)\left( 1-\lambda_{2}^{2} \right)}{2\left( 1+\lambda_{1}^{2}+4\lambda_{1}\lambda_{2}+\lambda_{2}^{2}+\lambda_{1}^{2}\lambda_{2}^{2}-\left( 1-\lambda_{1}^{2} \right)\left( 1-\lambda_{2}^{2} \right) \right)} = -\frac{-3+\lambda_{1}^{2}+\lambda_{2}^{2}+\lambda_{1}^{2}\lambda_{2}^{2}}{4\left( \lambda_{1}+\lambda_{2} \right)^{2}}$$

So, the maximal deviation from the line between the archetypes, for any given $\theta_{1}, \theta_{2}$ is bounded by $\frac{3-\lambda_{1}^{2}-\lambda_{2}^{2}-\lambda_{1}^{2}\lambda_{2}^{2}}{4\left( \lambda_{1}+\lambda_{2} \right)^{2}}$.

Let’s focus on the special case when one of the performance functions depends on Euclidean norm, i.e. – $\lambda_{1}=1$.

In this case the upper bound becomes $h\leq\frac{1-\lambda_{2}}{2(1+\lambda_{2})}$, and this is a tight bound:

$$h(1,\theta_{1}, \lambda_{2},\theta_{2})= \frac{\left( 1-\lambda_{2} \right)Sin[2\theta_{2}]}{2(1+\lambda_{2})}$$

When setting $\theta_{2}=\frac{\pi}{4}$ this bound is obtained (as expected, $\theta_{1}$ is irrelevant when $\lambda_{1}=1$).

Hence, when task 1 depends on Euclidean norm, the maximal deviation of the front per given $\lambda_{2}$ is obtained for $\theta_{2}=\frac{\pi}{4}$ and is given by:

$$h\left( 1,\theta_{1}, \lambda_{2},\theta_{2} \right)\leq\frac{(1-\lambda_{2})}{2(1+\lambda_{2})}$$

In this case, the deviation of the Pareto front from the line between the archetypes, maximized on all $\lambda_{2}'s$, and on all ${\theta_{2}}^{'}s$ is half the distance between the archetypes. The deviation approaches this value as $\lambda_{2}\to0$ (the contours of the second norm becomes more and more eccentric).

# Appendix 5

## Each Pareto front of 2 tasks in a 2D morphospace is generated by a 1-dimensional family of norm-pairs

Here, we would like to deal with the following question: Let *PF* be a Pareto front associated with 2 tasks in a 2D-morphospace, where each performance decays from its archetype with a different inner-product norm. In Appendix 2 we showed that under the above assumptions, the Pareto front is a segment of a hyperbola (or a line) that connects the archetypes $v_{1}^{*}$ and $v_{2}^{*}$. In that case, can we deduce the norms that the performance functions decay with from the exact shape of the Pareto front?

When approaching the question presented above, we assume that the position of the archetypes is known. This is a reasonable assumption – for a given hyperbola/line-shaped data set, the 2 edge points of the front are assumed to be the archetypes.

We will show that not all hyperbola-shaped datasets can be explained by the model with its current assumptions – there are hyperbolae that are not generated by any pair of norms. However, if for a hyperbola-shaped dataset there exists a pair of norms that generates it, then there exists a one-dimensional family of norm-pairs that generate it.

This is expected. A hyperbola is a quadratic curve defined by an equation of the form: $A_{xx}x^{2}+2A_{xy}xy+A_{yy}y^{2}+2A_{x}x+2A_{y}y+A=0$. Hence, it is defined by 5 free parameters (we can normalize the equation by one of the coefficients). We assume that the position of the archetypes is known. A hyperbola-shaped front must pass through the 2 archetypes, leaving it with 3 free parameters (each point yields a single equation the hyperbola’s coefficients must satisfy, reducing the number of free parameters by one). The norms that the performance functions depend on are represented by the matrices $M_{1}, M_{2}$, where $M_{i}=R\left( \theta_{i} \right)\left( \begin{matrix} 1 & 0 \\ 0 & \lambda_{i}^{2} \end{matrix} \right)R\left( -\theta_{i} \right)$ (Appendix 2). Hence, besides the position of the archetype $v_{i}^{*}$, each norm can be describes by 2 parameters - the ratio between the eigenvalues of the norm’s matrix, $\lambda_{i}^{2}$, and the angle of the rotation matrix that diagonalizes it, $\theta_{i}$ ($P_{i}=-\left( v-v_{i}^{*} \right)^{T}R\left( \theta_{i} \right)\left( \begin{matrix} 1 & 0 \\ 0 & \lambda_{i}^{2} \end{matrix} \right)R\left( -\theta_{i} \right)\left( v-v_{i}^{*} \right)$). It means that if the location of the archetypes is known, the 2 performance functions together have 4 free parameters. Thus, the problem of deducing the norms from the shape of the Pareto front is expectedly degenerate, since we try to determine 4 free parameters (the norms of the performance functions) using only 3 observed parameters (the hyperbola). Also, we expect the family of norm-pairs that generate each hyperbola to depend only on 1 parameter – i.e. - to be 1-dimensional.

Consider a hyperbola/line-shaped dataset that is generated by a pair of norms $M_{1}$ and $M_{2}$, with archetypes $v_{1}^{*},v_{2}^{*}$. We can transform to a coordinate system where $v_{1}^{*}=(0,0)$ and $v_{2}^{*}=(1,0)$, $M_{1}=I$ and $M_{2}$ is a positive definite matrix, using a transformation under which a hyperbola/line remains a hyperbola/line. Such transformation was shown to exist in Appendix 2. There is a 1-to-1 correspondence between norm-pairs that generate the transformed front in the transformed coordinate system and the norm-pairs that generate the front in the original coordinate system. Hence, if we show that there is a 1-dimensional family of norm pairs that generate the front in the transformed system, the conclusion will also hold in the original system.

Assume that in the transformed coordinate system, the quadratic curve that the Pareto front lies on is represented by:

$$A_{xx}x^{2}+2A_{xy}xy+A_{yy}y^{2}+2A_{x}x+2A_{y}y+A=0$$

We constrain the curve to go through the archetypes at $(0,0)$ and $\left( 1,0 \right)$, and get that $A=0$ and $A_{xx}= - 2A_{x}$

So we expect the Pareto front to be of the form

$$\left( PF \right) A_{xx}x^{2}+2A_{xy}xy+A_{yy}y^{2}-A_{xx}x+2A_{y}y=0$$

Each quadratic curve is associated with 2 parameters, $\Delta$ and $J$, defined by:

$J = A_{xx}A_{yy}$ - $A_{xy}^{2}$ and $\Delta=\det\left( \begin{matrix} A_{xx} & A_{xy} & A_{x} \\ A_{xy} & A_{yy} & A_{y} \\ A_{x} & A_{y} & A \end{matrix} \right)= -\frac{A_{xx}^{2}A_{yy}}{4}-A_{xx}A_{xy}A_{y}-A_{xx}A_{y}^{2}$

For a hyperbola $J<0$ and $\Delta\neq0$. For a line, $J=0$ and $\Delta=0$.

We would like to find all norm-pairs $(m_{1},m_{2})$ that generate $\left( PF \right)$, given that the pair $(I,M_{2})$ generates it.

Let $m_{1}$ depend on parameters $\lambda_{1},\theta_{1}$ and $m_{2}$ depend on parameters $\lambda_{2},\theta_{2}$. Namely -

$$m_{1}=R\left( \theta_{1} \right)\left( \begin{matrix} 1 & 0 \\ 0 & \lambda_{1}^{2} \end{matrix} \right)R\left( -\theta_{1} \right){; m}_{2}=R\left( \theta_{2} \right)\left( \begin{matrix} 1 & 0 \\ 0 & \lambda_{2}^{2} \end{matrix} \right)R\left( -\theta_{2} \right)$$

As will be shown in Appendix 7 (Lemma 1), the quadratic curve on which lies a Pareto front associated with 2 tasks can be given by the equation: ${\pi_{z}(g}_{1}(v)\times g_{2}(v))\equiv0$, where $g_{i}\left( v \right)=\nabla P_{i}\left( v \right)=m_{i}(v-v_{i}^{*})$. Denote $PF\left( \lambda_{1}, \lambda_{2},\theta_{1},\theta_{2} \right)={\pi_{z}(g}_{1}(v)\times g_{2}(v))\Rightarrow$

$$PF\left( \lambda_{1}, \lambda_{2},\theta_{1},\theta_{2} \right)= y \left( 1+\lambda_{1}^{2}+\lambda_{2}^{2}+\lambda_{1}^{2}\lambda_{2}^{2}+\left( -1+\lambda_{1}^{2} \right)\left( 1+\lambda_{2}^{2} \right)\mathrm{Cos}\left[ 2\theta_{1} \right]-\left( -1+\lambda_{1}^{2} \right)\left( -1+\lambda_{2}^{2} \right)\mathrm{Cos}\left[ 2\left( \theta_{1}-\theta_{2} \right) \right]-\left( 1+\lambda_{1}^{2} \right)\left( -1+\lambda_{2}^{2} \right)\mathrm{Cos}\left[ 2\theta_{2} \right] \right)+2xy\left( -\left( -1+\lambda_{1}^{2} \right)\left( 1+\lambda_{2}^{2} \right)\mathrm{Cos}\left[ 2\theta_{1} \right]+\left( 1+\lambda_{1}^{2} \right)\left( -1+\lambda_{2}^{2} \right)\mathrm{Cos}\left[ 2\theta_{2} \right] \right)+x\left( \left( -1+\lambda_{1}^{2} \right)\left( 1+\lambda_{2}^{2} \right)\mathrm{Sin}\left[ 2\theta_{1} \right]-\left( -1+\lambda1 \right)\left( -1+\lambda_{2}^{2} \right)\mathrm{Sin}\left[ 2\left( \theta_{1}-\theta_{2} \right) \right]-\left( 1+\lambda_{1}^{2} \right)\left( -1+\lambda_{2}^{2} \right)\mathrm{Sin}\left[ 2\theta_{2} \right] \right)+y^{2}\left( \left( -1+\lambda_{1}^{2} \right)\left( 1+\lambda_{2}^{2} \right)\mathrm{Sin}\left[ 2\theta_{1} \right]+\left( -1+\lambda_{1}^{2} \right)\left( -1+\lambda_{2}^{2} \right)\mathrm{Sin}\left[ 2\left( \theta_{1}-\theta_{2} \right) \right]-\left( 1+\lambda_{1}^{2} \right)\left( -1+\lambda_{2}^{2} \right)\mathrm{Sin}\left[ 2\theta_{2} \right] \right)+x^{2}(-(-1+\lambda_{1}^{2})(1+\lambda_{2}^{2})\mathrm{Sin}[2\theta_{1}]+(-1+\lambda_{1}^{2})(-1+\lambda_{2}^{2})\mathrm{Sin}[2(\theta_{1}-\theta_{2})]+(1+\lambda_{1}^{2})(-1+\lambda_{2}^{2})\mathrm{Sin}[2\theta_{2}])$$

The Pareto front associated with $m_{1},m_{2}$ is thus given by $PF\left( \lambda_{1}, \lambda_{2},\theta_{1},\theta_{2} \right)=0$.

Here we observe the invariance of the Pareto front under $\lambda_{i}\to\frac{1}{\lambda_{i}}, \theta_{i}\to\theta_{i}+\frac{\pi}{2}$, which is inherent to the problem since the norms are symmetric under this transformation. This is only a “technical” degeneracy rather than a genuine one, since $d_{i}(\lambda_{i}$,$\theta_{i}$) is essentially the same as $d_{i}(\frac{1}{\lambda_{i}}, \theta_{i}+\frac{\pi}{2}$). The resulting norms only differ by a factor of $\lambda$, and generate the same contours and the same Pareto front (see Appendix 2). Hence, we can assume $0\leq\theta<\frac{\pi}{2}$.

We would like to know when $PF=0$ and $PF\left( \lambda_{1}, \lambda_{2},\theta_{1},\theta_{2} \right)=0$ represent the same line/hyperbola. This happens if and only if these quadratic forms are equal up to a factor, i.e. -$PF=\beta PF\left( \lambda_{1}, \lambda_{2},\theta_{1},\theta_{2} \right).$

We know that $PF$ and $PF\left( \lambda_{1}=1, \lambda,\theta_{1}=0,\theta\right)$ represent the same quadratic curve. Hence, $\exists\beta s.t. PF=\beta PF\left( \lambda_{1}=1, \lambda,\theta_{1}=0,\theta\right)$. Examining the expression for $PF\left( \lambda_{1}=1, \lambda,\theta_{1}=0,\theta\right)$, we find out that the coefficients of $x^{2}$ and of $y^{2}$ are opposite to one another. That is – if the hyperbola was indeed generated by the given norm-pair, it mush have $A_{xx}=-A_{yy}$.

First, let’s find which norm pairs generate $PF$ given that it is a line. If the front is a line, then both $J=0$ and $\Delta=0$. As $A_{xx}=-A_{yy}$ and $J= -A_{yy}^{2}-A_{xy}^{2}$ we get that $A_{yy}=0$ and $A_{xy}=0$

We look for all other $\lambda_{1}, \lambda_{2},\theta_{1},\theta_{2}$, for which exists $\beta$ such that $PF\left( \lambda_{1}, \lambda_{2},\theta_{1},\theta_{2} \right)=\beta PF$.

We know that when this happens, the coefficients of $x^{2}$ and of $y^{2}$ are opposite to one another. This results in the equation:

$$\left( A \right)-2\left( -1+\lambda_{1} \right)\left( -1+\lambda_{2} \right)\mathrm{Sin}\left[ 2\left( \theta_{1}-\theta_{2} \right) \right]=0$$

This equation is satisfied if $\lambda_{1}=1$, $\lambda_{2}=1$ or $\theta_{1}=\theta_{2}$ as all angles are taken modulo $\frac{\pi}{2}$.

Also, we know that the coefficients of $x^{2}$, $y^{2}$ and $xy$ are zero. This results in the following equation:

$$\left( B \right)\left( \left( -1+\lambda_{1}^{2} \right)\left( 1+\lambda_{2}^{2} \right)\mathrm{Sin}\left[ 2\theta_{1} \right]+\left( -1+\lambda_{1}^{2} \right)\left( -1+\lambda_{2}^{2} \right)\mathrm{Sin}\left[ 2\left( \theta_{1}-\theta_{2} \right) \right]-\left( 1+\lambda_{1}^{2} \right)\left( -1+\lambda_{2}^{2} \right)\mathrm{Sin}\left[ 2\theta_{2} \right] \right)=0$$

Substituting into $(B)$ each of the solutions found for equation $(A)$, we get that:

If $\lambda_{1}=1,$ equation (B) becomes: $-2\left( -1+\lambda_{2} \right)\mathrm{Sin}\left[ 2\theta_{2} \right]=0$. It is satisfied if $\lambda_{2}=1$ or $\theta_{2}=0$ (as all angles are taken modulo $\frac{\pi}{2}$).

If $\lambda_{2}=1,$ equation (B) becomes: $2\left( 1-\lambda_{1} \right)\mathrm{Sin}\left[ 2\theta_{1} \right]=0$. It is satisfied if $\lambda_{1}=1$ or $\theta_{1}=0$.

If $\theta_{1}=\theta_{2}$, equation (B) becomes: $2\left( \lambda_{1}-\lambda_{2} \right)\mathrm{Sin}\left[ 2\theta_{2} \right]=0$. It is satisfied if $\theta_{2}=0$, or $\lambda_{1}-\lambda_{2}=0$

To conclude, equations $(A)$ and $(B$), which must be satisfied in order that $PF\left( \lambda_{1}, \lambda_{2},\theta_{1},\theta_{2} \right)$ will represents the same line as $PF$, are satisfied if and only if:

1. $\lambda_{1}=\lambda_{2}$ and $\theta_{1}=\theta_{2}$

$$or$$

$\theta_{1}=\theta_{2}=0$

Note that if $\lambda_{i}=1$, $\theta_{i}$ has no effect on the norm and we can take it to be whatever fits.

On the other hand, it can be easily checked that if one of those conditions holds, $PF\left( \lambda_{1}, \lambda_{2},\theta_{1},\theta_{2} \right)$ represents a line (or intersecting lines) that the line between the archetypes lies on.

It means that 2 norms generate the line between the archetypes if and only if one of the above conditions holds. The meaning of (I) is that $m_{1}=m_{2}$, and (II) means that $m_{1}'s$ and $m_{2}'s$ elliptic contours each have an axis parallel to the $x$ axis (which is parallel to $v_{1}^{*}-v_{2}^{*}$)

Note that if a dataset is shaped like a line, 2 Euclidean norms will always generate it. This means that in order for the above conclusions to hold, we only need to translate, rotate and isometrically scale the coordinate system such that the archetypes are at $(0,0)$ and $(1,0)$. This implies that the above conclusions also hold in the original coordinate system. Equality of matrices is not affected by change of basis, which covers (I). Regarding case (II) – notice that the contours’ axes transform like regular vectors when applying translations / rotations, and parallel vectors remain parallel under any linear transformation.

To summarize – for a given archetype pair ($v_{1}^{*},v_{2}^{*})$, the norm-pair $(m_{1}, m_{2})$ generates a Pareto front which is the line between them if and only if $m_{1}=m_{2}$ or both $m_{1}$ and $m_{2}$ have elliptic contours with an axis that is parallel to $v_{1}^{*}-v_{2}^{*}$.

After taking care of which norms generate a line-shaped front, we will assume from now on that the front is not a straight line but a hyperbola.

We saw that on the frame we work on, the Pareto front lies on a curve given by the equation:

$$A_{xx}x^{2}+2A_{xy}xy-A_{xx}y^{2}-A_{xx}x+2A_{y}y=0$$

We know that $A_{xx}\neq0$, since otherwise $\Delta=0$ and the resulting Pareto front is not a hyperbola, so we can normalize the coefficient of $x^{2}$ to be 1, and equate both (normalized) representations:

$$x^{2}-x+2A_{xy}xy-y^{2}+2A_{y}y=0$$

And

$$x^{2}-x+ y\frac{\left( 1+\lambda_{1}^{2}+\lambda_{2}^{2}+\lambda_{1}^{2}\lambda_{2}^{2}+\left( -1+\lambda_{1}^{2} \right)\left( 1+\lambda_{2}^{2} \right)\mathrm{Cos}\left[ 2\theta_{1} \right]-\left( -1+\lambda_{1}^{2} \right)\left( -1+\lambda_{2}^{2} \right)\mathrm{Cos}\left[ 2\left( \theta_{1}-\theta_{2} \right) \right]-\left( 1+\lambda_{1}^{2} \right)\left( -1+\lambda_{2}^{2} \right)\mathrm{Cos}\left[ 2\theta_{2} \right] \right)}{\left( -\left( -1+\lambda_{1}^{2} \right)\left( 1+\lambda_{2}^{2} \right)\mathrm{Sin}\left[ 2\theta_{1} \right]+\left( -1+\lambda_{1}^{2} \right)\left( -1+\lambda_{2}^{2} \right)\mathrm{Sin}\left[ 2\left( \theta_{1}-\theta_{2} \right) \right]+\left( 1+\lambda_{1}^{2} \right)\left( -1+\lambda_{2}^{2} \right)\mathrm{Sin}\left[ 2\theta_{2} \right] \right)}+2xy\frac{\left( -\left( -1+\lambda_{1}^{2} \right)\left( 1+\lambda_{2}^{2} \right)\mathrm{Cos}\left[ 2\theta_{1} \right]+\left( 1+\lambda_{1}^{2} \right)\left( -1+\lambda_{2}^{2} \right)\mathrm{Cos}\left[ 2\theta_{2} \right] \right)}{\left( -\left( -1+\lambda_{1}^{2} \right)\left( 1+\lambda_{2}^{2} \right)\mathrm{Sin}\left[ 2\theta_{1} \right]+\left( -1+\lambda_{1}^{2} \right)\left( -1+\lambda_{2}^{2} \right)\mathrm{Sin}\left[ 2\left( \theta_{1}-\theta_{2} \right) \right]+\left( 1+\lambda_{1}^{2} \right)\left( -1+\lambda_{2}^{2} \right)\mathrm{Sin}\left[ 2\theta_{2} \right] \right)}+y^{2}\frac{\left( \left( -1+\lambda_{1}^{2} \right)\left( 1+\lambda_{2}^{2} \right)\mathrm{Sin}\left[ 2\theta_{1} \right]+\left( -1+\lambda_{1}^{2} \right)\left( -1+\lambda_{2}^{2} \right)\mathrm{Sin}\left[ 2\left( \theta_{1}-\theta_{2} \right) \right]-\left( 1+\lambda_{1}^{2} \right)\left( -1+\lambda_{2}^{2} \right)\mathrm{Sin}\left[ 2\theta_{2} \right] \right)}{\left( -\left( -1+\lambda_{1}^{2} \right)\left( 1+\lambda_{2}^{2} \right)\mathrm{Sin}\left[ 2\theta_{1} \right]+\left( -1+\lambda_{1}^{2} \right)\left( -1+\lambda_{2}^{2} \right)\mathrm{Sin}\left[ 2\left( \theta_{1}-\theta_{2} \right) \right]+\left( 1+\lambda_{1}^{2} \right)\left( -1+\lambda_{2}^{2} \right)\mathrm{Sin}\left[ 2\theta_{2} \right] \right)}=0$$

These normalized representations describe the same hyperbola if and only if the coefficients are equal. This results in 3 equations (equation set E1):

$-1+\frac{\left( -1+\lambda_{1}^{2} \right)\left( 1+\lambda_{2}^{2} \right)\mathrm{Sin}\left[ 2\theta_{1} \right]+\left( -1+\lambda_{1}^{2} \right)\left( -1+\lambda_{2}^{2} \right)\mathrm{Sin}\left[ 2\left( \theta_{1}-\theta_{2} \right) \right]-\left( 1+\lambda_{1}^{2} \right)\left( -1+\lambda_{2}^{2} \right)\mathrm{Sin}\left[ 2\theta_{2} \right]}{\left( 1-\lambda_{1}^{2} \right)\left( 1+\lambda_{2}^{2} \right)\mathrm{Sin}\left[ 2\theta_{1} \right]+\left( -1+\lambda_{1}^{2} \right)\left( -1+\lambda_{2}^{2} \right)\mathrm{Sin}\left[ 2\left( \theta_{1}-\theta_{2} \right) \right]+\left( 1+\lambda_{1}^{2} \right)\left( -1+\lambda_{2}^{2} \right)\mathrm{Sin}\left[ 2\theta_{2} \right]}=0$

$-2A_{y}+\frac{\left( 1+\lambda_{1}^{2} \right)\left( 1+\lambda_{2}^{2} \right)+\left( -1+\lambda_{1}^{2} \right)\left( 1+\lambda_{2}^{2} \right)\mathrm{Cos}\left[ 2\theta_{1} \right]-\left( -1+\lambda_{1}^{2} \right)\left( -1+\lambda_{2}^{2} \right)\mathrm{Cos}\left[ 2\left( \theta_{1}-\theta2 \right) \right]-\left( 1+\lambda_{1}^{2} \right)\left( -1+\lambda_{2}^{2} \right)\mathrm{Cos}\left[ 2\theta_{2} \right]}{\left( 1-\lambda_{1}^{2} \right)\left( 1+\lambda_{2}^{2} \right)\mathrm{Sin}\left[ 2\theta_{1} \right]+\left( -1+\lambda_{2}^{2} \right)\left( \left( -1+\lambda_{1}^{2} \right)\mathrm{Sin}\left[ 2\left( \theta_{1}-\theta_{2} \right) \right]+\left( 1+\lambda_{1}^{2} \right)\mathrm{Sin}\left[ {2\theta}_{2} \right] \right)}=0$

$-A_{xy}+\frac{\left( 1-\lambda_{1}^{2} \right)\left( 1+\lambda_{2}^{2} \right)\mathrm{Cos}\left[ 2\theta_{1} \right]+\left( 1+\lambda_{1}^{2} \right)\left( -1+\lambda_{2}^{2} \right)\mathrm{Cos}\left[ {2\theta}_{2} \right]}{\left( 1-\lambda_{1}^{2} \right)\left( 1+\lambda_{2}^{2} \right)\mathrm{Sin}\left[ 2\theta_{1} \right]+\left( -1+\lambda_{2}^{2} \right)\left( \left( -1+\lambda_{1}^{2} \right)\mathrm{Sin}\left[ 2\left( \theta_{1}-\theta_{2} \right) \right]+\left( 1+\lambda_{1}^{2} \right)\mathrm{Sin}\left[ {2\theta}_{2} \right] \right)}=0$

Consider equation (I):

$$\frac{2(-1+\lambda_{1}^{2})(-1+\lambda_{2}^{2})\mathrm{Sin}[2(\theta_{1}-\theta_{2})]}{-(-1+\lambda_{1}^{2})(1+\lambda_{2}^{2})\mathrm{Sin}[2\theta_{1}]+(-1+\lambda_{2}^{2})((-1+\lambda_{1}^{2})\mathrm{Sin}[2(\theta_{1}-\theta_{2})]+(1+\lambda_{1}^{2})\mathrm{Sin}[2\theta_{2}])}=0$$

It is satisfied when $\lambda_{1}^{2}=1$ or $\lambda_{2}^{2}=1$ or $\theta_{1}=\theta_{2}$. Substituting either $\lambda_{i}=1$ into equations (I) and (III) yields equations that don’t depend on $\theta_{i}$ (this is expected since in case $\lambda_{i}=1$ the respective norm is Euclidean and $\theta_{i}$ is meaningless). So it is safe to assume that in this case it is true, in a sense, that $\theta_{1}= \theta_{2}$. From now on we’ll simply use $\theta$ to denote the common angle. Equations (I) and (II) now become (regardless of which condition satisfied equation (I)):

$$-2A_{xy}+2\cot\left[ 2\theta\right]=0$$

$$\frac{{2A}_{y}(\lambda_{1}^{2}-\lambda_{2}^{2})+\lambda_{1}^{2}\cot\left[ \theta\right]+\lambda_{2}^{2}\tan[\theta]}{-\lambda_{1}^{2}+\lambda_{2}^{2}}=0$$

This means that $\theta:=\theta_{1}=\theta_{2}=\frac{\mathrm{arccot} \left[ A_{xy} \right]}{2}$

For the second equation we see that it reduces to

$${2A}_{y}(1-\frac{\lambda_{2}^{2}}{\lambda_{1}^{2}})+\cot[\theta]+\frac{\lambda_{2}^{2}}{\lambda_{1}^{2}}\tan[\theta]=0$$

It means that from this equation we can only deduce $\left( \frac{\lambda_{2}}{\lambda_{1}} \right)^{2}$. Denote $C=\left( \frac{\lambda_{2}}{\lambda_{1}} \right)^{2}$. *C* depends on the parameters of the hyperbola:

$$C=\frac{-2A_{y}-\cot[\frac{\mathrm{arccot}[A_{xy}]}{2}]}{-2A_{y}+\tan[\frac{\mathrm{arccot}[A_{xy}]}{2}]}$$

Note that for consistency, the value deduced for *C* from equation (II) must be positive. If $C\leq0$, it means that there is no pair of norms that generate this hyperbola. We know that the Pareto front is generated by $m_{1}=I, m_{2}=M_{2}$, so their parameters must satisfy equation (II). $\lambda_{1}=1,\lambda_{2}=\lambda, \theta_{1}=0, \theta_{2}=\theta\Rightarrow{2A}_{y}\left( 1-\lambda\right)+\cot[\frac{\mathrm{arccot}[A_{xy}]}{2}]+\lambda\tan[\frac{\mathrm{arccot}[A_{xy}]}{2}]=0$

$$\Rightarrow\lambda^{2}=\frac{-2A_{y}-\cot[\frac{\mathrm{arccot}[A_{xy}]}{2}]}{-2A_{y}+\tan[\frac{\mathrm{arccot}[A_{xy}]}{2}]}=C$$

$\lambda^{2}>0$, therefore $C>0$. This means that for every choice of $\lambda_{1}>0$, choosing $\lambda_{2}^{2}=C \lambda_{1}^{2}$ will result in $\lambda_{2}^{2}$ greater than zero, and hence $m_{2}$ that depends on $\lambda_{2}$ will represent an inner-product norm. Hence, the parameters $\theta_{1}=\theta_{2}= \frac{\mathrm{arccot} [A_{xy}]}{2}$, $\lambda_{1}, \lambda_{2}= \sqrt{C}\lambda_{1}$ define 2 norms, $m_{1}$ and $m_{2}$ that generate (PF), for every $\lambda_{1}>0$. This means that there is a 1-dimensional family of norm-pairs that generate *PF,* parameterized by $\lambda_{1}$. Note that choosing $m_{1}$ determines $m_{2}$ uniquely.

However, note that there exist hyperbola-shaped data sets that cannot be describes as a Pareto front associated with 2 tasks. This is since the existence of any solution relies on *C* being positive. *C* is defined by the parameters of the hyperbola ($C=\frac{-2A_{y}-\cot[\frac{\mathrm{arccot}[A_{xy}]}{2}]}{-2A_{y}+\tan[\frac{\mathrm{arccot}[A_{xy}]}{2}]}$). We can find a hyperbola with parameters that define a negative *C,* which means that it is not the Pareto front of any norm pairs. This also means that given a hyperbola, it is easy to check whether or not it is generated by a norm-pair by simply calculating $C$. An example for a hyperbola with $C<0$ is $x^{2}-2xy-y^{2}-x+2y=0$.

So, knowing at least one norm pair that generates the Pareto front, we can deduce all norm pairs. However, another question can be asked: can we determine from the parameters of the hyperbola whether there are norm pairs that generate the hyperbola, and if so what are the norms? We will show that in the common case, it is numerically possible.

To approach this question, assume we have data shaped like a hyperbola section. From the data, we can identify the edge points of the hyperbola, and change coordinate system such that the edge points are at $(0,0)$ and $(1,0)$. Those points are assumed to be the 2 archetypes - $v_{1}^{*}=(0,0)$ and $v_{2}^{*}=(1,0)$. The new coordinate system results from the old coordinate system by a rotation, translation and isometric scaling. Note the difference between this transformation and the one described earlier – in both of them we transform the archetypes to be at $(0,0)$ and $(1,0)$. However, earlier we transformed such that one of the norms that generates the front is Euclidean. Here we can’t do so as we don’t know which norm pairs generate the hyperbola. As before, we get that under such transformations, a hyperbola remains a hyperbola, and there is a 1-to-1 correspondence between norm-pairs that generate the transformed front in the transformed coordinate system and the norm-pairs that generate the front in the original coordinate system. Hence, once we find which norms generate the front in the transformed coordinate system, we can transform back and find the norms that generate the front in the original coordinate system. If we find that there isn’t a norm pair that generates the hyperbola in the transformed coordinate system, this conclusion will hold in the original coordinate system.

In this coordinate system, the hyperbola is given by the equation

$$A_{xx}x^{2}+2A_{xy}xy+A_{yy}y^{2}-A_{xx}x+2A_{y}y=0$$

The $x$ coefficient and free parameter are determined since the hyperbola has to pass through the archetypes at (0,0) and (0,1).

We would like to know which pairs of norms, if exist, generate a Pareto front with those parameters. We search for parameters $\lambda_{1}, \lambda_{2},\theta_{1},\theta_{2}$ such that $PF(\lambda_{1}, \lambda_{2},\theta_{1},\theta_{2})$, defined before, describes the same curve as $A_{xx}x^{2}+2A_{xy}xy+A_{yy}y^{2}-A_{xx}x+2A_{y}y=0$.

We know that $A_{xx}\neq0$, since otherwise $\Delta=0$ and the Pareto front is a line, so we can normalize the coefficient of $x^{2}$ to be 1, and equate both (normalized) representations:

$$x^{2}-x+2A_{xy}xy+A_{yy}y^{2}+2A_{y}y=0$$

and

$$x^{2}-x+ y\frac{\left( 1+\lambda_{1}^{2}+\lambda_{2}^{2}+\lambda_{1}^{2}\lambda_{2}^{2}+\left( -1+\lambda_{1}^{2} \right)\left( 1+\lambda_{2}^{2} \right)\mathrm{Cos}\left[ 2\theta_{1} \right]-\left( -1+\lambda_{1}^{2} \right)\left( -1+\lambda_{2}^{2} \right)\mathrm{Cos}\left[ 2\left( \theta_{1}-\theta_{2} \right) \right]-\left( 1+\lambda_{1}^{2} \right)\left( -1+\lambda_{2}^{2} \right)\mathrm{Cos}\left[ 2\theta_{2} \right] \right)}{\left( -\left( -1+\lambda_{1}^{2} \right)\left( 1+\lambda_{2}^{2} \right)\mathrm{Sin}\left[ 2\theta_{1} \right]+\left( -1+\lambda_{1}^{2} \right)\left( -1+\lambda_{2}^{2} \right)\mathrm{Sin}\left[ 2\left( \theta_{1}-\theta_{2} \right) \right]+\left( 1+\lambda_{1}^{2} \right)\left( -1+\lambda_{2}^{2} \right)\mathrm{Sin}\left[ 2\theta_{2} \right] \right)}+2xy\frac{\left( -\left( -1+\lambda_{1}^{2} \right)\left( 1+\lambda_{2}^{2} \right)\mathrm{Cos}\left[ 2\theta_{1} \right]+\left( 1+\lambda_{1}^{2} \right)\left( -1+\lambda_{2}^{2} \right)\mathrm{Cos}\left[ 2\theta_{2} \right] \right)}{\left( -\left( -1+\lambda_{1}^{2} \right)\left( 1+\lambda_{2}^{2} \right)\mathrm{Sin}\left[ 2\theta_{1} \right]+\left( -1+\lambda_{1}^{2} \right)\left( -1+\lambda_{2}^{2} \right)\mathrm{Sin}\left[ 2\left( \theta_{1}-\theta_{2} \right) \right]+\left( 1+\lambda_{1}^{2} \right)\left( -1+\lambda_{2}^{2} \right)\mathrm{Sin}\left[ 2\theta_{2} \right] \right)}+y^{2}\frac{\left( \left( -1+\lambda_{1}^{2} \right)\left( 1+\lambda_{2}^{2} \right)\mathrm{Sin}\left[ 2\theta_{1} \right]+\left( -1+\lambda_{1}^{2} \right)\left( -1+\lambda_{2}^{2} \right)\mathrm{Sin}\left[ 2\left( \theta_{1}-\theta_{2} \right) \right]-\left( 1+\lambda_{1}^{2} \right)\left( -1+\lambda_{2}^{2} \right)\mathrm{Sin}\left[ 2\theta_{2} \right] \right)}{\left( -\left( -1+\lambda_{1}^{2} \right)\left( 1+\lambda_{2}^{2} \right)\mathrm{Sin}\left[ 2\theta_{1} \right]+\left( -1+\lambda_{1}^{2} \right)\left( -1+\lambda_{2}^{2} \right)\mathrm{Sin}\left[ 2\left( \theta_{1}-\theta_{2} \right) \right]+\left( 1+\lambda_{1}^{2} \right)\left( -1+\lambda_{2}^{2} \right)\mathrm{Sin}\left[ 2\theta_{2} \right] \right)}=0$$

These normalized representations describe the same hyperbola if and only if the coefficients are equal. This results in 3 equations (equation set E2):

$-A_{yy}+\frac{\left( -1+\lambda_{1}^{2} \right)\left( 1+\lambda_{2}^{2} \right)\mathrm{Sin}\left[ 2\theta_{1} \right]+\left( -1+\lambda_{1}^{2} \right)\left( -1+\lambda_{2}^{2} \right)\mathrm{Sin}\left[ 2\left( \theta_{1}-\theta_{2} \right) \right]-\left( 1+\lambda_{1}^{2} \right)\left( -1+\lambda_{2}^{2} \right)\mathrm{Sin}\left[ 2\theta_{2} \right]}{\left( 1-\lambda_{1}^{2} \right)\left( 1+\lambda_{2}^{2} \right)\mathrm{Sin}\left[ 2\theta_{1} \right]+\left( -1+\lambda_{1}^{2} \right)\left( -1+\lambda_{2}^{2} \right)\mathrm{Sin}\left[ 2\left( \theta_{1}-\theta_{2} \right) \right]+\left( 1+\lambda_{1}^{2} \right)\left( -1+\lambda_{2}^{2} \right)\mathrm{Sin}\left[ 2\theta_{2} \right]}=0$

$-2A_{y}+\frac{\left( 1+\lambda_{1}^{2} \right)\left( 1+\lambda_{2}^{2} \right)+\left( -1+\lambda_{1}^{2} \right)\left( 1+\lambda_{2}^{2} \right)\mathrm{Cos}\left[ 2\theta_{1} \right]-\left( -1+\lambda_{1}^{2} \right)\left( -1+\lambda_{2}^{2} \right)\mathrm{Cos}\left[ 2\left( \theta_{1}-\theta2 \right) \right]-\left( 1+\lambda_{1}^{2} \right)\left( -1+\lambda_{2}^{2} \right)\mathrm{Cos}\left[ 2\theta_{2} \right]}{\left( 1-\lambda_{1}^{2} \right)\left( 1+\lambda_{2}^{2} \right)\mathrm{Sin}\left[ 2\theta_{1} \right]+\left( -1+\lambda_{2}^{2} \right)\left( \left( -1+\lambda_{1}^{2} \right)\mathrm{Sin}\left[ 2\left( \theta_{1}-\theta_{2} \right) \right]+\left( 1+\lambda_{1}^{2} \right)\mathrm{Sin}\left[ {2\theta}_{2} \right] \right)}=0$

$-A_{xy}+\frac{\left( 1-\lambda_{1}^{2} \right)\left( 1+\lambda_{2}^{2} \right)\mathrm{Cos}\left[ 2\theta_{1} \right]+\left( 1+\lambda_{1}^{2} \right)\left( -1+\lambda_{2}^{2} \right)\mathrm{Cos}\left[ {2\theta}_{2} \right]}{\left( 1-\lambda_{1}^{2} \right)\left( 1+\lambda_{2}^{2} \right)\mathrm{Sin}\left[ 2\theta_{1} \right]+\left( -1+\lambda_{2}^{2} \right)\left( \left( -1+\lambda_{1}^{2} \right)\mathrm{Sin}\left[ 2\left( \theta_{1}-\theta_{2} \right) \right]+\left( 1+\lambda_{1}^{2} \right)\mathrm{Sin}\left[ {2\theta}_{2} \right] \right)}=0$

The solution of this equation set behaves quite differently depending on $A_{yy}$.

First assume that $A_{yy}=-1$. In that case, equation set E2 becomes equation set E1. Note that as mentioned under equation set E1, this scenario happens if $\theta_{1}=\theta_{2}, \lambda_{1}=1$ or $\lambda_{2}=1$ (one of the norms is Euclidean). We know that the resulting solution is as calculated above for E1, and that it is valid only if the hyperbola’s parameters are such that *C* > 0. Here, given only the fit of the hyperbola, we can determine if there are norms that generate the hyperbola, and what are the norms if they exist.

When $A_{yy}\neq-1$, solving equations (E2.I)+(E2.III) results in:

$$\lambda_{1}^{2}=1+\frac{2\left( 1+A_{yy} \right)}{-1-A_{yy}+\left( -1+A_{yy} \right)\mathrm{Cos}\left[ 2\theta_{1} \right]+2A_{xy}\mathrm{Sin}\left[ 2\theta_{1} \right]}$$

$$\lambda_{2}^{2}=1+\frac{2(1+A_{yy})}{-1-A_{yy}+(-1+A_{yy})\mathrm{Cos}[2\theta_{2}]+2A_{xy}\mathrm{Sin}[2\theta_{2}]}$$

Substituting this into equation (II) we get:

(IV)$\mathrm{Sin}\left[ {2\theta}_{1} \right]\left( 4\left( A_{xy}+A_{y}+A_{yy}A_{y} \right)\mathrm{Cos}\left[ 2\theta_{2} \right]+\left( -4{A_{xy}}^{2}+\left( 1+A_{yy} \right)^{2} \right)\mathrm{Sin}\left[ 2\theta_{2} \right] \right)+4 \mathrm{Cos}\left[ {2\theta}_{1} \right] (A_{yy}\mathrm{Cos}\left[ {2\theta}_{2} \right]-(A_{xy} A_{yy}+A_{y}+A_{yy} A_{y})\mathrm{Sin}[{2\theta}_{2}])=0$

For every $\theta_{2}$, we will show that there exists a single angle $\theta_{1}$ (modulo $\frac{\pi}{2}$), such that equation (IV) is satisfied. (An example for specific parameters $A_{yy},A_{xy},A_{y}$ is shown in Fig.S2)

For convenience denote $\xi:=\left( 1+A_{yy} \right)^{2}-4{A_{xy}}^{2}$, $\eta=4\left( A_{xy}+A_{y}+A_{yy}A_{y} \right)$

There are 2 cases:

(A) $\theta_{2}\neq\frac{\pi}{4} (mod\frac{\pi}{2})$:

Now $C\mathrm{os} \left[ {2\theta}_{2} \right]\neq0$ and we can divide the equation by it:

$\left( \mathrm{Sin}\left[ 2\theta_{1} \right]\left( \eta+\xi\mathrm{Tan} \left[ {2\theta}_{2} \right] \right)+4 \mathrm{Cos}\left[ {2\theta}_{1} \right] (A_{yy}\mathrm{Cos}\left[ {2\theta}_{2} \right]-(A_{xy} A_{yy}+A_{y}+A_{yy} A_{y})\mathrm{Sin}[{2\theta}_{2}]) \right)=0$

Again, there are 2 cases:

(A.1) $\mathrm{Tan} \left[ {2\theta}_{2} \right]=-\frac{\eta}{\xi}$

Substituting this into equation (IV) we get the equation:

$$\left( \frac{8\left( 1+A_{yy} \right)^{2}\left( A_{yy}+{4A}_{y}\left( A_{xy}+A_{y} \right) \right)}{\xi} \right)\mathrm{Cos}\left[ {2\theta}_{1} \right]=0$$

$\left( 1+A_{yy} \right)^{2}\left( A_{yy}+4A_{y}\left( A_{xy}+A_{y} \right) \right)\neq0$. This is because we assume that $A_{yy}\neq-1$, and, in addition, the $\Delta$ property of the quadratic curve is $\Delta=4(A_{yy}+{4A}_{y}\left( A_{xy}+A_{y} \right))$ and for a hyperbola $\Delta\neq0$. Hence, $\cos\left[ {2\theta}_{1} \right]$ must equal 0 for the equation to be satisfied, meaning $\theta_{1}=\frac{\pi}{4}(mod\frac{\pi}{2})$.

(A.2) $\mathrm{Tan} \left[ {2\theta}_{2} \right]\neq-\frac{\eta}{\xi}$:

In that case, we can assume that $\theta_{1}\neq\frac{\pi}{4}$. This is because when $\theta_{1}=\frac{\pi}{4}$, $\mathrm{Cos} \left[ 2\theta_{1} \right]=0$, $\mathrm{Sin} \left[ 2\theta_{1} \right]=1$, and $\theta_{1}=\frac{\pi}{4}$ solves the equation only if $\eta+\xi\mathrm{Tan} \left[ {2\theta}_{2} \right]=0$. Note that either $\xi\neq0$ or $\eta\neq0$, since if both $\xi=0$ and $\eta=0$, it can be shown that the $\Delta$ property of the quadratic curve becomes 0. This means that this equation has a solution only if $\mathrm{Tan}\left[ {2\theta}_{2} \right]=-\frac{\eta}{\xi}$, which is assumed not to be case.

So we assume $\theta_{1}\neq\frac{\pi}{4}$. In that case, $\mathrm{Cos} \left[ 2\theta_{1} \right]\neq0$ and we can divide the equation by it. The equation becomes:

$$8(A_{yy}-\left( A_{xy}A_{yy}+A_{y}+A_{yy}A_{y} \right))\mathrm{Tan} \left[ {2\theta}_{2} \right]+\mathrm{Tan}\left[ {2\theta}_{1} \right]\left( \eta+\xi\mathrm{Tan} \left[ {2\theta}_{2} \right] \right)=0$$

Note that if $\eta+\xi\mathrm{Tan} \left[ {2\theta}_{2} \right]\neq0$, as was shown earlier to be the case here, then $\theta_{1}$ such that:

$$\mathrm{Tan} \left[ {2\theta}_{1} \right]= \frac{-4A_{yy}+4(A_{y}+A_{yy}A_{xy}+A_{yy}A_{y})\mathrm{Tan} \left[ {2\theta}_{2} \right]}{\left( \eta+\xi\mathrm{Tan} \left[ {2\theta}_{2} \right] \right)}$$

solves equation (IV).

Note that once $\mathrm{Tan} [2\theta_{1}]$ is determined, $\theta_{1}$ is determined modulo $\frac{\pi}{2}$. However, we assume that $0\leq\theta_{1},\theta_{2}<\frac{\pi}{2}$, so determining $\theta_{1}$ up to $\frac{\pi}{2}$ is enough. Moreover, we know that if $\theta_{1}\to\theta_{1}+\frac{\pi}{2}$, then if also $\lambda_{1}\to\frac{1}{\lambda_{1}}$ the Pareto front doesn’t change. Examining the term for $\lambda_{1}$, we see this symmetry.

To conclude, for each $\theta_{2}\neq\frac{\pi}{4}$, there is a single $\theta_{1}$ in the range $0\leq\theta_{1}<\frac{\pi}{2}$ such that $\theta_{1},\theta_{2}, \lambda\left( \theta_{1} \right), \lambda(\theta_{2})$ generate the hyperbola.

(B) $\theta_{2}=\frac{\pi}{4}$:

In that case, $\theta_{1}=\frac{\pi}{4}$ is a solution to the equation if and only if $\xi=0$.

Otherwise, $\theta_{1}\neq\frac{\pi}{4}$, and we can divide by $\cos\left[ 2\theta_{1} \right]$ to get:

$2(\eta+\xi\tan[2\theta_{1}])$ = 0

Again, for a hyperbola, it is not possible that both $\eta$ and $\xi$ are zero, so the only way that the equation will be satisfied is if $\tan\left[ 2\theta_{1} \right]=\frac{\eta}{\xi}$.

To conclude, for every value of $\theta_{2}$, we can determine a unique value for $\theta_{1}$ in the range $[0,\frac{\pi}{2})$ such that the hyperbola on which lies the Pareto front that is generated by the 2 norms $M_{1}\left( {\lambda_{1}(\theta}_{1} \right),\theta_{1})$, $M_{2}\left( {\lambda_{2}(\theta}_{2} \right),\theta_{2})$ is the same as the hyperbola described by $x^{2}-x+2A_{xy}xy+A_{yy}y^{2}+{2A}_{y}y=0$. The $\theta_{1}$ that matches $\theta_{2}$ is given by:

$$\theta_{1}=\frac{1}{2}\arctan[ \frac{4A_{yy}+\eta\tan\left[ {2\theta}_{2} \right]}{\left( \eta+\xi\tan\left[ {2\theta}_{2} \right] \right)}]$$

If $\theta_{2}\neq\frac{\pi}{4}, \tan[2\theta_{2}]\neq\frac{\eta}{\xi}$

$$\theta_{1}=\frac{\pi}{4}$$

If $\theta_{2}\neq\frac{\pi}{4}, \tan\left[ 2\theta_{2} \right]=\frac{\eta}{\xi}, \xi\neq0$

$$\theta_{1}=\frac{1}{2}\arctan\left[ \frac{\eta}{\xi} \right]$$

If $\theta_{2}=\frac{\pi}{4}$

Note that this describes a 1-dimensional continuous curve. When $\theta_{2}\neq\frac{\pi}{4}$ and ${Tan[2\theta}_{2}]\to\frac{\eta}{\xi}$, then $\theta_{1}=\frac{1}{2}ArcTan\left[ \frac{4A_{yy}+\eta\tan\left[ {2\theta}_{2} \right]}{\left( \eta+\xi\tan\left[ {2\theta}_{2} \right] \right)} \right]\to\frac{1}{2}ArcTan\left[ \infty\right]=\frac{\pi}{4}$

which is the solution when $\theta_{2}\neq\frac{\pi}{4}, {Tan[2\theta}_{2}]=\frac{\eta}{\xi}$.

When $\theta_{2}\to\frac{\pi}{4}$, then the solution when $\theta_{2}\neq\frac{\pi}{4}$, $\theta_{1}=\frac{1}{2}\arctan[ \frac{4A_{yy}+\eta\tan\left[ {2\theta}_{2} \right]}{\left( \eta+\xi\tan\left[ {2\theta}_{2} \right] \right)}]$ goes to $\theta_{1}=\frac{1}{2}\arctan\left[ \frac{\eta}{\xi} \right]$ which is the solution when $\theta_{2}=\frac{\pi}{4}$.

Note that $\theta_{2}\neq\theta_{1}$ since we assume $A_{yy}\neq-1$, so when $\theta_{2}\to\frac{\pi}{4}$ it is okay to assume $\theta_{1}\neq\frac{\pi}{4}$.

To conclude, for every $\theta_{2}$, there is a single $\theta_{1}(\theta_{2})$, such that norms with the parameters $\theta_{1}(\theta_{2}),\theta_{2},\lambda_{1}^{2}\left( \theta_{1}(\theta_{2}) \right),\lambda_{2}^{2}(\theta_{2})$ generate the hyperbola. However, those parameters will represent matrices of inner-product norms only if $\lambda_{1}^{2}\left( \theta_{1}(\theta_{2}) \right)>0$ and $\lambda_{2}^{2}\left( \theta_{2} \right)>0$. Hence, if there exists $\theta_{2}$ such that $\lambda_{1}^{2}\left( \theta_{1}(\theta_{2}) \right)>0$ and $\lambda_{2}^{2}\left( \theta_{2} \right)>0$, the norms represented by $\theta_{1}(\theta_{2}),\theta_{2},\lambda_{1}^{2}\left( \theta_{1}(\theta_{2}) \right),\lambda_{2}^{2}(\theta_{2})$ will generate the hyperbola. We can find all norms that generate a given hyperbola by considering all $\theta_{2}'s$ for which $\lambda_{1}^{2}\left( \theta_{1}(\theta_{2}) \right)>0$ and $\lambda_{2}^{2}\left( \theta_{2} \right)>0$. In that case, we know that there is a 1-dimensional family of norms that generate this hyperbola. If there doesn’t exist such $\theta_{2}$, it means that there doesn’t exist a pair of norms that generates the given hyperbola.

We can examine the existence of such $\theta_{2}$ numerically: we plot $\lambda_{1}^{2}(\theta_{1}\left( \theta_{2} \right))$ and $\lambda_{2}^{2}(\theta_{2})$, and check if there is an area where both $\lambda_{1}^{2}$ and $\lambda_{2}^{2}$ are positive. An example for such a plot is given in Fig S3.

To conclude, we showed that we cannot uniquely determine norm pairs that generated a given front. Some hyperbolae cannot be described as the Pareto front of 2 tasks in 2D. For those who can be described, there is a one-dimensional family of norm-pairs that generate them. However, it is generally enough to determine a single parameter of one of the norms, to completely determine both norms. If such a parameter could be obtained by other means (e.g. – a biomechanical model, etc.), the above method can be used to exactly determine the norms, and therefore the relative importance of each trait to the performance.


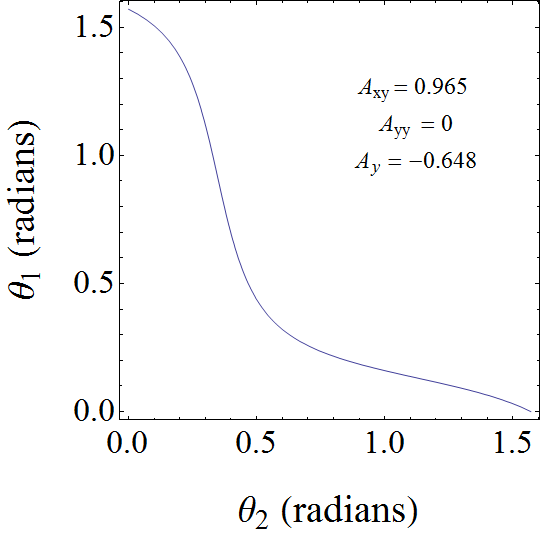


Fig S2: When considering the set of norm-pairs parameters that generate a given hyperbola, $\boldsymbol{\theta}_{\boldsymbol{1}}$ is a function of $\boldsymbol{\theta}_{\boldsymbol{2}}$. Here, an example is shown for the hyperbola with parameters as displayed in the figure.


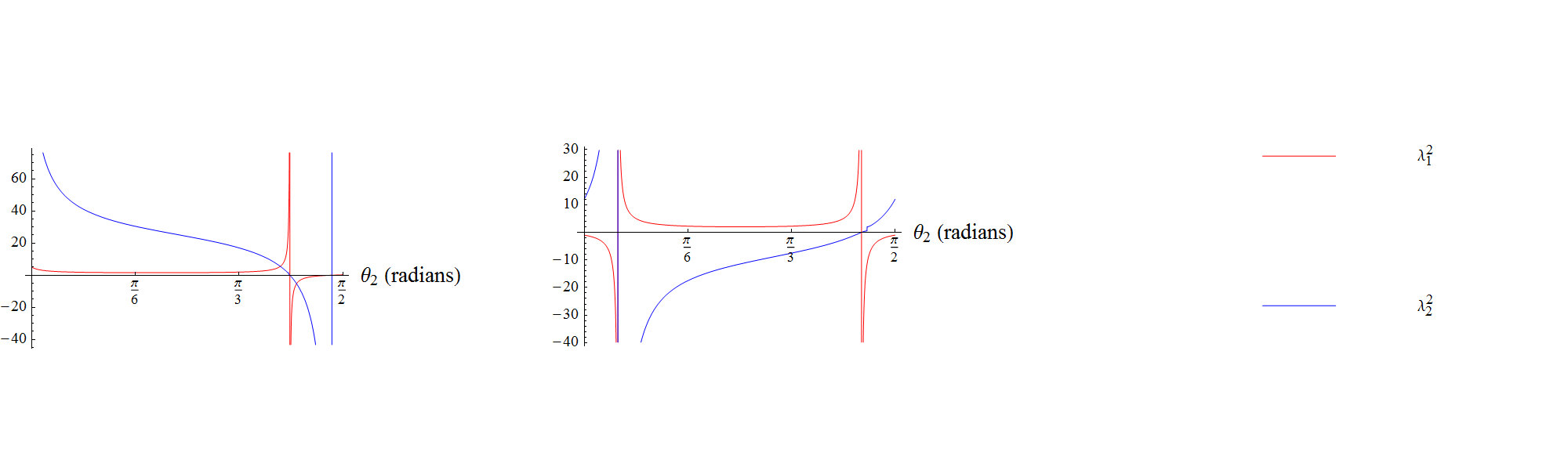


Fig S3: $\boldsymbol{\lambda}_{\boldsymbol{1}}^{\boldsymbol{2}}\boldsymbol{(}\boldsymbol{\theta}_{\boldsymbol{1}}\left( \boldsymbol{\theta}_{\boldsymbol{2}} \right)\boldsymbol{)}$ and $\boldsymbol{\lambda}_{\boldsymbol{2}}^{\boldsymbol{2}}\boldsymbol{(}\boldsymbol{\theta}_{\boldsymbol{2}}\boldsymbol{)}$, for 2 different hyperbolae. a) A plot of $\boldsymbol{\lambda}_{\boldsymbol{1}}^{\boldsymbol{2}}\boldsymbol{(}\boldsymbol{\theta}_{\boldsymbol{1}}\left( \boldsymbol{\theta}_{\boldsymbol{2}} \right)\boldsymbol{)}$ and $\boldsymbol{\lambda}_{\boldsymbol{2}}^{\boldsymbol{2}}\boldsymbol{(}\boldsymbol{\theta}_{\boldsymbol{2}}\boldsymbol{)}$ for a hyperbola with the parameters: $\boldsymbol{A}_{\boldsymbol{xy}}\boldsymbol{= -9, A}{}_{\boldsymbol{yy}}\boldsymbol{= -5,}\boldsymbol{A}_{\boldsymbol{y}}\boldsymbol{= -}\frac{\boldsymbol{1}}{\boldsymbol{2}}$. It can be seen that there exist $\boldsymbol{\theta}_{\boldsymbol{2}}\boldsymbol{'s}$ for which both $\boldsymbol{\lambda}_{\boldsymbol{1}}^{\boldsymbol{2}}\boldsymbol{(}\boldsymbol{\theta}_{\boldsymbol{1}}\left( \boldsymbol{\theta}_{\boldsymbol{2}} \right)\boldsymbol{)}$ and $\boldsymbol{\lambda}_{\boldsymbol{2}}^{\boldsymbol{2}}\boldsymbol{(}\boldsymbol{\theta}_{\boldsymbol{2}}\boldsymbol{)}$ are positive. This means that there are norms whose Pareto front is the above hyperbola. b) A plot of $\boldsymbol{\lambda}_{\boldsymbol{1}}^{\boldsymbol{2}}\boldsymbol{(}\boldsymbol{\theta}_{\boldsymbol{1}}\left( \boldsymbol{\theta}_{\boldsymbol{2}} \right)\boldsymbol{)}$ and $\boldsymbol{\lambda}_{\boldsymbol{2}}^{\boldsymbol{2}}\boldsymbol{(}\boldsymbol{\theta}_{\boldsymbol{2}}\boldsymbol{)}$ for a hyperbola with the parameters: $\boldsymbol{A}_{\boldsymbol{xy}}\boldsymbol{= 3, A}{}_{\boldsymbol{yy}}\boldsymbol{= 1,}\boldsymbol{A}_{\boldsymbol{y}}\boldsymbol{= -}\frac{\boldsymbol{1}}{\boldsymbol{3}}$. It can be seen that there isn’t any $\boldsymbol{\theta}_{\boldsymbol{2}}$ such that both $\boldsymbol{\lambda}_{\boldsymbol{1}}^{\boldsymbol{2}}\boldsymbol{(}\boldsymbol{\theta}_{\boldsymbol{1}}\left( \boldsymbol{\theta}_{\boldsymbol{2}} \right)\boldsymbol{)}$ and $\boldsymbol{\lambda}_{\boldsymbol{2}}^{\boldsymbol{2}}\boldsymbol{(}\boldsymbol{\theta}_{\boldsymbol{2}}\boldsymbol{)}$ are positive. This means that this hyperbola cannot be described as the Pareto front of any norm.

# Appendix 6

## Generally, for 3 tasks in a 2D morphospace, the norms can be uniquely determined by the shape of the Pareto front

Let us assume that we have 3 tasks in a $2D$-morphospace. The matrices that describe the norms that the performance functions depend on are denoted by $M_{i},$ for $i\in\{1,2,3\}$. Denote the parameters that $M_{i}$ depends on with $\lambda_{i}, \theta_{i}$.

The Pareto front in this case is given by (Appendix 1):

$$v=({\sum_{i=1}^{3} \alpha_{i}M_{i})}^{-1}\left( \sum_{i=1}^{3} \alpha_{i}M_{i}v_{i}^{*} \right) s.t. 0\leq\alpha_{i}\leq1, \sum_{i=1}^{3} \alpha_{i}=1$$

As proven in Appendix 7, the boundary of $P_{1,2,3}$, the 3-tasks Pareto front, is composed of the three 2-tasks fronts. Hence, given a dataset, we can take its boundary and assume that it represents 3 hyperbolae. Denote by $P_{i,j}$ the 2-tasks front between $v_{i}^{*}$ and $v_{j}^{*}$. Since there are 3 tasks, each one is associated with two 2-tasks fronts. From the front $P_{i,j}$ we can deduce the 1-dimensional family of norm-pairs that generates it, as explained in Appendix 5. Denote this family by $\mathcal{F}_{i,j}$. Denote by $\mathcal{F}_{i,j}^{i}$ the family of norms that are associated with task $i$ and were calculated from the front $P_{i,j}$. Thus, there are 2 families of potential norms that task $i$ might depend on - $\mathcal{F}_{i, j}^{i}$ and $\mathcal{F}_{i, k}^{i}$ ($i\neq j\neq k)$. However, each task can depend only on one norm. It means that if the hyperbolae triad is indeed generated by a norms-triad, then there must be at least one common member between $\mathcal{F}_{i,j}^{i}$ and $\mathcal{F}_{i,k}^{i}$. Denote $\mathcal{F}^{i}=\mathcal{F}_{i, j}^{i}\cap\mathcal{F}_{i, k}^{i}$, then $\mathcal{F}^{i}\neq\phi$. Choosing a member $n_{i}\in\mathcal{F}^{i}$ determines (see Appendix 5) a member $n_{i, j}$ of $\mathcal{F}_{i,j}$ and a member $n_{i,k}$ of $\mathcal{F}_{i,k}$. Those in turn determine a member $n_{j}\in\mathcal{F}^{j}$ and $n_{k}\in\mathcal{F}^{k}$. Define $N_{j,k}=\left\{ (n_{j},n_{k}) \right|n_{i}\in\mathcal{F}^{i}\}$. $N_{j,k}$ includes all pairs of norms on which the performance of task $j$ and task $k$ can depend, deduced from looking on task $i$. On the other hand, task *j* and task *k* generate $f_{j,k}$. This means that the existence of a triplet of norms that generate the Pareto front requires that $N_{j,k} \cap f_{j,k}\neq\phi$.

Hence, three cases are possible:

1. $|N_{j,k} \cap\mathcal{F}_{j,k}|=1$. In that case, there is only one triplet of norms that generates the given Pareto front.
2. . In that case, the norms-triplet cannot be determined uniquely from the front.
3. $\left| N_{j,k} \cap\mathcal{F}_{j,k} \right|=0$. In that case, the hyperbole-bound triangular shape corresponds to no triplet of norms and hence cannot be explained in the scope of the current model.

Let’s check under what conditions each of the above cases occur. But first, we prove the following lemma:

Lemma 1: Let $P_{i, j}$ be the Pareto front associated with tasks $i$ and $j$*.*

If one of the following occurs:

1. $I\in\mathcal{F}_{i, j}^{i}$ or $I\in\mathcal{F}_{i, j}^{j}$ (where $I$ is identity matrix, representing the Euclidean norm)
2. $\exists\left( m_{1},m_{2} \right)\in\mathcal{F}_{i,j}$ such that $\theta_{1}=\theta_{2}$.

Then for every $\lambda>0$ there is a norm-pair that generate $P_{i, j}$ given by

$$m_{1}=R\left[ \theta_{i, j} \right]\left( \begin{matrix} 1 & 0 \\ 0 & \lambda^{2} \end{matrix} \right)R[-\theta_{i, j}]$$

$$m_{2}=R\left[ \theta_{i, j} \right]\left( \begin{matrix} 1 & 0 \\ 0 & {C_{i,j} \lambda}^{2} \end{matrix} \right)R[-\theta_{i, j}]$$

where $C_{i,j}$ > 0 and $\theta_{i, j}$ are constant determined by the parameters of $P_{i,j}$.

Proof: We transform to a coordinate system where $v_{i}^{*}\mapsto\tilde{v_{i}^{*}}=\left( 0,0 \right), v_{j}^{*}\mapsto\tilde{v_{j}^{*}}=(1,0)$. This transformation is a combination of a translation, a rotation by an angle $\tilde{\theta}$, and an isomorphic scaling. Under those transformations, the parameters of the norms transform in the following way:

$$\lambda_{1}\mapsto\tilde{\lambda}_{1}=\lambda_{1}, \lambda_{2}\mapsto\tilde{\lambda}_{2}=\lambda_{2}, \theta_{1}\mapsto\tilde{\theta}_{1}=\theta_{1}+\tilde{\theta}, {\theta_{2}\mapsto\tilde{\theta}}_{2}=\theta_{2}+\tilde{\theta}$$

It means that if condition (I) or (II) holds in the original coordinate system, it will hold in the transformed coordinate system. So either way, from Appendix 5 we know that in the transformed coordinate system, $P_{i, j}\mapsto\tilde{P_{i, j}}$ whose parameter $A_{yy}=-1$. In that scenario, the family of norm pairs that generate $\tilde{P_{i, j}}$, $\tilde{\mathcal{F}_{i,j}}$, was fully classified in Appendix 5:

$\tilde{\mathcal{F}_{i,j}}= {(\tilde{m}}_{1},\tilde{m}_{2})$ $s.t.$ $\tilde{\theta}_{1}=\tilde{\theta}_{2}=\tilde{\theta_{i,j}}$, $\tilde{\lambda_{2}^{2}}= C_{i,j}\tilde{\lambda_{1}^{2}}$, for $\tilde{\theta_{i,j}}$, $\tilde{C_{i,j}}>0$ that are determined by the parameters of the hyperbola $\tilde{P_{i,j}}$. Going back to the original coordinate system, we can deduce that $\mathcal{F}_{i, j}=(m_{1},m_{2})$ $s.t\boldsymbol{.}$ $\theta_{1}=\theta_{2}=\theta_{i,j}$, $\lambda_{2}^{2}= C_{i,j}\lambda_{1}^{2}$, for $\theta_{i,j}=\tilde{\theta_{i,j}}-\tilde{\theta}$, $C_{i,j}= \tilde{C_{i,j}}$.

Consider a hyperbole-bound triangular shape with vertices $v_{1}^{*}, v_{2}^{*},v_{3}^{*}$. Denote $\mathcal{F}_{i, j}$ as above. For now we assume that there is a triplet of norms – $M_{1}, M_{2},M_{3}$- that generate that shape, i.e. $\mathcal{F}_{i,j}\cap N_{i,j}\neq\Phi$. We will attempt to characterize when this norm-triplet is unique, and show that if it is not unique, there exists a 1-dimensional family of norm-triplets that generate the same $P_{1,2,3}$.

We choose coordinate system such that $M_{1}=I$. This is possible as shown in Appendix 2. In that case, from lemma 1 we know that $\mathcal{F}_{1,2}$ - the family of norm pairs that generate $P_{1,2}$, contains norms for which $\theta_{1}=\theta_{2}=:\theta_{1,2}$ and $\frac{\lambda_{2}^{2}}{\lambda_{1}^{2}}=:C_{1,2}$, where $C_{1,2}>0{, \theta}_{1,2}$ are defined by the parameters of $P_{1,2}$. Lemma 1 also tell us that $\mathcal{F}_{1,3}$- the family of norm pairs that generate $P_{1,3}$- contains norms for which $\theta_{1}=\theta_{3}=:\theta_{1,3}$ and $\frac{\lambda_{3}^{2}}{\lambda_{1}^{2}}=:C_{1,3}$, where $C_{1,3}>0{, \theta}_{1,3}$ are defined by the parameters of $P_{1,3}$.

There are 2 cases, either $\theta_{1,3}\neq\theta_{1,2}$ or $\theta_{1,3}= \theta_{1,2}$.

Claim 1: If $\theta_{1,3}\neq\theta_{1,2}$, then $\mathcal{F}^{1}=\left\{ M_{1} \right\}=\{I\}$, and the norm-triplet is unique.

Proof: Let $M^{'}\in\mathcal{F}^{1}\Rightarrow M^{'}\in\mathcal{F}_{1,2}^{1}$ and $M^{'}\in\mathcal{F}_{1,3}^{1}$. From the above conclusions on $\mathcal{F}_{1,2}$ and $\mathcal{F}_{1,3}$ we deduce that on one hand $\theta_{M^{'}}=\theta_{1,3}$ and on the other hand $\theta_{M^{'}}=\theta_{1,2}$. If $\theta_{1,3}\neq\theta_{1,2}$, the only possibility to resolve the conflict is if $\lambda_{M^{'}}=1$ and then $M^{'}=I$, since in this case the norm is Euclidean and $\theta$ is meaningless. $M_{1}$ is thus determined uniquely, and since $M_{2}$ is determined uniquely by $M_{1}$ and also $M_{3}$ is determined uniquely by $M_{1}$. This means that there is only one triplet of norms that generates the Pareto front $\Rightarrow$ we can uniquely deduce the norms that he performance functions depend on from the Pareto front.

Claim 2: If $\theta_{1,3}= \theta_{1,2}$, then there’s a 1-dimensional family of norm-triplets that generates the given $P_{1,2,3}$.

Proof: In that case $\mathcal{F}_{1,2}^{1}=\mathcal{F}_{1,3}^{1}$. This is true since according to lemma 1 $\mathcal{F}_{1,2}^{1}$ contains all norms with $\theta= \theta_{1,2}$ and any $\lambda>0$, and $\mathcal{F}_{1,3}^{1}$ contains all norms with $\theta= \theta_{1,3}$ and any $\lambda>0$. Since $\mathcal{F}^{1}=\mathcal{F}_{1,2}^{1}\cap\mathcal{F}_{1,3}^{1}$ we get that $\mathcal{F}^{1}=\mathcal{F}_{1,2}^{1}=\mathcal{F}_{1,3}^{1}$. $\mathcal{F}_{1,2}^{1}$ is infinite since $\mathcal{F}_{1,2}$ is infinite (Appendix 5) and hence $\mathcal{F}^{1}$ is infinite. Under the current assumptions, there is $M_{1}\in\mathcal{F}^{1}$ with $\left( M_{2},M_{3} \right)\in\mathcal{F}_{2,3}$ (i.e. the norm $M_{2}$ that is paired with $M_{1}$ in $\mathcal{F}_{1,2}$ and the norm $M_{3}$ that is paired with $M_{1}$ in $\mathcal{F}_{1,3}$ generate $P_{2,3})$. Note that $M_{2}$ and $M_{3}$ both have the same angle $\theta$. In that case, from lemma 1 we get that $\left( m_{2},m_{3} \right)\in f_{2,3}$ $\Leftrightarrow\theta_{2}=\theta_{3}=\theta_{2,3} and \lambda_{3}^{2}=C_{2,3}\lambda_{2}^{2}$, for $\theta_{2,3}$ and $C_{2,3}$ determined by the parameters of the hyperbola.

$\left( M_{2},M_{3} \right)$ generates $P_{2,3}$, and thus $\frac{\lambda_{M_{2}}^{2}}{\lambda_{M_{3}}^{2}}=C_{2,3}$ and $\theta_{M_{2}}= \theta_{M_{3}}=\theta_{2,3}$. In addition, $\left( M_{1},M_{2} \right)\in\mathcal{F}_{1,2}\Rightarrow\lambda_{M_{2}}^{2}=C_{1,2} \lambda_{M_{1}}^{2}.$

$\left( M_{1},M_{3} \right)\in\mathcal{F}_{1,3}\Rightarrow\lambda_{M_{3}}^{2}=C_{1,3} \lambda_{M_{1}}^{2}$.

$\Rightarrow C_{2,3}= \frac{\lambda_{M_{3}}^{2}}{\lambda_{M_{2}}^{2}}=\frac{C_{1,3} \lambda_{M_{1}}^{2}}{C_{1,2} \lambda_{M_{1}}^{2}} =\frac{C_{1,3}}{C_{1,2}}$, and $\theta_{M_{1}}= \theta_{M_{2}}=\theta_{1,2}=\theta_{1,3}= \theta_{M_{3}}= \theta_{2,3}$.

Now take another $M_{1}^{'}\in\mathcal{F}^{1}$. We know that, $\lambda_{M_{2}^{'}}^{2}=C_{1,2} \lambda_{M_{1}^{'}}^{2} , \lambda_{M_{3}^{'}}^{2}=C_{1,3} \lambda_{M_{1}^{'}}^{2}\Rightarrow\frac{\lambda_{M_{3}^{'}}^{2}}{\lambda_{M_{2}^{'}}^{2}}=\frac{C_{1,3} \lambda_{M_{1}^{'}}^{2}}{C_{1,2} \lambda_{M_{1}^{'}}^{2}}= \frac{C_{1,3}}{C_{1,2}} =C_{2,3}$. We also know that $\theta_{M_{1}^{'}}= \theta_{M_{2}^{'}},$ $\theta_{M_{1}^{'}}= \theta_{M_{3}^{'}}$ $\Rightarrow\theta_{M_{2}^{'}}= \theta_{M_{3}^{'}}\Rightarrow$ $\left( M_{2}^{'},M_{3}^{'} \right)\in\mathcal{F}_{2,3}$. Hence, every $M_{1}^{'}\in\mathcal{F}^{1}$ is a part of a norm-triplet that generates the hyperbolae triplet. Since $\mathcal{F}^{1}$ is infinite, it means that there are infinite number of norm-triplets that generate the given hyperbolae-bound shape. The family is defined by one of the $\lambda$ parameters, since all $\theta$s are known, and every other $\lambda$ is determined by the respective $C$ constant.

Those conclusions are true in the frame where $M_{1}=I$ – i.e. – there is a norm triplet that generates the Pareto front in which the norm associated with task 1 is Euclidean. We can go back to the original coordinate system by rotating, translating and rescaling the space. Those transformations are invertible, so there is a 1-to-1 correspondence between norm-triplets that generates the shape in the transformed coordinate system and norm-triplets that generates the shape in the original coordinate system.

It can be seen that most of hyperbolae-bound triangular shapes that correspond to norms triplets correspond to a single triplet, since it is much more common that $\theta_{1}\neq\theta_{2}$.

This method can be used to find the norms that generate a given hyperbolae-bound triangular shape. We can fit $P_{1,2}$ and $P_{1,3}$, and find $\mathcal{F}_{1,2}$ and $\mathcal{F}_{1,3}$. Then, we find $\mathcal{F}_{1,2}^{1}$,$\mathcal{F}_{1,3}^{1}$ and $\mathcal{F}^{1}=\mathcal{F}_{1,2}^{1}\cap\mathcal{F}_{1,3}^{1}$. If $\mathcal{F}^{1}=\phi$ we deduce that the shape corresponds to no norms-triplet. Otherwise, we choose ${M_{1}\mathcal{\in F}}^{1}$. We change coordinate system such that $M_{1}=I$ while $v_{1}^{*}, v_{2}^{*}$ are at $\left( 0,0 \right), (1,0)$, respectively. From the transformed $f_{1,2}$ and $f_{1,3}$, we find $\theta_{1,2}$ and $\theta_{1,3}$. Examining if $\theta_{1,2}= \theta_{1,3}$ determines if the solution is unique or degenerate.

# Appendix 7

## The boundary of the 3-tasks Pareto front is composed of the three 2-tasks Pareto fronts

In this Appendix, we will show that the boundary of the Pareto front associated with 3 tasks in a 2D morphospace is composed of the 3 Pareto fronts associated with each pair of tasks

Consider the Pareto front defined by tasks , denoted by $P_{i, j}$. As seen before - this front is a section of a hyperbola (or a line as a special case).

Denote the hyperbola branch that contains $P_{i, j}$ by $B_{i, j}$ and the entire hyperbola by $H_{i, j}$ (in case $P_{i, j}$ is a line, $B_{i, j}=H_{i, j})$. The hyperbola branch divides the space into 3 parts – one side of $B_{i, j}$, $B_{i, j}$ itself and the other side of $B_{i, j}$.

Denote by $P_{1,2,3}$ the Pareto front associated with all three tasks.

First note that $\forall i,j: P_{i,j}\subseteq P_{1,2,3}$: If $v\in P_{i,j}$, then for every $v^{'}\neq v$, there is $k\in\{i,j\}$ such that $P_{k}\left( v^{'} \right)<P_{k}\left( v \right)$, and specifically there is $k\in\{1,2,3\}$ such that $P_{k}\left( v^{'} \right)<P_{k}\left( v \right)$ $\Rightarrow$ $v\in P_{1,2,3}$ $\Rightarrow$ $P_{i,j}\subset P_{1,2,3}$.

For convenience sake, and without loss of generality, we assume that the gradient $g_{i}=\nabla P_{i}$ is normalized ($g_{i}\neq0$ except at the archetype $v_{i}^{*}$, everywhere else we can redefine $g_{i}:=\frac{g_{i}}{\left\| g_{i} \right\|}$).

For now, assume that for $i, j, k\in\{1,2,3\}$ such that $i\neq j\neq k$, $H_{i, j}\neq H_{i, k}$. The case where there are overlapping hyperbolae will be discussed later.

We will implicitly consider the 2-dimensional morphospace V as embedded in a 3-dimensional vector space in the trivial way ($\left( x, y \right)\mapsto\left( x,y,0 \right)$) for the use of operations such as cross product. So expressions such as $g_{i}\times g_{j}$ should be understood as operations in the 3-dimensional space, while operations such as $\partial P$ should be understood as operations in the original 2-dimensional space.

Consider the functional $f_{i, j}=\pi_{z}\circ(g_{i}\times g_{j})$, where $\pi_{z}$ is the standard projection function $\pi_{z}\left( x, y, z \right)=z$. $g_{i}\times g_{j}=(0,0,\left\| g_{i} \right\|\left\| g_{j} \right\|\sin\left[ \theta_{i, j} \right])$, where $\theta_{i, j}$ is the angle between $g_{i}$ and $g_{j}$, measured anticlockwise from $g_{i}$ to $g_{j}$, so we get

$$f\left( v \right)=\left\| g_{i}(v) \right\|\left\| g_{j}(v) \right\|\sin\left[ \theta_{i, j} \right]$$

Lemma 1: On $B_{i, j}$, $f_{i,j}\equiv0$. On one side of $B_{i, j}$, close enough to it, $f_{i,j}>0$, and on the other side of $B_{i, j}$, close enough to it, $f_{i,j}<0$.

Proof: First note that $f$ is continuous as a projection of a cross product of 2 continuous functions.

As we demonstrated before – on the Pareto front $P_{i, j}$, the gradients point in opposite directions, meaning $\sin\left[ \theta_{i, j} \right]= 0$, which implies $f_{i, j}=0$.

We’ve also seen that $P_{i, j}$ is part of a hyperbola (or line) $H_{i, j}$. $H_{i, j}$ is given by a quadratic (or linear) form $H$ such that $H_{i, j}=\left\{ v \right|H\left( v \right)=0\}$

$f_{i,j}$ is also a quadratic (or linear) form (as a cross product of two linear forms), and $\left. f_{i,j} \right|_{P_{i,j}}\equiv\left. H \right|_{P_{i, j}}\equiv0$, which implies $f=\alpha H$ for some $\alpha\mathbb{\in R}$.

In case $P_{i, j}$ is a line – $f_{i,j}$ is a linear functional and it is trivial that it is negative on one half space, and positive on the other.

In the case where $P_{i, j}$ is a hyperbola – by definition $f_{i,j}=\alpha H=0$ on the hyperbolae (both branches). We’ll show that it changes sign between the 3 connected components of $\mathbb{R}^{2}\setminus H_{i, j}$ - let *h* be the line between the 2 foci of the hyperbola. *h* intersects each branch of the hyperbola exactly once. Consider the function $f_{i,j}\circ h$ - it is a quadratic real function of a single parameter (since $f_{i,j}\mathbb{: R\to}\mathbb{R}^{2}$ is quadratic in 2 variables and $h\mathbb{: R\to R}$ is linear). $f_{i,j}\circ h=0$ on both intersections of $h$ with $H_{i,j}$. It means that $f|_{h}$ has to change sign once it passes the hyperbola. So on one side of a branch of the hyperbola $f_{i,j}|_{h}$ is positive and on the other side it is negative.

Of course $f_{i,j}$ is continuous, and $f_{i,j}\neq0$ on $\mathbb{R}^{2}\setminus H_{i, j}$, so its sign is constant across the connected components. Hence, on one side of $B_{i, j}$, $f_{i,j}$ is positive, and on the other side $f_{i,j}$ is negative (as long as the other branch of the hyperbola is not approached).

All in all, in a neighborhood of $B_{i, j}$ (and hence of $P_{i, j}$), $f_{i,j}$ is positive on one side of $B_{i, j}$, negative on the other side of $B_{i, j}$, and 0 on $B_{i, j}$.

$f_{i,j}>0$ implies that $g_{j}$ is less than $\pi$ radians anticlockwise than $g_{i}$, $f_{i,j}<0$ implies that $g_{j}$ is more than $\pi$ radians anticlockwise than $g_{i}$. We would like to show that $P_{i, j}$ is at the boundary of $P_{1,2,3}$, the Pareto front associated with all 3 tasks. To do so, we need to show that

$$\forall v\in P_{i, j} and \forall\epsilon>0: \exists{v^{'}\in B_{0}\left( v,\epsilon\right) s.t. v}^{'}\in\mathbb{R}^{2}\backslash P_{1,2,3}$$

where $B_{0}(v,\epsilon)$ is an open ball of radius $\epsilon$ around $v$.

Theorem 2: $\forall i, j (i\neq j): P_{i, j}\subseteq\partial P_{1,2,3}$

Proof: Assume without loss of generality that $i=1, j=2$ (the proof will be identical for any pair of $i, j$ as long as $i\neq j$). Assume by negation $P_{1,2}$ is not at the boundary of $P_{1,2,3}$, it means that there exist $v\in P_{1,2}$ and $\epsilon>0$ such that $B_{0}\left( v, \epsilon\right)\subset P_{1,2,3}$.

Let $\epsilon>0$, and $u\in B_{0}(v, \epsilon)$.

Claim 2: For $u\in P_{1,2,3}$, $f_{i, j}(u) (j\neq i)$ and $f_{i, k}(u) (k\neq i, j)$ can’t be neither both positive nor both negative.

Proof:

Assume $f_{i, j}>0\Rightarrow g_{j}$ is less than $\pi$ radians anticlockwise to $g_{i}$.

In this case $f_{i, k}$ can’t be positive: If $f_{i, k}>0$, it means that $g_{k}$ is less than $\pi$ radians anticlockwise to $g_{i}$. In this case all three gradients lie in the same half-space -choose the gradient $g_{m}$ that has the maximal angle with $g_{i}$ ($m=argmax_{m}\{f_{i, m}\}$). The angle between $g_{i}$ and $g_{m}$ is smaller than $\pi$ (because both gradients are less than $\pi$ radians anticlockwise from $g_{i}$. Choose $h_{u}$ to be the unit vector bisecting the angle between $g_{i}$ and $g_{m}$ ($h_{u}=\frac{\hat{g}_{i}+\hat{g}_{m}}{2}$). The angle between $h_{u}$ and each gradient is smaller than $\frac{\pi}{2}$. Then, ${\forall n: h}_{u}\cdot g_{n}>0$. However, we showed in Appendix 1 that $u$ is Pareto optimal if and only if there doesn’t exists a vector $h_{u}$ such that $\forall n: h_{u}\cdot g_{n}>0$. Since $u\in P_{1, 2, 3}$ (e.g. is Pareto optimal), we must conclude that $f_{i, k}≯0$.

To show that $f_{i, j},$ and $f_{i, k}$ can’t both be negative, follow the above proof while changing the word “anticlockwise” to “clockwise”.

Corollary 1: For $u\in P_{1,2,3}$, such that $\forall i, j: u\notin H_{i, j}$- $sgn\left( f_{1,2}\left( u \right) \right)=sgn\left( f_{2,3}\left( u \right) \right)=sgn\left( f_{3,1}\left( u \right) \right).$

Proof: This results directly from the claim, the anti-symmetry of $f_{i,j}$, and the fact that $f_{i,j}\left( u \right)=0\Leftrightarrow u\in H_{i,j}$

Claim 3: If $u\notin P_{1,2,3}$ and $u\notin H_{i, j}$ for any $i, j$, not all $f_{1,2}\left( u \right),f_{2,3}\left( u \right),f_{3,1}(u)$ have the same sign (see Figure S4)

Proof: According to Conclusion 1 from Appendix 1, since $u$ is not Pareto optimal, all three gradients $g_{1},g_{2},g_{3}$ lie in the same half-space. Choose a vector $l$ on the line separating the two half-spaces such that $\pi_{z}(l\times g_{i})$ are all positive (they are either all positive or all negative since they are all in the same half-space). If we order the gradients according to their anticlockwise angle from $l$ (they are all smaller than $\pi)$ and name them $g_{i}, g_{j}, g_{k}$ we get that $f_{i, j}>0, f_{j, k}>0$ but $f_{k, i}<0$, since the anticlockwise angles from $i$ to $j,$ from $j$ to $k$ and from $i$ to $k$ are smaller than $\pi$ radians (they are all non-zero since $u$ is not on any hyperbola), and since $f$ is anti-symmetric $f_{i, k}\geq0\Rightarrow f_{k, i}\leq0$.

This proves the claim for every assignment of $i, j,$ and $k$.

Corollary 2: a point $u\in V$, which is not on any hyperbola $H_{i, j}$ is Pareto optimal if, and only if, $sgn\left( f_{1,2}\left( u \right) \right)=sgn\left( f_{2,3}\left( u \right) \right)=sgn\left( f_{3,1}\left( u \right) \right).$

Now, it is clear that if $u\in P_{i, j}$, in every neighborhood of $u$ there are points from both sides of $P_{i, j}$, meaning points with $f_{i, j}>0$ and points with $f_{i, j}<0$. This means that every point on $P_{i, j}$ that is not any other hyperbola $H_{k,l}$ (i.e. not an intersection point), if it has Pareto optimal points on one side of $P_{i, j}$, on the other side there are no Pareto optimal points (since only $f_{i, j}$ changes sign on $H_{i, j}$).

If the point $u\in P_{i, j}$ is an intersection of two or more hyperbolae $H_{i, j}$ and some $H_{k, l}$ (not necessarily on $P_{k, l}$), then in every neighborhood there are points on $P_{i, j}$ that are *not* intersection points (the number of intersection points between 2 hyperbolae/lines is finite) and therefore in that neighborhood there are points that are not Pareto optimal. Note that the archetypes, for example, are at the intersection of $P_{i, j}$ with $P_{i, k}$.

Thus – any point $u\in P_{i, j}$ for every pair of $i, j$, is not in the interior of $P_{1,2,3}$, but since it is in $P_{1,2,3}$ it must be in $\partial P_{1,2,3}$.

Theorem 3: For every $u\in P_{1,2,3}$, if $u\notin P_{i, j}$ for any $i, j$, then $u\notin\partial P_{1,2,3}$

Proof 3: First, we will show that $u$ is not on any hyperbola. If it were on some $H_{i, j}$, then $g_{i}\left( u \right)\times g_{j}\left( u \right)=0$. But since $u\notin P_{i, j}$ then the gradients $g_{i}$ and $g_{j}$ cannot point to opposite directions, so they must point in the same direction. i.e. $g_{i}+\alpha g_{j}=0$ but $\alpha<0$. Since $u$ is Pareto optimal, then the third gradient $g_{k}$ ($k\neq i, j)$ must point in the opposite direction (otherwise there was $h$ s.t. $\forall n: h\cdot g_{n}>0$, namely $h=\frac{g_{i}+g_{k}}{2}$). So we get that $g_{i}$ and $g_{k}$ must point in opposite directions, which means that $u\in P_{i, k}$, which is in contradiction to the assumption.

So $u$ is not on any hyperbola, and hence there is a neighborhood of $u$ that doesn’t include any point of $H_{i,j}$ for each *i,j*. If $u$ is Pareto optimal, then the signs of $f_{1,2}, f_{2,3},f_{3,1}$ are all equal on $u$, but since all $f's$ change signs only on the hyperbolae, they all have the same sign in a neighborhood of $u$, which means that there is a neighborhood of $u$ which is Pareto optimal, i.e. – $u$ is in the interior of $P_{1,2,3}$.

Theorem 4: $\partial P_{1,2,3}=P_{1,2}\cup P_{2,3}\cup P_{3,1}$

We showed that $\forall i,j:P_{i,j}\subset\partial P_{1,2,3}$, and that for every $u\in P_{1,2,3}$, such that $u\notin P_{i, j}$ for any $i, j$, then $u\notin\partial P_{1,2,3}$. All that remains to be shown is that if $u\notin P_{1,2,3}$, then $u\notin\partial P_{1,2,3}$. That is true because $P_{1,2,3}$ is a closed set. In Appendix 1 we showed that:

$$P_{1,2,3}=\left. \left\{ \left( \sum_{i} \alpha_{i}M_{i} \right)^{-1}\sum_{i} \left( \alpha_{i}M_{i}v_{i}^{*} \right) | \alpha_{i}\geq0, \sum_{i=1}^{3} \alpha_{i}=1 \right\} \right.$$

So $P_{1,2,3}$ is the image of the (compact) unit triangle ($0\leq\alpha_{1}+\alpha_{2}\leq1$) under the continuous mapping

$$\alpha_{1},\alpha_{2}\mapsto\alpha_{1},\alpha_{2},1-\alpha_{1}-\alpha_{2}\mapsto\left( \sum_{i} \alpha_{i}M_{i} \right)^{-1}\sum_{i} \left( \alpha_{i}M_{i}v_{i}^{*} \right)$$

(It is continuous since $\sum_{i} \alpha_{i}M_{i}$ is a non negative combination of positive definite matrices, and not all coefficients are zero, and hence it is positive definite, and hence always invertible).

The image of compact sets are compact, and specifically closed, so there cannot be any points outside $P_{1,2,3}$ in $\partial P_{1,2,3}$.

Lemma: For every $i\neq j\neq k$, $H_{i,j}$ and $H_{k,i}$ can intersect only once except in the archetype $A_{i}$.

Proof: First note that $H_{j,k}$ goes through any intersection point between $H_{i,j}$ and $H_{k,i}$. Let $t$ be an intersection point between $H_{i,j}$ and $H_{k,i}$. $t\in H_{i,j}$. As shown earlier this implies that $g_{i}\left( t \right)\parallel g_{j}\left( t \right)$. $t\in H_{k,i}\Rightarrow g_{i}\left( t \right)\parallel g_{k}\left( t \right)$. From the above two conclusions we can conclude that $g_{j}\left( t \right)\parallel g_{k}\left( t \right)\Rightarrow t$ is on $H_{j,k}$. We see that except the archetypes, each intersection point between 2 of the hyperbolae is actually an intersection point between all 3 hyperbolae. Two different hyperbolae can intersect only 4 times. As each pair of hyperbolae intersect at an archetypes, it means that excluding the archetypes, there are at most 3 intersection points between the hyperbolae, and specifically a total of at most 3 intersection points between any $P_{i,j}$ and any $P_{k,j}$.

We showed that $\partial P_{1,2,3}=P_{1,2}\cup P_{2,3}\cup P_{3,1}$. We now want to show that there are points $v\in P_{1,2,3}$ such that $v\notin P_{i,j}$ for any $\left\{ i,j \right\}\in\{1,2,3\}$ (i.e. – there are Pareto optimal points beside the 3 2-tasks Pareto fronts).

Lemma: Let $v\in P_{i,j}$ such that $v$ is not an intersection point between $H_{i,j}$ and any other hyperbola $H_{i,k}$. Then, if $v=\left( \sum_{m} \alpha_{m}M_{m} \right)^{-1}\sum_{m} \left( \alpha_{m}M_{m}v_{m}^{*} \right)$ for $\alpha_{m}\geq0, \sum_{i=1}^{3} \alpha_{i}=1$, then $\alpha_{k}=0$, for $k\in\left\{ 1,2,3 \right\}, k\neq i,j$.

Proof: $v= \left( \sum_{m} \alpha_{m}M_{m} \right)^{-1}\sum_{m} \left( \alpha_{m}M_{m}v_{m}^{*} \right)\Rightarrow\sum_{m} \alpha_{m}M_{m}{(v-v}_{m}^{*})=0$. However, we showed that we can assume that $P_{m}\left( v \right)= -\left( v-v_{m}^{*} \right)^{T}M_{m}(v-v_{m}^{*})$. Hence, $\sum_{m} \alpha_{m}g_{m}(v)=0$. $v\in P_{i,j}$, so $g_{i}||g_{j}\Rightarrow{\alpha_{i}g}_{i}+\alpha_{j}g_{j}=c g_{i}\Rightarrow c g_{i}+\alpha_{k}g_{k}=0$ ($k\in\left\{ 1,2,3 \right\}, k\neq i,j$). Since $v$ is not an intersection point between $H_{i,j}$ and any other hyperbola, $v$ is not an archetype, so $g_{i}(v)\neq0$. Also, it means that $g_{i}(v)∦g_{k}(v)$, as otherwise $v$ would be on $H_{1,3}$. It means that $c g_{i}+\alpha_{k}g_{k}=0\Leftrightarrow c=0 and \alpha_{k}=0$.

Theorem 5: Let $v\in P_{i,j}$ for any $i\neq j$. Let $U$ be an open environment of $v$. Then, there is $v^{'}\in U$ such that $v^{'}\in P_{1,2,3}/P_{1,2}\cup P_{2,3}\cup P_{3,1}$.

Proof:

As mentioned earlier, $P_{1,2,3}$ is the image of the triangle $T=\{0\leq\alpha_{1}+\alpha_{2}\leq1\}$ under the map:

$$f: \alpha_{1},\alpha_{2}\mapsto\left( \sum_{i} \alpha_{i}M_{i} \right)^{-1}\sum_{i} \left( \alpha_{i}M_{i}v_{i}^{*} \right)$$

As shown earlier, $f$ is continuous. $f\left( \left\{ \alpha_{1},\alpha_{2} \right\} \right)=v\Rightarrow{v=\left( \sum_{i} \alpha_{i}M_{i} \right)}^{-1}\sum_{i} \left( \alpha_{i}M_{i}v_{i}^{*} \right)$ where $\alpha_{3}=1-\alpha_{1}-\alpha_{2}$

Let $v\in P_{i,j}$. Either $v$ is an intersection point between $H_{i,j}$ and another hyperbola $H_{k,i}$, or it is not.

First assume that $v$ is not an intersection point between $H_{i,j}$ and any other hyperbola $H_{k,i}$. Let $U$ be an open environment of $v$ that doesn’t intersect any hyperbola besides $P_{1,2}$. The above lemma implies that for every $v^{'}$ such that $v^{'}\in P_{i,j}\cap U$, $f^{-1}(v^{'})\subseteq T$ can’t contain points for which $\alpha_{i}+\alpha_{j}\neq1$. Let $\tilde{\alpha_{i}}, \tilde{\alpha_{j}}$ be such that $\tilde{\alpha_{i}}g_{i}(v)+ \tilde{\alpha_{j}}g_{j}(v)=0$. Such $\tilde{\alpha_{i}}, \tilde{\alpha_{j}}$ exist since $v\in P_{i,j}.$

Since $f$ is continuous, the origin of $U$ is open in $T$. {$\tilde{\alpha_{i}}, \tilde{\alpha_{j}}\}\in f^{-1}\left( U \right)\Rightarrow$ $f^{-1}(U)$ is a non-empty open environment of $\{\tilde{\alpha_{i}}, \tilde{\alpha_{j}}\}$ and hence must contain points from the interior of the triangle $T$, i.e there are $\left\{ \alpha_{i}^{'},\alpha_{j}^{'} \right\}\in f^{-1}\left( U \right), \alpha_{i}^{'}+\alpha_{j}^{'}<1$ such that $f\left( \left\{ \alpha_{i}^{'},\alpha_{j}^{'} \right\} \right)\in U$. $f\left( \left\{ \alpha_{i}^{'},\alpha_{j}^{'} \right\} \right)$ can’t be on $P_{i,j}$. Otherwise, as $U$ doesn’t intersect any other hyperbola, we would have gotten by the lemma that $\alpha_{k}^{'}=0$ , or $\alpha_{i}^{'}+\alpha_{j}^{'}=1$, in contradiction to the assumption. So, $f\left( \left\{ \alpha_{1}^{'},\alpha_{2}^{'} \right\} \right)$ is not on $P_{i,j}$ but also not on any other hyperbola, as by assumption $U$ does not intersect them. However, $f\left( \left\{ \alpha_{1}^{'},\alpha_{2}^{'} \right\} \right)\in P_{1,2,3}$. So, we get that $f\left( \left\{ \alpha_{1}^{'},\alpha_{2}^{'} \right\} \right)\in P_{1,2,3}\backslash P_{1,2}\cup P_{2,3}\cup P_{3,1}$

If $v$ is an intersection point between $H_{i,j}$ and any other hyperbola $H_{k,i}$, then since the number of intersection points between the different hyperbolae is finite, there exists $u\in U\cap P_{i,j}$ that is not an intersection point between $P_{i,j}$ and any other hyperbola. Let $V\subseteq U$ be an open environment of $u$, then by the above proof there exists $v^{'}\in V\subseteq U$ such that $v^{'}\in P_{1,2,3}/(P_{1,2}\cup P_{2,3}\cup P_{3,1})$. This is the required $v'∎$

So we showed that the boundary of the 3-tasks Pareto front is exactly the 3 2-tasks Pareto fronts, but they are not identical (i.e. it is not ‘empty’). Now we would like to better characterize the 3-tasks Pareto front.

Examine ${\mathfrak{R}^{2}}/{\bigcup_{i, j} H_{i, j}}$ . It is divided into connected components, $X_{k}$.

Theorem 6: Each component $X_{k}$ is either entirely Pareto optimal or not Pareto optimal at all.

Proof: Let $x_{k}\in X_{k}$. By corollary 2, $x_{k}$ is Pareto optimal $\Leftrightarrow$ $f_{1,2}\left( x_{k} \right)=f_{2,3}\left( x_{k} \right)=f_{3,1}(x_{k})$. By lemma 1, $f_{i,j}$ changes signs only on $H_{i,j}$, which means that the sign of each $f_{i,j}$ is constant across each $X_{k}$. Hence, if $x_{k}$ is Pareto optimal, $X_{k}$ is entirely Pareto optimal, and if $x_{k}$ is not Pareto optimal, $X_{k}$ is entirely not Pareto optimal.

Theorem 7: If $X_{k}$ is unbounded it is not Pareto optimal

Proof: The Pareto front is compact as a continuous image of a compact set (the triangle $T)$, and hence it is bounded.

Theorem 8: 2 adjacent connected components, $X_{k}$ and $X_{m}$, cannot be both Pareto optimal.

Proof: Assume $X_{k}$ is Pareto optimal. It means that in this area, $f_{1,2}=f_{2,3}=f_{3,1}$.

Assume that without loss of generality that $X_{k}$ and $X_{m}$ are separated by $H_{1,2}$. It means that $f_{1,2}$ (and only $f_{1,2}$) has a different sign on $X_{k}$ and on $X_{m}$. Hence, $f_{1,2}\not\equiv f_{2,3}=f_{3,1}$ on $X_{m}$, so $X_{m}$ is not Pareto optimal.

Theorem 9: Let $u\in P_{i,j}$. Then $u$ is at the boundary of a Pareto optimal region.

Proof: Each $u$ is at the boundary of 2 connected components $X_{k}$. Theorem 5 shows that in every open environment $U$ of $u$ there are Pareto optimal points that are not on $P_{i,j}$. This implies that one of the connected components is entirely Pareto optimal. According to theorem 8, the second one is necessarily *not* Pareto optimal. This implies that $u$ is at the boundary of a Pareto optimal region.

Theorem 10: Let $u\in H_{i,j}/P_{1,2}\cup P_{2,3}\cup P_{3,1}$. Then the connected components on which boundary $u$ is, are not Pareto optimal.

Proof: $u$ cannot be Pareto optimal. Since $u\in{H_{i,j}}/{P_{i,j}}$, $g_{i}$ and $g_{j}$ point to the same direction. $g_{k}$ ($k\neq i\neq j$) must also point to the same direction (otherwise $g_{k}$ would point to the opposite direction than $g_{i}$ and $g_{j}$, implying that $u\in P_{i,k}, P_{j,k}$ in contradiction). So, all three gradients are in the same half plane. As shown in Appendix 1, this means $u$ is not Pareto optimal. Since the Pareto front is compact, it is also closed, and has an open compliment. Hence, there is an environment $U$ of $u$ which is not Pareto optimal. If $u$ is on the boundary of a connected component $X_{k}$, then $U\cap X_{k}\neq\Phi$, meaning that $X_{k}$ contains points that are not Pareto optimal, and hence $X_{k}$ is not Pareto optimal.

We showed that $\partial P_{1,2,3}=P_{1,2}\cup P_{2,3}\cup P_{3,1}$, if for every $i\neq j\neq k\in\{1,2,3\}$, $H_{i,j}\neq H_{i,k}$, yet this statement is true also if there are such $i,j,k$.

Assume that $H_{i, j}=H_{i,k}$. As shown, $H_{i, j}=\left\{ u|{f_{i, j}\left( u \right)=0\Leftrightarrow g}_{i}(u)\times g_{j}(u)=0 \right\}$, and $H_{i, k}=\left\{ u|g_{i}(u)\times g_{k}(u)=0 \right\}$. It means that on $H_{i, j}$, $g_{i}$ is parallel to $g_{j}$, and $g_{i}$ is parallel to $g_{k}\Rightarrow g_{j}$ is parallel to $g_{k}$ $\Rightarrow g_{j}\times g_{k}=0$ on $H_{i, j}$ $\Rightarrow f_{j, k}=0$ on $H_{i,j}$. As explained earlier, this implies that ${H_{j, k}=H}_{i, j}$. $H_{i,j}$ divides the space into 2 (if it’s a line) or 3 (if it’s a hyperbola) parts. Each of $f_{i, j}, f_{i, k},f_{j, k}$ changes sign when passing any branch of $H_{i, j}$. So, either they always have the same sign (expect on $H_{i, j}$ itself), or they never have the same sign. As seen before - a point outside of $H_{i, j}$ is Pareto optimal if and only if $f_{i, j}, f_{i, k}$ and $f_{j, k}$ all have the same sign. So either the entire space (maybe except parts of $H_{i, j}$) is Pareto optimal, or none of it is. However, we showed that the Pareto front is compact, and hence bounded, and hence it cannot be the entire space. So, the Pareto front is placed on $H_{i, j}$ (which has no interior), i.e. - $P_{1,2,3}=\partial P_{1,2,3}$.

To show that ${\partial P_{1,2,3}=P}_{1,2}\cup P_{2,3}\cup P_{3,1}$ we need to show that $P_{1,2,3}=P_{1,2}\cup P_{2,3}\cup P_{3,1}$. On one hand, $P_{1,2}\cup P_{2,3}\cup P_{3,1}\subseteq P_{1,2,3}$. On the other hand, $P_{1,2,3}\subseteq H_{i,j}$, so if $u\in P_{1,2,3}$, $u\in H_{i, j}$ and then $g_{1}\left( u \right),g_{2}\left( u \right),g_{3}(u)$ are either all aligned, or one is pointing away from the other two. The first case happens if and only if the point is not Pareto optimal, so for $u\in P_{1,2,3}$ the latter must happen. However, in that case $u\in P_{m, n}$ for $m, n\in\{1,2,3\}$ $\Rightarrow$ $u\in P_{1,2}\cup P_{2,3}\cup P_{3,1}\Rightarrow{P_{1,2,3}\subseteq P}_{1,2}\cup P_{2,3}\cup P_{3,1}\Rightarrow{P_{1,2,3}=P}_{1,2}\cup P_{2,3}\cup P_{3,1}.$


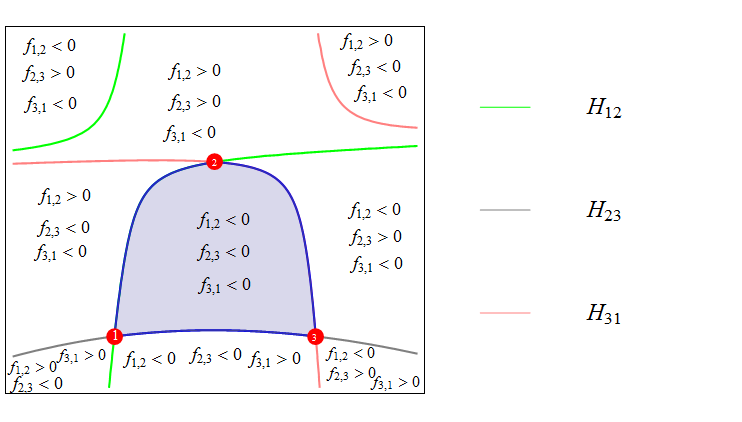


Figure S4: For 3 tasks in 2D, the Pareto front is the locus of all points such that all $\boldsymbol{f}_{\boldsymbol{i, j}}$ have the same sign. Each of the 2 tasks Pareto fronts, $\boldsymbol{P}_{\boldsymbol{i, j}}$ (in blue), lies on a hyperbola $\boldsymbol{H}_{\boldsymbol{i, j}}$. $\boldsymbol{f}_{\boldsymbol{i, j}}$ switches sign when passing $\boldsymbol{H}_{\boldsymbol{i, j}}$. $\boldsymbol{H}_{\boldsymbol{1,2}}\boldsymbol{,}\boldsymbol{H}_{\boldsymbol{2,3}}$ and $\boldsymbol{H}_{\boldsymbol{3,1}}$ are plotted in green, gray and pink, respectively. The Pareto front associated with the three tasks, $\boldsymbol{P}_{\boldsymbol{1, 2,}\boldsymbol{3}}$ is plotted in light blue.

# Appendix 8

## The resulting Pareto front when one of the performance function is maximized in a region

Consider again the case of 3 tasks in $n$ dimensions, each with a point archetype $a_{i}$, and a performance function $\tilde{P}_{i}$ that decays with an inner-product norm from the archetype. Denote by $T$ the resulting Pareto front. Now consider the case where $\tilde{P}_{1}$ is truncated – instead of a point archetype there is a region, $A_{1}$, that maximizes performance. Denote the truncated performance function by $P_{1}$. Since $P_{1}$ is truncated, it means that outside the archetypal region, its contours are identical to those of $\tilde{P}_{1}$. For convenience - denote $\tilde{P}_{2},\tilde{P}_{3}$ by $P_{2},P_{3}$.

We would now like to calculate the Pareto front relative to $P_{1}, P_{2}, P_{3}$.

Theorem: The Pareto front relative to $P_{1}, P_{2}, P_{3}$ is composed of $P_{2,3}\cup(T/{A_{1}^{^{\circ}}})$, where $P_{2,3}$ is the Pareto front related to $P_{2}$ and $P_{3}$.

Proof:

Lemma: If $x\notin T$, it is not Pareto optimal.

Proof: $x\notin T$. In Appendix 1 we saw that this means that there is $y$ in the vicinity of $x$ such that $\forall i:\tilde{P}_{i}\left( x \right)<\tilde{P}_{i}(y)$. This implies that $P_{2}\left( x \right)<P_{2}(y)$, $P_{3}\left( x \right)<P_{3}(y)$. When instead of considering $\tilde{P}_{1}$ we consider $P_{1}$, it is possible that instead of performing task 1 better than $x$, $y$ performs task 1 the same as $x$ (This happens if both $x$ and $y$ are on $A_{1}$). Hence, $x$ is not Pareto optimal also relative to $P_{1},P_{2}$ and $P_{3}$.

Lemma: If $x\in T$ and $x\notin A_{1}^{^{\circ}}$, then $x$ is Pareto optimal.

Proof: Let $x\in T, x\notin A_{1}^{^{\circ}}$. If there was a point $y$ that dominated $x$, it would mean that $P_{i}\left( x \right)\leq P_{i}\left( y \right)$, with at least one proper inequality. $P_{2}\left( x \right)\leq P_{2}\left( y \right)$ or $P_{3}\left( x \right)\leq P_{3}\left( y \right)\Rightarrow\tilde{P}_{2}\left( x \right)\leq\tilde{P}_{2}\left( y \right)$ or $\tilde{P}_{3}\left( x \right)\leq\tilde{P}_{3}\left( y \right)$. If $P_{1}\left( x \right)<P_{1}\left( y \right)$, it means that $x\notin A_{1}$ and certainly $\tilde{P}_{1}\left( x \right)<\tilde{P}_{1}\left( y \right)$. For $\in\partial A_{1}$ $P_{1}\left( x \right)=P_{1}\left( y \right)\Rightarrow\tilde{P}_{1}\left( x \right)\leq\tilde{P}_{1}\left( y \right)$, and for $x\in A_{1}$ $P_{1}\left( x \right)=P_{1}\left( y \right)\Rightarrow\tilde{P}_{1}\left( x \right)=\tilde{P}_{1}\left( y \right)$. So, for $x\notin A_{1}^{^{\circ}}$, $P_{1}\left( x \right)=P_{1}\left( y \right)\Rightarrow\tilde{P}_{1}\left( x \right)\leq\tilde{P}_{1}\left( y \right)$. Thus, $P_{i}\left( x \right)\leq P_{i}\left( y \right)\Rightarrow\tilde{P}_{i}\left( x \right)\leq\tilde{P}_{i}\left( y \right)$, and $P_{i}\left( x \right)<\left( y \right)\Rightarrow\tilde{P}_{i}\left( x \right)<\left( y \right)$, so if $x$ is not Pareto optimal relative to ${\{P}_{i}\}$ it is not Pareto optimal relative to $\{\tilde{P}_{i}\}$. Since $x\in T$, it is Pareto optimal relative to $\{\tilde{P}_{i}\}$, and hence it is Pareto optimal relative to ${\{P}_{i}\}$.

Lemma: If $x\in T\cap A_{1}^{^{\circ}}$, then it is either on $P_{2,3}$ or it is not Pareto optimal.

Proof: Let $x\in T\cap A_{1}^{0}$. Assume $x$ is not on $P_{2,3}$. We would like to show that in this case, $x$ is not Pareto optimal. There is $\epsilon_{1}>0$ such that $\bar{B_{0}(x,\epsilon_{1})}\subseteq A_{1}$, since $x\in A_{1}^{^{\circ}}$. There is $\epsilon_{2}>0$ such that $\bar{B_{0}(x,\epsilon_{2})}$ does not intersect $P_{2,3}$ since $x$ is not on that front (It was shown in Appendix 7 that the Pareto front is closed. The argument brought there applies to any number of dimensions). Let $\epsilon=min(\epsilon_{1},\epsilon_{2})$. $\bar{B_{0}(x,\epsilon)}\subseteq A_{1}$ and does not intersect $P_{2,3}$. $x$ is not Pareto optimal relative to $P_{2}$ and $P_{3}$ since it is not on $P_{2,3}$. We saw in Appendix 1 that if x is not Pareto optimal (when the performance functions decay we a norm from a point archetype), there is a direction such that points in this direction close enough to the point dominate it. This means that there is $y\in\bar{B_{0}(x,\epsilon)}$ that performs tasks 2 and 3 better than $x$ does. $y\in\bar{B_{0}\left( x,\epsilon\right)}\subseteq A_{1}\Rightarrow y\in A_{1}\Rightarrow y$ performs task 1 the same as $x$ does since they are both on the archetype of task 1 $\Rightarrow x$ is dominated by $y\Rightarrow x$ is not Pareto optimal.

Lemma: If $x\in P_{2,3}$, then $x$ is pareto optimal (even if it is on $A_{1})$

Proof: If $x\in P_{2,3}$ it means that no other point performs both task 2 and task 3 better than it, which in turn means that no other point performs all 3 tasks better than it, without regard to performance 1. Hence, $x$ is Pareto optimal.

When considering the case of performance functions that decay with Euclidean norm, we get a circular shaped archetypal region, and a resulting Pareto front as depicted in Fig. 12 in the main text.

For the special case where $a_{2}=a_{3}$, $P_{2}=P_{3}$, we effectively get the case of 2 tasks, one maximized in a region and one at a single point. Such a case, where $P_{1}$ and $P_{2}/P_{3}$ decay with different inner product norms, is depicted in Fig. 11 in the main text.

# Appendix 9

## Bounds on the Pareto front for general performance functions show that normally it is located in a region close to the archetype

We would like to find conditions as general as possible under which the Pareto front will be constrained to an area close to the line between the archetypes. We consider the case of more general performance functions, and of archetypes that are regions instead of points.

We start with the case of 2 point-like archetypes: $a_{1}$ and $a_{2}$. Denote by $C_{2}^{1}$ the contour of the first performance function, $P_{1}$, on which lies $a_{2}$. Define $B_{2}^{1}$ as the set of points in which performance $1$ is greater or equal to its value on the contour (i.e. $B_{2}^{1}=\{x|P_{1}\left( x \right)\geq P_{1}\left( a_{2} \right)\}$). In the same manner, denote by $C_{1}^{2}$ the contour of the second performance function, $P_{2}$, on which lies $a_{1}$, and $B_{1}^{2}=\{x|P_{2}\left( x \right)\geq P_{2}\left( a_{1} \right)\}$.

Claim: Under these conditions, the Pareto front is bounded in $B_{2}^{1}\cap B_{1}^{2}$.

Proof: Let $z\notin B_{2}^{1}\cap B_{1}^{2}$. There are 3 options: $z\in{B_{2}^{1}}/{B_{1}^{2}},z\in{B_{1}^{2}}/{B_{2}^{1}}, z\notin B_{1}^{2}\cup B_{2}^{1}$.

If $z\in{B_{2}^{1}}/{B_{1}^{2}}$, it is dominated by $a_{1}$ as $P_{2}\left( z \right)<P_{2}(a_{1})$ since $a_{2}\in B_{1}^{2}$ and $P_{1}\left( z \right)<P_{1}(a_{1})$ since $a_{1}$ is the archetype of task 1 so it maximizes the performance of that task. The same argument shows that if $z\in{B_{1}^{2}}/{B_{2}^{1}},$ then $z$ is dominated by $a_{2}$. If $z{\notin B}_{1}^{2}\cup B_{2}^{1}$, it is dominated by any point in $B_{1}^{2}\cup B_{2}^{1}$. Hence, the Pareto front is restricted to the area $B_{1}^{2}\cap B_{2}^{1}$.

In this area the Pareto front does not have to be connected. Such scenario can arise when the performance functions are not monotonic, or do not depend monotonically on strictly concave functions (See Fig S5).

Consider now the case of $k$ point-like archetypes. Denote by $C_{j}^{i}$ the contour of the $i^{th}$ performance function, $P_{i}$, on which $a_{j}$ lies. Denote by $B_{j}^{i}$ the set of points in which performance $i$ is greater or equal to its value on the contour ($B_{j}^{i}=\{x|P_{i}\left( x \right)\geq P_{i}\left( a_{j} \right)\}$). For each $j,$ the Pareto front must lie in $\bigcup_{\begin{aligned} i=1, \\ i\neq j \end{aligned}}^{k} B_{j}^{i}$, since each point outside this set is dominated by $a_{j}$. If $x\notin\bigcup_{\begin{aligned} i=1, \\ i\neq j \end{aligned}}^{k} B_{j}^{i}\Rightarrow\forall i\neq j: x\notin B_{j}^{i}\Rightarrow x$ performs task $i$ for $i\neq j$ worse than any phenotype in $B_{j}^{i}$. As ${\forall i: a}_{j}\in B_{j}^{i}$, $x$ performs task $i$ worse than $a_{j}$. $x$ performs task $j$ worse than $a_{j}$. Since $a_{j}$ is the archetype of task $j$, $x$ performs task $j$ worse than $a_{j}$ $\Rightarrow$ $x$ performs all tasks worse than $a_{j}\Rightarrow x$ is not Pareto optimal.

Conclusion: $x\in$ Pareto front $\Rightarrow x\in\bigcup_{\begin{aligned} i=1, \\ i\neq j \end{aligned}}^{k} B_{j}^{i}$ for every $j$.

$\Rightarrow$Pareto front $\subseteq\bigcap_{j=1}^{k} \bigcup_{\begin{aligned} i=1, \\ i\neq j \end{aligned}}^{k} B_{j}^{i}$

Consider again the case of 2 archetypes. Assume now that instead of a point, one of the archetypes is a region. We would like to find a bound to the Pareto front in this scenario.

We assume the first archetype $A_{1}$ is a closed bounded region. Let $x_{1,2}$ be the point on $A_{1}$ with the best performance of task 2. Since $A_{1}$is compact and $P_{2}$ is continuous, such points exist. As before, let $B_{2}^{1}$ be the set of points outperforming $a_{2}$ in task 1, $B_{2}^{1}=\{x|P_{1}\left( x \right)\geq P_{1}\left( a_{2} \right)\}$. Let $B_{1}^{2}$ be the set of points outperforming $x_{1,2}$ in task 2, $B_{1}^{2}=\{x|P_{2}\left( x \right)\geq P_{2}\left( x_{1,2} \right)\}$. Take a point $z$ outside of $B_{2}^{1}\cap B_{1}^{2}$. Again there are 3 options: $z\in{B_{1}^{2}}/{B_{2}^{1}}, z\in{B_{2}^{1}}/{B_{1}^{2}}, z\notin B_{2}^{1}\cup B_{1}^{2}$. If $z\in{B_{1}^{2}}/{B_{2}^{1}}$, then $P_{1}\left( z \right)<P_{1}(a_{2})$ since $a_{2}\in C_{2}^{1}$ and $z\notin C_{2}^{1}$, and $P_{2}\left( z \right)<P_{2}(a_{2})$ since $a_{2}$ is the archetype of task 2 so it maximizes the performance of that task. It means that $z$ is dominated by $a_{2}$, and hence it is not Pareto optimal. If $z\in{B_{2}^{1}}/{B_{1}^{2}},$ then $P_{2}\left( z \right)<P_{2}(x_{1,2})$ since $x_{1,2}\in B_{1}^{2}$ and $z\notin B_{1}^{2}$, and $P_{1}\left( z \right)\leq P_{1}(x_{1,2})$ since $x_{1,2}$ is on $A_{1}$, the archetype of task 1 so it maximizes the performance of that task. Therefore, $z$ is dominated by $x_{1}^{2}$ and is not Pareto optimal. If $z\notin B_{2}^{1}\cup B_{1}^{2}$, it is dominated by any point in $B_{2}^{1}\cup B_{1}^{2}$. Hence, the Pareto front is restricted to the area $B_{2}^{1}\cap B_{1}^{2}$ in this case as well.

We further ask what happens in the case where there are 3 archetypes – the first archetype, $A_{1}$ is a region, and the 2 other are points - $a_{2},a_{3}$. Let $x_{1,2}$ and $x_{1,3}$ be the point on $A_{1}$ with the best performance of task 2 and 3, respectively. Let $B_{1,2}^{2},B_{1,2}^{3}$ be the set of points that outperform $x_{1,2}$ in task 2 and 3, respectively. Let $B_{1,3}^{2},B_{1,3}^{3}$ be the set of points that outperform $x_{1,3}$ in task 2 and 3, respectively. By definition - $\forall x\in B_{1,i}^{j},z\notin B_{1,i}^{j}:P_{j}\left( z \right)<P_{j}\left( x \right)$.

In that case, the Pareto front must be contained in $B_{1,2}^{2}\cup B_{1,2}^{3}$: Any point outside of this set is dominated by $x_{1,2}$, since $x_{1,2}$ is contained in both contours, it performs tasks 2 and 3 better than any point outside of those contours, and task 1 better than any other point since it is on $A_{1}$. The same argument applies to $B_{1,3}^{2}\cup B_{1,3}^{3}$. From the same arguments brought earlier, the front is contained in $B_{2}^{1}\cup B_{2}^{3}$ and in $B_{3}^{1}\cup B_{3}^{2}$. Hence, the front is bounded in ($B_{1,2}^{2}\cup B_{1,2}^{3})\cap(B_{1,3}^{2}\cup B_{1,3}^{3}) \cap(B_{2}^{1}\cup B_{2}^{3})$ $\cap$ ($B_{3}^{1}\cup B_{3}^{2}$)

If all 3 archetypes are regions, each term can be replaced by ($B_{j}^{i}\cup B_{j}^{k})\to(B_{j,i}^{i}\cup B_{j,i}^{k})\cap(B_{j,k}^{i}\cup B_{j,k}^{k})$, resulting in the Pareto front bounded by $\bigcap_{j=1}^{3} \bigcup_{i\neq k\neq j,i,k=1}^{3} (B_{j,i}^{i}\cup B_{j,i}^{k})\cap(B_{j,k}^{i}\cup B_{j,k}^{k})$

Conclusion: The Pareto Front





**Figure S5: The Pareto front does not have to be connected for non monotonic performance functions. A and B:** A plot of 2 chosen non-monotonic performance functions, $P_{1}=4.1ⅇ^{\frac{-(x^{2}+y^{2})}{0.1}}+4.9ⅇ^{\frac{-{({(x-1)}^{2}+y^{2})}^{2}}{.5}}+5ⅇ^{\frac{-{({(x)}^{2}+{(y-1)}^{2})}^{2}}{.5}}+5.1ⅇ^{\frac{-{({(x)}^{2}+{(y+1)}^{2})}^{2}}{.5}}+5.2ⅇ^{\frac{-{({(x+1)}^{2}+y^{2})}^{2}}{.5}}$

and $P_{2}= 4.1ⅇ^{\frac{-({(x-1)}^{2}+{(y-1)}^{2})}{0.1}}+ 4.9ⅇ^{\frac{-{({(x-1-1)}^{2}+{(y-1)}^{2})}^{2}}{.5}}+5ⅇ^{\frac{-{({(x-1)}^{2}+{(y-1-1)}^{2})}^{2}}{.5}}+2ⅇ^{\frac{-{({(x+1-1)}^{2}+{(y-1)}^{2})}^{2}}{.5}}+5.1ⅇ^{\frac{-{({(x-1)}^{2}+{(y+1-1)}^{2})}^{2}}{.5}}+3ⅇ^{\frac{-{({(x-1.9)}^{2}+{(y+1.3)}^{2})}^{2}}{.2}}$.

**C:** The Pareto front related to those performance functions is not connected. $C_{2}^{1}$ - the contour of performance function 1 going through $a_{2}$, the archetype of task 2, is in thick purple. $C_{1}^{2}$ - the contour of performance function 2 going through $a_{1}$ , the archetype of task 1, is in thin blue. $a_{1}$ and $a_{2}$ are red dots. The Pareto front is plotted in red. It can be seen that it is not connected.

#

# Appendix 10

## The Pareto front of r strongly concave performance functions is a connected set of Hausdorff dimension of at most r-1.

We would like to calculate the Pareto front of a system that needs to perform r tasks in an n-dimensional trait space V = $\mathcal{R}^{n}$. Each performance function $P_{i}(v)$ has a single maximum - the archetype $v_{i}^{*}$. $P_{i}$ is assumed to be smooth and strongly concave.

In appendix 1 we’ve shown that for such a system, for a Pareto optimal point *v* there exists a convex linear combination of that equals zero.

For performance functions $P_{i}(v)$ that decay with inner product norms, we’ve shown that if there exists a convex linear combination of that equals zero, *v* is Pareto optimal.

This remains true for strongly concave performance functions:

Lemma 1: For strongly concave $\{P_{i}\}$, if there exists a convex linear combination of that equals zero, then *v* is Pareto optimal

Proof: By assumption, there exist ${\{\alpha_{i}\geq0\}}_{i=1}^{r}, \sum_{i=1}^{r} \alpha_{i}=1$ such that .

Consider the function $f= \sum_{i=1}^{r} \alpha_{i}P_{i}$. Since all $P_{i}$’s are strongly concave, all $\alpha_{i}$’s are positive and are not all zero, *f* is strictly concave. By our assumption , and together those imply that *v* maximizes *f*. Since *f* is monotonic with all $P_{i}$’s, this implies *v* is Pareto optimal.

Hence, *v* is Pareto optimal iff there is a convex combination of that equals zero.

In addition, for each convex set $\left\{ \alpha_{i} \right\}_{i=1}^{r}$, there exists a *v* such that . This is because each of the $P_{i}$ is strongly concave on $\mathcal{R}^{n}$, and hence decays to negative infinity. This implies that $\sum_{i=1}^{r} \alpha_{i}P_{i}$ decays to negative infinity, so it must attain a maximum. This maximum point *v* will satisfy . Also, from strict concavity, there will be only a single point *v* that satisfies this relation.

To conclude, we found that for every convex set $\left\{ \alpha_{i} \right\}_{i=1}^{r}$ there is a single point *v* such that , *v* is Pareto optimal, and those are all Pareto optimal points.

Hence, the set $\{\alpha_{1},\ldots\alpha_{r}|\alpha_{i}\geq0,\sum_{i=1}^{r} \alpha_{i}=1\}$ fully determines the Pareto front.

Denote by T := $\{\alpha_{1},\ldots\alpha_{r}|\alpha_{i}\geq0,\sum_{i=1}^{r} \alpha_{i}=1\}$ the unit (r-1)-simplex. $T\subset\mathcal{R}^{r-1}$. By the above considerations, we can define a function *h*:*T*$\to\mathcal{R}^{n}$ by assigning each {$\alpha_{1},\ldots\alpha_{r}\}$ with the maximal point of $\sum_{i=1}^{r} \alpha_{i}P_{i}$. By the above results, we know that *Im(T)* equals the Pareto front.

Lemma 2: *h* is continuously differentiable

Proof:

Take $\alpha:=\{\alpha_{1},\ldots,\alpha_{r}\}\in T\subset\mathcal{R}^{r-1}$. Take $v=h\left( \alpha\right)\in\mathcal{R}^{n}$.

$k\left( \alpha,v \right)=0$, where *k* is continuously differentiable.

Denote $k=\{k_{1},\ldots,k_{n}\}$

Then $\left( \begin{matrix} \frac{\partial k_{1}}{\partial y_{1}} & \cdots& \frac{\partial k_{1}}{\partial y_{n}} \\ \vdots& \ddots& \vdots\\ \frac{\partial k_{1}}{\partial y_{n}} & \cdots& \frac{\partial k_{n}}{\partial y_{n}} \end{matrix} \right)=\sum_{i=1}^{r} \alpha_{i}\nabla^{2}P_{i}$.

Since $P_{i}$ is strongly convex, $\nabla^{2}P_{i}$ is positive definite for every *i,* and hence $\sum_{i=1}^{r} \alpha_{i}\nabla^{2}P_{i}$ is positive definite, and hence not singular.

According to the implicit function theorem, this implies that there exists an open environment *U* of $\alpha$ (*U* itself is not necessarily contained in *T*) , an open environment *V* of *v*, and a unique continuously differentiable function $g:U\to V$ such that $\forall x\in U$: $k\left( x,g(x) \right)=0$.

However, we know that $\forall\alpha^{'}\in T:\exists$ a *single* *v’* such that ${k(\alpha}^{'},v)=0$. This implies that $h|_{U\cap T}=g|_{T}\Rightarrow h$ is continuously differentiable at $\alpha$.

This is true for every $\alpha\in T$, and hence *h* is continuously differentiable on *T.*

Corollary: For strongly concave and smooth performance functions, the Pareto front is connected.

Proof: We saw that the Pareto front is the image of *T* under *h. T* is connected (as the unit (r-1)-simplex in $\mathcal{R}^{r})$ and *h* is continuous, and hence the Pareto front is connected.

Corollary: The Pareto front has Hausdorff dimension of at most r-1

Proof: This follows as *T* has Hausdorff dimension of r-1 (as the unit (r-1)-simplex$)$ and *h* is $C^{1}$.

Note that *v* is Pareto optimal relative to $P_{1},\ldots,P_{r}\Leftrightarrow$ $\nexists v^{'}\neq v s.t \forall i:P_{i}\left( v \right)<P_{i}(v^{'})$ $\Leftrightarrow$ $\nexists v^{'}\neq v s.t \forall i:f(P_{i}\left( v \right))<f(P_{i}\left( v' \right))$ where $f\mathcal{:R\to R}$ is a monotonic increasing function $\Leftrightarrow$ *v* is Pareto optimal relative to $f\circ P_{1},\ldots,f\circ P_{r}$

Corollary: The Pareto front relative to $P_{1},\ldots,P_{r}$, where each $P_{i}$ is a smooth monotonically increasing function of a strongly concave function is continuous and has a maximal Hausdorff dimension of *r*-1.

# References

1. Grant PR, Abbott I, Schluter D, Curry RL, Abbott LK (1985) Variation in the size and shape of Darwin’s finches. *Biological Journal of the Linnean Society* 25:1-39.

2. Barber CB, Dobkin DP, Huhdanpaa H (1996) The quickhull algorithm for convex hulls. *ACM Trans Math Softw* 22:469–483.

3. Klee V, Laskowski MC (1985) Finding the smallest triangles containing a given convex polygon. *Journal of Algorithms* 6:359-375.

4. M. Gerstenhaber (1951) Theory of convex polyhedral cones, Chap. XVIII of Cowles Commission Monograph No. 13, Activity analysis of production and allocation, ed. T. C. Koopmans, Wiley, New York,.
